# Effectiveness of physio-cognitive dual-task training on improving global cognition, health-related quality of life, and physical outcomes among older adults with neurocognitive disorders: an umbrella review

**DOI:** 10.1093/ageing/afag061

**Published:** 2026-03-27

**Authors:** Calvin Wei Jie Chern, Ling Jie Cheng, Glenys Shu Wen Chiang, Siat Yee Yap, Thuy Anh Giang, Siew Tiang Lau

**Affiliations:** National University of Singapore, Alice Lee Centre for Nursing Studies, Yong Loo Lin School of Medicine, Singapore, Singapore; National University of Singapore, Alice Lee Centre for Nursing Studies, Yong Loo Lin School of Medicine, Singapore, Singapore; University of Oxford, National Perinatal Epidemiology Unit, Nuffield Department of Women’s & Reproductive Health, Oxford, England, United Kingdom of Great Britain and Northern Ireland; Ng Teng Fong General Hospital, Nursing Department, Singapore, Singapore; National University of Singapore, Alice Lee Centre for Nursing Studies, Yong Loo Lin School of Medicine, Singapore, Singapore; Khoo Teck Puat Hospital, Department of Rehabilitation Services, Singapore, Singapore; National University of Singapore, Alice Lee Centre for Nursing Studies, Yong Loo Lin School of Medicine, Singapore, Singapore

**Keywords:** aged, cognitive training, neurocognitive disorders, quality of life, rehabilitation, systematic review, older people

## Abstract

**Background:**

Physio-cognitive dual-task training (PCDT), combining physical and cognitive tasks, is a promising approach. However, clarity regarding its effectiveness and evidence credibility remains limited.

**Objective:**

To evaluate PCDT effectiveness on global cognition, health-related quality of life, and physical outcomes (activities of daily living, gait, balance) in older adults with neurocognitive disorders, assess review quality and evidence certainty, and explore moderator effects.

**Methods:**

Eight databases and grey literature were searched to 31 December 2024. Two reviewers independently screened, extracted data, and assessed review quality (AMSTAR-2) and evidence certainty (GRADE). Meta-level and study-level meta-analyses were conducted. Subgroup analyses and meta-regression explored moderator effects. PROSPERO: CRD42024622115.

**Results:**

Seventeen reviews with 47 unique meta-analyses involving 81 unique studies were included. Meta-level analyses indicated small significant improvements across outcomes except health-related quality of life. Study-level analyses, correcting for overlapping primary studies, confirmed significant benefits for global cognition and health-related quality of life; however, physical outcome effects were non-significant. Prediction intervals for all outcomes were non-significant. Simultaneous PCDT and higher weekly frequency showed greater benefits. Participants with dementia benefited less than those with mild cognitive impairment. Age was not a significant moderator. Most reviews were low or critically low quality, and evidence certainty was low.

**Conclusions:**

PCDT is potentially associated with improvements in cognitive, physical, and quality-of-life outcomes among older adults with neurocognitive disorders. However, prediction intervals suggest effectiveness uncertainty, and heavy sample weighting toward prodromal stages warrants caution. PCDT may not be indicated for cognitive improvement in established dementia. High-quality reviews are urgently needed.

## Introduction

The inevitability of an ageing population [1] will increase the prevalence of those with neurocognitive disorders, a major health detriment [2]. Dementia and Alzheimer’s disease progressively impair cognitive function, reducing the ability to perform daily tasks [3]. Mild Cognitive Impairment is an intermediary status between normal cognition and dementia [4], with the potential to deteriorate into the latter and cause further functional decline by impairing dual-task activities [3, 5] (Appendix B1). When left unchecked, the collective impairment in activities of daily living [6] and impaired gait and balance [7] will contribute to a poorer health-related quality of life [8].

Physio-cognitive dual-task training (PCDT) has gained interest as a rehabilitation strategy due to its simultaneous or sequential simulation of real-world multitasking demands, offering potential advantages that cannot be achieved through single-task training [9, 10]. Several meta-analyses have evaluated the effects of PCDT on physical and cognitive outcomes, with generally positive, though inconsistent, findings across cognition, physical function, and health-related quality of life [11, 12]. Furthermore, only one review conducted meta-regression to explore the influence of moderators on the intervention effects [13], while others have relied on narrative synthesis. Although prior umbrella reviews exist, none have provided a comprehensive synthesis specific to older adults with neurocognitive disorders, lacking clearly defined objectives or outcomes [14, 15], rendering their consensus on PCDT effectiveness inadequate.

Thus, a robust and methodologically rigorous umbrella review is warranted to answer the following questions:

Research Question 1: What is the effectiveness of PCDT on global cognition, health-related quality of life, and physical outcomes (activities of daily living, gait, balance) among older adults with neurocognitive disorders, and their associated review quality and strength of evidence?

Research Question 2: What moderators influence the effectiveness of PCDT in this population?

## Methods

### Protocol and registration

This umbrella review’s methodology followed the Joanna Briggs Institute manual recommendation for umbrella reviews [16], while the reporting followed the Preferred Reporting Items for Overviews of Systematic Reviews [17] (Appendix A1) and Preferred Reporting Items for Systematic Reviews and Meta-Analyses [18] (Appendix A2). The study protocol was registered in the International Prospective Register of Systematic Reviews (PROSPERO) database (CRD42024622115).

### Eligibility criteria

We included reviews that (i) included participants with at least a mean age of 50 years old due to a higher general risk and prevalence of cognitive impairment [2]; (ii) diagnosed or clinically evaluated to have age-related neurocognitive disorders of mild cognitive impairment and dementias of similar aetiologies [19, 20]; (iii) dual-task interventions combining physical and cognitive interventions (simultaneous or sequential); (iv) comparators included active controls, passive controls, or a combination of both; (v) reported one or more of the primary outcomes; (vi) systematic analyses and meta-analyses from inception to 31 December 2024; and (vii) reviews published in the English language.

Reviews with neurocognitive disorders of non-age-related aetiologies such as vascular dementia [21, 22], were excluded. The full criteria are detailed in Appendix A3.

### Search strategy and eligibility criteria

A preliminary search of PROSPERO and PubMed Clinical Queries was conducted to confirm that no concurrent umbrella reviews were being done on this topic of interest. A university medical librarian was consulted to refine the search strategy. A three-step search strategy was employed following the Cochrane Handbook for Systematic Reviews [23], minimising selection bias risk, Systematic reviews and meta-analyses were searched for in eight databases: PubMed, Embase, CINAHL Complete, The Cochrane Library, Scopus, Web of Science Core Collection, PsycINFO, and ProQuest Theses and Dissertations (Appendix A4). Grey literature sources including GreySource, CogPrints, and the first ten pages of Google Scholar were hand searched. Reference lists of included reviews were also hand searched for further relevant systematic reviews and meta-analyses. Results and references were imported into EndNote 21.0 [24] for record management.

### Study selection and data extraction

Two reviewers (CWJC and GSWC) independently screened titles and abstracts, retrieved full texts of potentially eligible reviews, assessed them against the inclusion criteria, and data extracted using a pre-specified form (Appendix A5). Corresponding authors were contacted to clarify any missing or ambiguous data. A complete case analysis was conducted for data that remained unavailable. Discrepancies were resolved by consensus, and if needed, through discussion with a third reviewer (CLJ). Cohen’s Kappa (*κ*) statistics were used to calculate inter-rater reliability [25], and the levels were as follows: None: ≤0; None to slight: 0.01–0.2; Fair: 0.21–0.40; Moderate: 0.41–0.60; Substantial: 0.61–0.80; Almost perfect: 0.81–1.00.

### Quality assessment of systematic reviews

The methodological quality of reviews were assessed using the Assessment of Multiple Systematic Reviews 2 (AMSTAR-2) [26], Two independent reviewers (CWJC and GSWC) rated the quality level of each review (high, moderate, low, critically low) based on 16 items, of which seven were critical domains (Appendix A6). The third reviewer (CLJ) was consulted to resolve any remaining disputes.

### Overlapping studies

To account for overlapping studies included between the reviews, the below formulas by Pieper *et al*. [27] were used to measure the degree of overlap using percentage overlaps, covered area (CA) and corrected covered area (CCA):


\begin{align*} \%\, overlaps&=\frac{\mathrm{N}\mathrm{umber}\ \mathrm{of}\ \mathrm{overlapped}\ \mathrm{studies}}{\mathrm{Total}\ \mathrm{of}\ \mathrm{primary}\ \mathrm{studies}},\nonumber\\ CA&=\frac{\mathrm{N}}{\mathrm{rc}}, CCA=\frac{\mathrm{N}-\mathrm{r}}{\mathrm{rc}-\mathrm{r}} \end{align*}

N = total number of included studies (including double count) in the reviews, r = number of primary studies, and c = number of included reviews. The degree of overlap was represented using the following CCA scores [27]: Slight overlap: 0%–5%; Moderate overlap: 6%–10%; High overlap: 11%–15%; Very high overlap: >15%.

### Meta-analyses of meta-analysed data and study-level data

Meta-analyses were conducted using R software [28] and the *metafor* package [29]. For meta-level data, effect sizes and their corresponding metrics were pooled in R using inverse variance weighting. To prevent overestimating effect sizes owing to overlapping studies between reviews [27], meta-analyses of meta-level and study-level data were performed and compared. For study-level data, unique primary studies that met the eligibility criteria and measured relevant outcomes had their corresponding means and standard deviations extracted for effect size calculation. Random-effects meta-analysis employed the Hartung-Knapp-Sidik-Jonkman method as it reliably results in adequate error rates, particularly in small sample sizes and when high between-study heterogeneity exists [30]. Hedges’ *g* was used to accurately estimate the aggregated effect sizes [31] and was interpreted as: small ≥0.2; moderate ≥0.5; large ≥0.8; extremely large ≥1.0.

Inter-review and inter-study heterogeneities were assessed using Cochran’s Q and *I*^2^ statistics [32]. An *I*^2^ statistic of ≥50% will indicate heterogeneity [23]. Subgroup analyses and meta-regression were conducted to compare the effectiveness of PCDT moderators. For subgroup analyses, the following were split into categorical groups for effect size comparisons [33]: neurocognitive disorder nature, PCDT methodology, training duration, session duration, and weekly training frequency. Meta-regression was performed to measure if age influenced effect sizes using regression coefficient (*β*) for estimating effect size, with *P* < .05 indicating a significant threshold [34]. Publication bias was evaluated using funnel plots and Egger’s test, where *P* < .10 indicates a statistically significant small-study effect [35]. A biostatistician was consulted to review the accuracy of the findings.

### Evaluation of certainty of evidence

The certainty of evidence for meta-level findings was assessed using the Grading of Recommendations Assessment, Development and Evaluation (GRADE) approach for systematic reviews and meta-analyses [36]. We adjusted the review-level risk of bias using the seven critical domains of AMSTAR-2 instead of the proposed four critical AMSTAR domains [26, 37], as this was published before AMSTAR-2’s inception. Downgrading criteria included imprecision, study-quality risk of bias, inconsistency, and review-quality risk of bias (Appendix A7.1, A7.2) as they were deemed key measurements [36], while indirectness was qualitatively and quantitatively assessed without downgrading [38]. Two independent reviewers (CWJC and GSWC) performed the assessments. Any disagreements were resolved through discussion with a third reviewer (CLJ).

## Results

### Review, and primary study selection, and review characteristics

**Figure 1:**
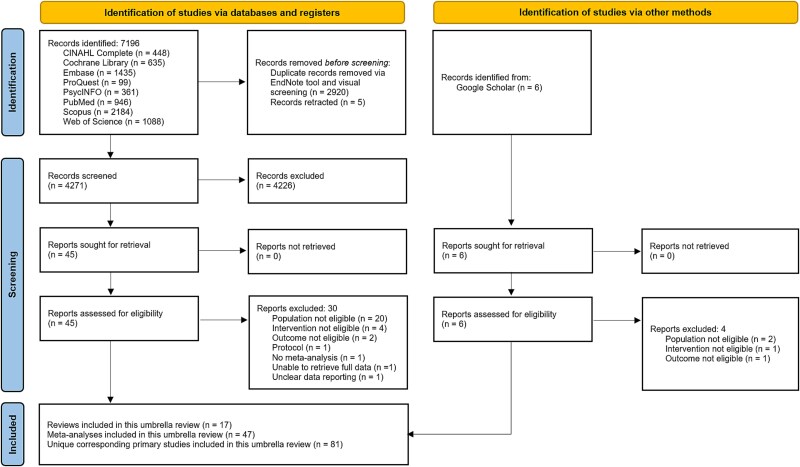
PRISMA 2020 flow diagram of review selection for umbrella review.

The search identified a total of 7202 records, of which the full texts of 51 records were assessed (Figure 1). After 34 records were excluded (Appendix A8), a total of 17 reviews [11–13, 39–52] with 47 unique meta-analyses, and 81 corresponding unique primary studies were included for meta-analyses comparisons. Study-level meta-analyses further excluded certain studies for reasons listed in Appendix A9. Inter-rater agreement was almost perfect for review selection (*κ* = 0.94), AMSTAR-2 assessment (*κ* = 0.98), and GRADE credibility of evidence (*κ* = 0.90), while substantial for data extraction (*κ* = 0.80). The 17 reviews included 26 591 participants (Mild cognitive impairment = 20 347, 76.52%) from 306 primary studies (Table 1, Table 2). Of the 17 reviews, 174 primary studies with simultaneous PCDTs (*n* = 99, 56.90%) and sequential PCDTs (*n* = 75, 43.10%) met this umbrella review’s eligibility criteria, not accounting for overlapping. Review affiliations and reported fundings are in Appendix A10. The between-studies overlaps for the 17 reviews measuring outcomes relevant to this umbrella review was 46.91%, CA was 0.13, and CCA was moderate at 7.17% (Appendix A11.1).

**Table 1:** Summary of systematic reviews and quality scores of included reviews.

References	Objectives	Sample size/Nature	Number of primary studies	Primary studies that met this UR’s eligibility	Comparator (passive, active, both)	Outcomes (Based on this UR’s eligibility)	AMSTAR-2 score
Ali et al. (2022) [39]	To assess the effects of dual-task training on cognitive and motor functions in older adults at various stages of cognitive decline.	Total: 2221 (MCI:1176 MCI; Dementia/ad: 635; Others: 410)	21	12 (Sim: 8; Seq: 4)	Passive care, active care	Global cognition: MCI & Dementia ↑. ad ↓; Balance ↑	Low
Cai et al. (2023) [40]	To assess the effect of exergaming intervention in MCI and dementia.	Total: 516 (MCI: 166; Dementia/ad: 350)	10	Sim: 9	Passive care, active care	Global cognition: MCI + Dementia ↑; ADL ↔; Gait ↔; Balance ↑; HRQoL ↔	Critically Low
Cai et al. (2024) [41]	To compare the effects of single-task and multi-task exergames on the cognitive ability of the elderly with MCI.	MCI: 526	11	Sim: 7	Passive care, active care	Global cognition: MCI ↑	Low
Chan et al. (2024) [42]	To examine the effect of exergaming on cognitive functions, specifically the type and training duration.	Total: 1152 (MCI: 773; Dementia: 379)	20	13 (Sim: 11; Seq: 2)	Passive care, active care, both	Global cognition: MCI + Dementia ↑	Moderate
Gómez-Soria et al. (2022) [43]	To assess the effects of two-component MNPI on global cognition and cognitive functions and to compare the degree of efficacy between the two interventions.	MCI: 592	8	3 (Sim: 1; Seq: 2)	Passive care, active care, both	Global cognition: MCI + aMCI ↑; ADL ↑; HRQoL ↑	Low
Han et al. (2022) [11]	To evaluate the effects of combined intervention on cognition in older adults with and without MCI.	Total: 825 (MCI: 434; Others: 391)	17	8 (Sim: 5; Seq: 3)	Passive care	Global cognition: MCI ↑	Critically Low
Hong et al. (2024) [13]	To evaluate the effectiveness of PCDT intervention on cognitive function, physical performance, ADL and QoL in pre-ageing and older adults with NCDs.	Total: 2256 (MCI: 1245; Dementia: 1011)	26	21 (Sim: 6; Seq: 15)	Passive care, active care	Global cognition: MCI + Dementia ↑; ADL ↔; Gait ↔; HRQoL ↑	High
Karssemeijer et al. (2017) [44]	To assess combined physical and cognitive intervention effects on cognitive domains of memory, executive function/attention, ADL, and mood.	Total: 742 (MCI: 267; Dementia/ad: 271; MCI + Dementia/ad: 204)	10	10 (Sim: 2; Seq: 8)	Passive care, active care	Global cognition: MCI + Dementia ↑; ADL ↑	Low
Li et al. (2022) [45]	To evaluate the effects of combined TaiChi and cognitive interventions on older adults.	Total: 979 (MCI: 318; Dementia: 181; Others: 480)	9	5 (Sim: 2; Seq: 3)	Passive care, active care	Global cognition: MCI + aMCI ↑; HRQoL ↔	Critically Low
Liu et al. (2023) [46]	To assess the comparative effectiveness of nonpharmacological interventions on cognitive function in older adults with MCI and to rank the interventions	MCI: 3319	28	9 (Sim: 3; Seq: 6)	Passive care, active care	Global cognition: MCI ↑	Low
Meng et al. (2022) [12]	To assess the efficacy of a combined intervention to improve cognition in older adults with MCI by comparing to a control group.	MCI + MCI Subtypes: 1337	16	12 (Sim: 6; Seq: 6)	Passive care, active care	Global cognition: MCI + aMCI ↑	Critically Low
Wati et al. (2024) [52]	To assess the efficacy of combining physical exercise and cognitive training to improve balance among older adults with cognitive impairment.	MCI: 255	4	3 (Sim: 1; Seq: 2)	Passive care, active care	Balance ↑	Critically Low
Xu et al. (2021) [47]	To evaluate the effectiveness of different types of interventions in improving global cognition among MCI patients.	MCI + MCI Subtypes: 5944	50	5 (Sim: 1; Seq: 4)	Passive care, active care	Global cognition: MCI ↑; aMCI ↔	Critically Low
Xue et al. (2023) [48]	To compare the effects of combined exercise and cognitive interventions on cognitive, psychological, functional outcomes, and health-related quality of life.	Total: 2910 (MCI: 1999; Dementia; 647; Others: 264)	29	20 (Sim: 7; Seq: 13)	Passive care, active care, both	Global cognition: MCI ↑; Dementia ↔; ADL ↔; HRQoL ↔	Low
Ye et al. (2024) [49]	To determine the effects of simultaneous dual-task training on cognitive function, physical function, and depression in older adults with MCI or dementia.	Total: 1477 (MCI: 813; Dementia: 664)	20	18 (Sim: 16; Seq: 2)	Passive care, active care	Global cognition: MCI ↑; Dementia ↔; ADL ↔; Gait ↑; Balance ↔	Low
Zhao et al. (2022) [50]	To compare and rank the relative effectiveness of different modes for exercise combined cognitive training in people with Alzheimer’s disease (ad) or Mild Cognitive Impairment [37].	Total: 1181 (MCI: 866; Dementia: 315)	16	11 (Sim: 7; Seq: 4)	Passive care, active care	Global cognition: MCI + ad ↑	Low
Zhu et al. (2021) [51]	To assess the effect of VR intervention on cognitive function (overall cognition, global cognition, attention, executive function, memory, visuospatial ability), and motor function (balance and gait).	Total: 359 (MCI: 317; Dementia: 42)	11	8 (Sim: 7; Seq: 1)	Passive care, active care	Global cognition: MCI + ad + Mild ad ↑; Gait ↔; Balance ↑	Critically Low

*Note.*  ad: Alzheimer’s dementia; ADL: Activities of daily living; aMCI: Amnestic mild cognitive impairment; AMSTAR-2: Assessment of Multiple Systematic Reviews 2; HRQoL: Health-related quality of life; MCI: Mild cognitive impairment; MNPI: Multi-component non-pharmacological interventions; NCD: Neurocognitive disorders; Others: Other populations that are included in the respective reviews, but not meeting this umbrella review’s eligibility criteria (Subjective cognitive decline, Vascular dementia, Parkinson’s, Healthy populations, etc.); PCDT: Physio-cognitive dual-task; QoL: Quality of life; Seq: Sequential; Sim: Simultaneous; UR: Umbrella review; VR: Virtual reality; ↑: Positive effect; ↓: Negative effect; ↔: Negligible Effect

**Table 2:** Characteristics of included reviews.

References	Review typology	Geographical location	Patient demographics		Search strategy				Quality appraisal instruments/Certainty of evidence
			Gender reported	Age range/Mean/Minimum	Databases searched	Search periods	Publication range of years	Publication language	
Ali et al. (2022) [39]	SR, MA	Asia, Europe, North America	Female & Male	67.5 to 87.2	6	Inception to 30 September 2020	2010 to 2020	English	Cochrane risk of bias/Unreported
Cai et al. (2023) [40]	SR, MA	Asia, Europe, North America, Middle East	Unreported	≥60	5	Inception to July 2022	2012 to 2022	English	Cochrane collaboration risk of bias/Unreported
Cai et al. (2024) [41]	SR, MA	Unreported	Female & Male	≥60	8	Inception to 1 April 2024	2014 to 2021	English	Cochrane risk of bias 2/Unreported
Chan et al. (2024) [42]	SR, MA	Asia, Europe, North America, Middle East	Female & Male	67 to 87	7	Inception to 31 March 2023	2012 to 2023	English	Cochrane risk of bias/Unreported
Gómez-Soria et al. (2022) [43]	SR, MA	Asia, Europe, South America	Female & Male	71.4	4	2010 to 18 January 2021	2011 to 2016	English	PEDro scale/Unreported
Han et al. (2022) [11]	SR, MA	Asia, Europe, North America	Female & Male	≥50	9	Inception to 1 November 2021	2002 to 2020	English	Cochrane collaboration risk of bias, PEDro scale/GRADE
Hong et al. (2024) [13]	SR, MA, Meta-regression	Asia, Europe, Oceania, Middle East	Female & Male	55.4 to 87.2	8	Inception to 1 August 2023	2011 to 2022	English	Cochrane risk of bias/GRADE
Karssemeijer et al. (2017) [44]	MA	Asia, Europe, North America, South America,	Female & Male	72.1	4	Inception to May 2017	2004 to 2017	English	Cochrane collaboration risk of bias/Unreported
Li et al. (2022) [45]	SR, MA	Asia, North America	Unreported	≥60	4	Inception to 12 November 2021	2012 to 2020	English	Cochrane risk of bias/Unreported
Liu et al. (2023) [46]	SR, Network MA	Africa, Asia, Europe, North America, South America	Female & Male	≥60	6	Inception to September 2022	2010 to 2022	English	Cochrane risk of bias/GRADE
Meng et al. (2022) [12]	MA	Asia, Europe, North America, Oceania, Middle East	Female & Male	73.33	6	Inception to February 2021	2013 to 2020	English	Cochrane collaboration risk of bias/Unreported
Wati et al. (2024) [52]	SR, MA	Asia, Europe, Oceania	Female & Male	65.9 to 87.5	4	Inception to 12 April 2023	2016 to 2020	English	Jaded Scale/Unreported
Xu et al. (2021) [47]	SR, MA, Network MA	Asia, Europe, Middle East, North America, South America	Female & Male	61.7 to 85.8	6	Inception to June 2020	2005 to 2020	English	Cochrane collaboration risk of bias/GRADE
Xue et al. (2023) [48]	SR, MA, Network MA	Asia, Europe, North America, Middle East Oceania	Unreported	55.4 to 87.2	10	Inception to 23 November 2022	2013 to 2022	English	Cochrane risk of bias/Unreported
Ye et al. (2024) [49]	SR, MA, Trial Sequential Analysis	Asia, Europe, Oceania, North America	Female & Male	63.8 to 87.2	7	Inception to December 2022	2010 to 2022	English	Cochrane risk of bias 2/Unreported
Zhao et al. (2022) [50]	SR, MA, Network MA	Asia, Europe, Middle East, North America, Oceania	Female & Male	67.07 to 87.2	7	Inception to May 2020	2013 to 2019	English	Cochrane risk of bias/GRADE
Zhu et al. (2021) [51]	SR, MA	Asia, Europe, North America	Female & Male	75.84	7	Inception to April 2020	2012 to 2020	English	Cochrane collaboration risk of bias, PEDro scale/Unreported

*Note*. GRADE: Grading of recommendations assessment, development and evaluation; MA: Meta-analysis; PEDRO scale: Physiotherapy evidence database scale; SR: Systematic review

### Methodological quality of systematic reviews

One review had a high quality rating [13], one review had a moderate quality rating [42], eight reviews had low quality ratings, and seven reviews had critically low quality ratings (Appendix A6). The most common methodological limitations were: failure to report sources of funding for included studies (*n* = 17) and omission of a list of excluded studies with justifications (*n* = 15).

### Global cognition outcomes

Meta-level meta-analysis from 16 reviews on PCDT’s effectiveness on global cognition found a small significant effect size (*g* = 0.39; 95% CI: 0.27, 0.52) (Table 3, Appendix B2.1). Between-studies overlaps for the 16 reviews was 53.73%, CA was 0.15, and CCA was moderate at 8.86% (Appendix A11.2). Study-level meta-analysis from 65 unique primary studies found a small significant effect size after removing overlapped studies (*g* = 0.47; 95% CI: 0.19, 0.75) (Table 3, Appendix B2.2), which was larger than the meta-level estimate, with substantial heterogeneity (*I*^2^ = 90.70%). Although there were no significant subgroup differences, moderate significant effect sizes were found within moderator subgroups such as older adults with mild cognitive impairment (*g* = 0.59; 95% CI: 0.18, 1.00), simultaneous PCDT (*g* = 0.74; 95% CI: 0.25, 1.22), training duration of ≤12 weeks (*g* = 0.70; 95% CI: 0.27, 1.14), session duration of ≤60 minutes (*g* = 0.68; 95% CI: 0.23, 1.12), and training frequency of ≥3 times per week (*g* = 0.55; 95% CI: 0.02, 1.08), while a training frequency of ≤2 times per week yielded a small significant effect size (*g* = 0.47; 95% CI: 0.22, 0.72) (Table 3, Appendix B2.3–B2.7, Appendix A12). The 95% prediction interval for both meta-level and study-level meta-analyses indicated that PCDTs may not improve global cognition compared to a comparator in future research under comparable conditions.

**Table 3:** Meta-level and study-level meta-analysis, subgroup analysis.

	Meta-level Data						Study-level Data				
Outcomes	MA	Effect Size (*g*) (95% CI) [Prediction Interval 95% CI]	*p*-value	*I* ^2^ (%)	Classification	Subgroups	No. of Studies (Total Sample)	Effect size (*g*) (95% CI), [Prediction Interval 95% CI]	*p*-value	*I* ^2^ (%)	Subgroup differences, *P*-value
Global Cognition	16	0.39 (0.27, 0.52) [−0.00, 0.79]	0.045	40.9	Global Analysis	**-**	65 (4586)	0.47 (0.19, 0.75) [−1.75, 2.69]	0.001	90.70	**-**
					NCD Nature	MCI	42 (2992)	0.59 (0.18, 1.00) [−2.07, 3.25]	0.004	92.60	.08
						Dementia	22 (1532)	0.27 (−0.03, 0.58) [−1.112, 1.67]	0.08	84.00	
					Intervention Type	Simultaneous	32 (1806)	0.74 (0.25, 1.22) [−2.05, 3.53]	0.003	89.50	.08
						Sequential	33 (2780)	0.24 (−0.05, 0.53) [−1.40, 1.88]	0.11	91.70	
					Training Duration	≤ 12 weeks	36 (1921)	0.70 (0.27, 1.14) [−1.91, 3.32]	0.002	89.4	.07
						> 12 weeks	29 (2665)	0.21 (−0.11, 0.52) [−1.52, 1.93]	0.21	90.70	
					Session Duration	≤ 60 minutes	35 (1942)	0.68 (0.23, 1.12) [−1.96, 3.31]	0.003	89.10	.19
						> 60 minutes	29 (2567)	0.31 (−0.01, 0.63) [−1.41, 2.03]	0.06	91.80	
					Frequency	≤ 2x per week	33 (2266)	0.47 (0.22, 0.72) [−0.91, 1.85]	0.002	86.50	.80
						≥ 3x per week	31 (2243)	0.55 (0.02, 1.08) [−2.48, 3.57]	0.04	92.80	
HRQoL	5	0.30 (−0.06, 0.67) [−0.68, 1.28]	0.11	55.3	Global Analysis	**-**	14 (764)	0.69 (0.26, 1.12) [−0.94, 2.33]	0.002	82.80	**-**
					NCD Nature	MCI	4 (285)	1.04 (0.67, 1.40) [−0.94, 2.33]	0.0001	0.00	.22
						Dementia	10 (479)	0.60 (0.02, 1.18) [−1.44, 2.65]	0.04	80.90	
					Intervention Type	Simultaneous	4 (202)	0.81 (−0.55, 2.17) [−3.97, 5.59]	0.25	91.10	.86
						Sequential	10 (562)	0.69 (0.33, 1.04) [−0.44, 1.81]	0.0002	71.90	
					Training Duration	≤ 12 weeks	10 (373)	0.83 (0.29, 1.37) [−0.99, 2.65]	0.003	76.90	.36
						> 12 weeks	4 (391)	0.42 (−0.25, 1.10) [−1.94, 2.79]	0.22	91.10	
					Session Duration	≤ 60 minutes	9 (391)	0.71 (0.11, 1.32) [−1.32, 2.74]	0.02	85.00	.89
						> 60 minutes	5 (373)	0.65 (0.06, 1.25) [−1.22, 2.52]	0.03	81.70	
					Training Frequency	≤ 2x per week	6 (341)	0.52 (0.06, 0.97) [−0.85, 1.88]	0.03	77.80	.41
						≥ 3x per week	8 (423)	0.86 (0.18, 1.55) [−1.40, 3.13]	0.01	86.70	
Physical—ADL	6	0.42 (0.18, 0.65) [0.06, 0.78]	0.0005	0	Global Analysis	**-**	16 (853)	0.39 (−0.22, 1.00) [−2.24, 3.01]	0.21	82.90	**-**
					NCD Nature	MCI	5 (258)	−0.00 (−0.45, 0.45) [−1.26, 1.26]	0.99	53.60	.25
						Dementia	11 (585)	0.58 (−0.30, 1.46) [−2.77, 3.93]	0.20	86.20	
					Intervention Type	Simultaneous	8 (372)	0.59 (−0.68, 1.87) [−3.90, 5.09]	0.36	90.80	.64
						Sequential	8 (481)	0.28 (0.00, 0.56) [−0.46, 1.03]	0.05	33.33	
					Training Duration	≤ 12 weeks	12 (565)	0.44 (−0.37, 1.26) [−2.75, 3.63]	0.29	84.70	.74
						> 12 weeks	4 (288)	0.27 (−0.35, 0.88) [−1.78, 2.31]	0.39	80.80	
					Session Duration	≤ 60 minutes	12 (565)	0.44 (−0.37, 1.26) [−2.75, 3.63]	0.29	84.70	.74
						> 60 minutes	4 (288)	0.27 (−0.35, 0.88) [−1.78, 2.31]	0.39	83.22	
					Training Frequency	≤ 2x per week	7 (430)	0.24 (−0.19, 0.67) [−1.09, 1.57]	0.27	78.50	.60
						≥ 3x per week	9 (423)	0.56 (−0.54, 1.66) [−3.41, 4.52]	0.32	86.50	
Physical—Gait	4	0.27 (0.11, 0.43) [−0.11, 0.66]	0.0008	39.2	Global Analysis	**-**	18 (981)	0.55 (−0.00, 1.10) [−1.87, 2.96]	0.051	56.40	**-**
					NCD Nature	MCI	13 (713)	0.69 (−0.09, 1.47) [−2.39, 3.76]	0.08	66.60	.46
						Dementia	5 (268)	0.37 (0.07, 0.67) [−0.29, 1.03]	0.02	0	
					Intervention Type	Simultaneous	16 (883)	0.60 (−0.03, 1.22) [−2.04, 3.23]	0.06	60.9	.41
						Sequential	2 (98)	0.28 (−0.12, 0.68) [−2.33, 2.90]	0.16	0	
					Training Duration	≤ 12 weeks	13 (550)	0.67 (−0.11, 1.45) [−2.43, 3.77]	0.09	67.80	.55
						> 12 weeks	5 (431)	0.42 (0.20, 0.65) [0.01, 0.83]	0.0002	0	
					Session Duration	≤ 60 minutes	11 (337)	0.73 (−0.22, 1.68) [−2.84, 4.29]	0.13	72.20	.55
						> 60 minutes	7 (644)	0.43 (0.25, 0.61) [0.10, 0.77]	0.0001	0	
					Training Frequency	≤ 2x per week	11 (726)	0.45 (0.26, 0.64) [0.04, 0.86]	0.0001	0	.58
						≥ 3x per week	7 (255)	0.89 (−0.65, 2.43) [−4.42, 6.20]	0.26	82.10	
Physical—Balance	5	0.37 (0.20, 0.55) [0.10, 0.65]	0.0001	0	Global Analysis	**-**	16 (643)	0.29 (−0.11, 0.70) [−1.32, 1.90]	0.15	67.20	**-**
					NCD Nature	MCI	10 (494)	0.23 (−0.03, 0.49) [−0.45, 0.91]	0.08	7.10	.93
						Dementia	6 (149)	0.28 (−0.80, 1.36) [−3.28, 3.84]	0.61	85.80	
					Intervention Type	Simultaneous	13 (467)	0.34 (−0.16, 0.84) [−1.54, 2.22]	0.18	72.50	.47
						Sequential	3 (176)	0.12 (−0.20, 0.45) [−0.73, 0.97]	0.47	0	
					Training Duration	≤ 12 weeks	13 (458)	0.37 (−0.12, 0.86) [−1.47, 2.22]	0.14	71.10	.24
						> 12 weeks	3 (185)	0.02 (−0.31, 0.34) [−0.90, 0.93]	0.92	0.00	
					Session Duration	≤ 60 minutes	12 (392)	0.37 (−0.17, 0.91) [−1.60, 2.34]	0.18	73.40	.41
						> 60 minutes	4 (251)	0.11 (−0.20, 0.42) [−0.64, 0.86]	0.48	0.00	
					Training Frequency	≤ 2x per week	10 (447)	0.17 (−0.10, 0.44) [−0.53, 0.87]	0.21	0.00	.69
						≥ 3x per week	6 (196)	0.39 (−0.67, 1.45) [−3.13, 3.91]	0.47	85.20	

*Note*. MA: Meta-analyses; MCI: Mild cognitive impairment; MMSE: Mini-mental state examination; MOCA: Montreal cognitive assessment; NCD: Neurocognitive disorders

### Health-related quality of life outcomes

Meta-level meta-analysis from five reviews on PCDT’s effectiveness on health-related quality of life found a small non-significant effect size (*g* = 0.30; 95% CI: −0.06, 0.67) (Table 3, Appendix B3.1). Between-studies overlaps for the five reviews was 14.29%, CA was 0.23, and CCA was slight at 3.33% (Appendix A11.3). Study-level meta-analysis from 14 unique primary studies found a moderate significant effect size after removing overlapped studies (*g* = 0.69; 95% CI: 0.26, 1.12) (Table 3, Appendix B3.2), which was larger than the meta-level estimates, with substantial heterogeneity (*I*^2^ = 82.80%). Although there were no significant subgroup differences, large significant effect sizes were found within moderator subgroups such as older adults with mild cognitive impairment (*g* = 1.04; 95% CI: 0.67, 1.40), training durations of ≤12 weeks (*g* = 0.83; 95% CI: 0.29, 1.37), and training frequencies of ≥3 times per week (*g* = 0.86; 95% CI: 0.18, 1.55), while moderate significant effect sizes were found for older adults with dementia (*g* = 0.60; 95% CI: 0.02, 1.18), sequential PCDT (*g* = 0.69; 95% CI: 0.33, 1.04), session duration of ≤60 minutes (*g* = 0.71; 95% CI: 0.11, 1.32) and > 60 minutes (*g* = 0.65; 95% CI: 0.06, 1.25), and training frequencies of ≤2x per week (*g* = 0.52; 95% CI: 0.06, 0.97) (Table 3, Appendix B3.3–B3.7, Appendix A12). The 95% prediction interval for both meta-level and study-level meta-analyses indicated that PCDTs may not improve health-related quality of life compared to a comparator in future research under comparable conditions.

### Physical—activities of daily living outcomes

Meta-level meta-analysis from six reviews on PCDT’s effectiveness on activities of daily living found a small significant effect size (*g* = 0.42; 95% CI: 0.18, 0.65) (Table 3, Appendix B4.1). Between-studies overlaps for the six reviews was 17.65%, CA was 0.21, and CCA was slight at 4.71% (Appendix A11.4). Study-level meta-analysis from 16 unique primary studies found a small non-significant effect size after removing overlapped studies (*g* = 0.39; 95% CI: −0.22, 1.00) (Table 3, Appendix B4.2), which was smaller than the meta-level estimate, with substantial heterogeneity (*I*^2^ = 82.90%). Although there were no significant subgroup differences, only sequential PCDTs had a small significant effect (*g* = 0.28; 95% CI: 0.00, 0.56) within moderator subgroups (Table 3, Appendix B4.3–B4.7, Appendix A12). The 95% prediction interval for meta-level meta-analyses indicated that PCDTs may improve activities of daily living compared to a comparator in future research under comparable conditions, while study-level meta-analyses suggested otherwise.

### Physical—gait outcomes

Meta-level meta-analysis from four reviews on PCDT’s effectiveness on gait found a small significant effect (*g* = 0.27; 95% CI: 0.11, 0.43) (Table 3, Appendix B5.1). Between-studies overlaps for the four reviews was 15.79%, CA was 0.29, and CCA was slight at 5.26% (Appendix A11.5). Study-level meta-analysis from 18 unique primary studies found a moderate non-significant effect size (*g* = 0.55; 95% CI: −0.00, 1.10) (Table 3, Appendix B5.2) which was larger than the meta-level estimate, with substantial heterogeneity (*I*^2^ = 56.40%). Although there were no significant subgroup differences, small significant effects were found within moderator subgroups such as older adults with dementia (*g* = 0.37; 95% CI: 0.07, 0.67), training duration of >12 weeks (*g* = 0.42; 95% CI: 0.20, 0.65), session duration of >60 minutes (*g* = 0.43; 95% CI: 0.25, 0.61), and training frequency of ≤2 times per week (*g* = 0.45; 95% CI: 0.26, 0.64) (Table 3, Appendix B5.3–B5.7, Appendix A12). The 95% prediction interval for both meta-level and study-level meta-analyses indicated that PCDTs may not improve gait compared to a comparator in future research under comparable conditions.

### Physical—balance outcomes

Meta-level meta-analysis from five reviews on PCDT’s effectiveness on balance found a small significant effect (*g* = 0.37; 95% CI: 0.20, 0.55) (Table 3, Appendix B6.1). Between-studies overlaps for the five reviews was 25.00%, CA was 0.34, and CCA was high at 12.50% (Appendix A11.6). Study-level meta-analysis of 16 unique primary studies found a small non-significant effect (*g* = 0.29; 95% CI: −0.11, 0.72) (Table 3, Appendix B6.2) which was smaller than the meta-level estimate, with substantial heterogeneity (*I*^2^ = 67.20%). No significant subgroup differences were found, and within moderator subgroups found no significant effects (Table 3, Appendix B6.3–B6.7, Appendix A12). The 95% prediction interval for meta-level meta-analyses indicated that PCDTs may improve balance compared to a comparator in future research under comparable conditions, while study-level meta-analyses suggested otherwise.

### Publication bias

Evidence of publication bias was only detected in the study-level meta-analyses for activities of daily living (*P* = .03) (Appendix B7, B8). No publication bias was observed for the remaining meta-level and study-level outcomes (*P* = .20–.91).

### GRADE credibility of meta-analyses

Although protective effects were observed across the primary outcomes, all were rated as having low certainty of evidence (Table 4). Risk of bias related to review quality was rated as very serious for all outcomes; risk of bias at the level of included studies was serious for all outcomes. Inconsistency was rated as serious for all outcomes except gait (no serious limitations), and indirectness was rated as very serious for all outcomes except balance (rated as serious).

**Table 4:** GRADE certainty of evidence for meta-level meta-analyses outcomes from included reviews.

Outcome/Design	Number of reviews	Number of participants	Overlapping studies	Criteria Assessment	Direction of effect	Certainty
***Global Cognition***						
SRMA of RCTs	16	12,172	36	Imprecision (No serious limitations); Review risk of bias (Very serious); Included study risk of bias (Serious); Inconsistency (Serious); Indirectness^*^ (Very serious)	Protective	Low certainty
***HRQoL***						
SRMA of RCTs	5	1322	2	Imprecision (No serious limitations); Review risk of bias (Very serious); Included study risk of bias (Serious); Inconsistency (Serious); Indirectness^**^ (Very serious)	Protective	Low certainty
***Physical***						
ADL						
SRMA of RCTs	6	1798	3	Imprecision (No serious limitations); Review risk of bias (Very serious); Included study risk of bias (Serious); Inconsistency (Serious); Indirectness^***^ (Very serious)	Protective	Low certainty
***Gait***						
SRMA of RCTs	4	1386	3	Imprecision (No serious limitations); Review risk of bias (Very serious); Included study risk of bias (Serious); Inconsistency (No serious limitations); Indirectness^****^ (Very serious)	Protective	Low certainty
***Balance***						
SRMA of RCTs	5	1050	4	Imprecision (No serious limitations); Review risk of bias (Very serious); Included study risk of bias (Serious); Inconsistency (Serious); Indirectness^*****^ (Serious)	Protective	Low certainty

*Note.* ADL: Activities of daily living; HRQoL: Health-related quality of life; RCT: Randomised controlled trials; SRMA: Systematic reviews and meta-analysis; ^*^Included a total of 10 studies that did not meet this UR’s eligibility criteria for the meta-level meta-analysis data. Not accounted for GRADE downgrading; ^**^Included a total of 4 studies that did not meet this UR’s eligibility criteria for the meta-level meta-analysis data. Not accounted for GRADE downgrading; ^***^Included a total of 3 studies that did not meet this UR’s eligibility criteria for the meta-level meta-analysis data. Not accounted for GRADE downgrading; ^****^Included a total of 4 studies that did not meet this UR’s eligibility criteria for the meta-level meta-analysis data. Not accounted for GRADE downgrading; ^*****^Included 1 study that did not meet this UR’s eligibility criteria for the meta-level meta-analysis data. Not accounted for GRADE downgrading.

## Discussion

This umbrella review has comprehensively reviewed the effectiveness of PCDT in improving global cognition, health-related quality of life, and physical outcomes of activities of daily living, gait, and balance on older adults with neurocognitive disorders. Meta-level meta-analyses suggested statistically significant improvements for all outcomes except for health-related quality of life, while study-level analyses showed statistically significant effects for global cognition and health-related quality of life. Subgroup analyses suggested that PCDT had greater effects in individuals with mild cognitive impairment compared to those with dementia, and in simultaneous rather than sequential PCDT formats. Higher training frequency was associated with greater effect sizes, whereas longer training or session durations were not. Age showed no significant moderating effect. Most included reviews were of low and very low quality, and meta-level meta-analyses were rated to have low certainty of evidence.

Meta-level and study-level meta-analyses suggested significant effects for global cognition, corroborating findings from previous umbrella reviews that employed qualitative synthesis [15]. Furthermore, improvements in health-related quality of life were suggested to be correlated with improvements in physio-cognitive functions [8, 53], which was consistent with our study-level meta-analytic findings. The inclusion of Li *et al*. [45], may have led to the non-significant meta-level meta-analyses effects due to the inclusion of a large proportion of older adults without any neurocognitive disorders. Otherwise, another possible explanation for health-related quality of life improvements could be that PCDT often involves structured interaction with facilitators or peers, potentially alleviating social isolation in older adults with neurocognitive disorders [54]. Together, these findings support the integration of PCDT into multidisciplinary rehabilitation strategies.

Study-level meta-analyses for physical outcomes suggested non-significant positive effects, contrasting with the significant positive effects found in meta-level meta-analyses. This discrepancy may be explained by a few factors. The effect size from meta-level meta-analysis on activities of daily living may have been overestimated, as it included studies that measured instrumental activities of daily living as activities of daily living despite the temporal precedence in impairment manifestation [55, 56]. Contrastingly, study-level meta-analysis filtered out these studies for a more precise aggregation. For gait and balance, meta-level analyses included studies that grouped these outcomes under broader physical performance metrics, leading to a possible underestimation of the effect size for gait and an overestimation for balance. The substantial heterogeneity in PCDT protocols and the diverse measurement tools used to assess physical outcomes may have also contributed to the non-significant findings in the study-level analyses and limited the generalisability of results, thereby reducing the credibility of the pooled estimates. This unstable consistency of physical outcome findings along with methodological issues highlight the importance for future higher quality reviews to accurately present PCDTs’ effectiveness. Despite PCDT’s key ability to mimic real-world multitasking scenarios, particularly in supporting activities of daily living function [57], while enhancing gait speed and balance [58, 59], the presented findings should be interpreted with caution, taking individual variability and therapeutic goals into account. Until more empirical evidence becomes available, clinicians may consider integrating PCDT with conventional physiotherapy to optimise outcomes [60].

Notably, simultaneous PCDTs appeared to outperform sequential PCDTs, possibly due to greater prefrontal cortex activation [61], which may promote neuroplasticity [62, 63]. Higher weekly training frequencies also seemed more beneficial, consistent with previous studies [64] and the World Health Organisation’s recommendation of 150–300 minutes of physical activity, split into three sessions weekly, for healthy ageing in older adults [65]. Older adults with mild cognitive impairment generally benefited more than those with dementia, aligning with existing theories (Appendix B1) and magnetic resonance imaging studies [66–68]. These findings highlight the potential value of early PCDT implementation and should be considered in clinical decision-making.

In contrast, extended training durations (>12 weeks) or long sessions (>60 minutes) were not advantageous, suggesting that training intensity and variation may be more important for optimising PCDT effectiveness [69, 70]. Age also did not appear to significantly influence PCDT effectiveness, contradicting theories of age-related decline in motor and cognitive function [58] and dual-task inefficiency [71], which are thought to limit PCDT benefits [72]. This is further supported by positive retention effects of working memory and balance even after 12 weeks post completion of simultaneous PCDT, though it should be noted that this was based on a sample of non-cognitively impaired older men and may not accurately translate to cognitive impaired older adults [73]. Hence, although PCDTs may be beneficial across the older adult age spectrum, further research and clinical data are needed to confirm age as a moderator, and future reviews are encouraged to examine PCDT’s long-term retention effects.

The high heterogeneity observed may be explained by the large variability in PCDT protocols, intensity, and compliance rates. While pooling such heterogeneous interventions provides a broad overview, readers should interpret pooled estimates cautiously, as they may obscure meaningful differences between specific PCDT modalities especially across its various clinical applications. Given the mixed significance and high variability in PCDT interventions and moderators, a tailored rather than a standardised approach may be more effective [60]. These findings provide important guidance for refining PCDT interventions and their application in future research and clinical practice. Considering the low and critically low AMSTAR-2 ratings, future reviews will need to be more cautious in their methodology and reporting clarity to improve the overall confidence level of their reported evidence to inform PCDT’s clinical effectiveness.

The following limitations should be considered. This umbrella review only measured global cognition and not the individual cognitive domains, which could have provided specific insights into how PDCT affects cognition. Furthermore, the exclusion of vascular dementia, which commonly co-occurs with Alzheimer’s disease in mixed dementia presentations, may have excluded a clinically significant portion of the patient population who undergo PCDT in real-world rehabilitation settings, thus producing a sample heavily weighted toward the prodromal stages. Additionally, its long-term effectiveness was not assessed, which is important for evaluating its viability for extended use. As most of the reviews were of low and critically low quality, and meta-level meta-analyses had low certainty of evidence, the overall trustworthiness of this umbrella review’s findings could have been undermined. Study-level meta-analyses showed high heterogeneity, which was inevitable due to varying PCDT modalities and measurement tools. Furthermore, quality appraisal was not conducted for individual studies as it was beyond the scope of this umbrella review. Consequently, publication bias may have been compounded from the review level to this umbrella review. Lastly, only reviews published in English were included due to language barriers.

## Conclusion

This umbrella review highlighted the potential of PCDT to improve global cognition, health-related quality of life, activities of daily living, gait, and balance among older adults with neurocognitive disorders, though the heavy weightage toward prodromal stages warrants interpretative caution. While the findings suggest potential benefits, particularly for cognitive outcomes in individuals with MCI, the integration of PCDT into rehabilitation programs should be approached cautiously pending higher-quality evidence. The moderator analyses findings may help guide clinicians in implementing PCDT strategies and optimising its protocols. Importantly, neither age nor longer session durations moderated outcomes, suggesting a broad yet time-efficient applicability. However, substantial heterogeneity, low certainty of evidence, and effectiveness uncertainty from the prediction intervals limit definitive conclusions for both study-level and meta-level findings. Hence, a tailored approach, rather than a uniform application, may be key to optimising effectiveness. High-quality systematic reviews and meta-analyses with standardised interventions, expanded populations, and long-term follow-ups are urgently needed to inform clinical translation.

## Supplementary Material

aa-25-2382-File003_afag061

## Key Points

- PCDT may be more effective than single-task training for older adults with neurocognitive disorders.
- Current evidence is limited by low-quality reviews, requiring higher-quality research.
- Clinicians should tailor PCDT, as patients with dementia may benefit less than those with MCI.
