# Supplementary material for: Effectiveness of physio-cognitive dual-task training on improving global cognition, health-related quality of life, and physical outcomes among older adults with neurocognitive disorders: an umbrella review
Source: Age Ageing. 2026 Mar 27;55(3):afag061. doi: 10.1093/ageing/afag061 (PMC13023371; doi:10.1093/ageing/afag061)

**Supplementary Materials for: Effectiveness of physio-cognitive dual-task training on improving global cognition, health-related quality-of-life, and physical outcomes among older adults with neurocognitive disorders: An umbrella review**

**Table of Contents**

[Appendix A1. PRIO-harms Checklist of Items to Include when Reporting an Overview of Systematic Reviews (OoSRs) 6](#_Toc222917627)

[Appendix A2. Preferred Reporting Items for Systematic Reviews and Meta-Analyses 9](#_Toc222917628)

[Appendix A3. Eligibility Criteria for the Umbrella Review 12](#_Toc222917629)

[Appendix A4. Search Strategy 13](#_Toc222917630)

[Appendix A5. Data Extraction Form 17](#_Toc222917631)

[Appendix A6. Critical Appraisal for Systematic Reviews Using AMSTAR-2 18](#_Toc222917632)

[Appendix A7.1. Algorithm for ‘‘Downgrades’’ to Levels of Evidence in Reviews 19](#_Toc222917633)

[Appendix A7.2. Formula for GRADE Level of Evidence from Number of Downgrades Determined Using the Algorithm 19](#_Toc222917634)

[Appendix A8. Lists of Excluded Reviews with Exclusion Reasons 20](#_Toc222917635)

[Appendix A9. Lists of Excluded Primary Studies from Corresponding Study-Level Meta-Analyses with Exclusion Reasons 22](#_Toc222917636)

[Appendix A10. Summary of Affiliation, Funding, Publisher, Protocol Registration of Included Reviews 23](#_Toc222917637)

[Appendix A11.1. Between-Studies Overlaps for Meta-Analyses Measuring Outcomes Relevant to this Umbrella Review 24](#_Toc222917638)

[Appendix A11.2. Within-Studies Overlaps for Global Cognitive Outcomes Using PCDT 26](#_Toc222917639)

[Appendix A11.3. Within-Studies Overlaps for HRQoL Outcomes Using PCDT 28](#_Toc222917640)

[Appendix A11.4. Within-Studies Overlaps for ADL Outcomes Using PCDT 29](#_Toc222917641)

[Appendix A11.5. Within-Studies Overlaps for Gait Outcomes Using PCDT 30](#_Toc222917642)

[Appendix A11.6. Within-Studies Overlaps for Balance Outcomes Using PCDT 31](#_Toc222917643)

[Appendix A12. Mixed-Effects Meta-Regression of Age on Outcomes 32](#_Toc222917644)

[Appendix B1. Cognitive Decline Trajectory from Mild Cognitive Impairment to Dementia 33](#_Toc222917645)

[Appendix B2.1. Forest Plot of Effect Sizes (Hedges’ g) of Meta-Level Data for Global Cognition 34](#_Toc222917646)

[Appendix B2.2. Forest Plot of Effect Sizes (Hedges’ g) of Study-Level Data for Global Cognition 35](#_Toc222917647)

[Appendix B2.3. Subgroup Analyses of Forest Plot of Effect Sizes (Hedges’ g) of Study-Level Data for NCD Nature for Global Cognition 36](#_Toc222917648)

[Appendix B2.4. Subgroup Analyses of Forest Plot of Effect Sizes (Hedges’ g) of Study-Level Data for Intervention Type for Global Cognition 37](#_Toc222917649)

[Appendix B2.5. Subgroup Analyses of Forest Plot of Effect Sizes (Hedges’ g) of Study-Level Data for Training Duration for Global Cognition 38](#_Toc222917650)

[Appendix B2.6. Subgroup Analyses of Forest Plot of Effect Sizes (Hedges’ g) of Study-Level Data for Session Duration for Global Cognition 39](#_Toc222917651)

[Appendix B2.7. Subgroup Analyses of Forest Plot of Effect Sizes (Hedges’ g) of Study-Level Data for Training Frequency for Global Cognition 40](#_Toc222917652)

[Appendix B3.1. Forest Plot of Effect Sizes (Hedges’ g) of Meta-Level Data for HRQoL 41](#_Toc222917653)

[Appendix B3.2. Forest Plot of Effect Sizes (Hedges’ g) of Study-Level Data for HRQoL 42](#_Toc222917654)

[Appendix B3.3. Subgroup Analyses of Forest Plot of Effect Sizes (Hedges’ g) of Study-Level Data for NCD Type for HRQoL 43](#_Toc222917655)

[Appendix B3.4. Subgroup Analyses of Forest Plot of Effect Sizes (Hedges’ g) of Study-Level Data for Intervention Type for HRQoL 44](#_Toc222917656)

[Appendix B3.5. Subgroup Analyses of Forest Plot of Effect Sizes (Hedges’ g) of Study-Level Data for Training Duration for HRQoL 45](#_Toc222917657)

[Appendix B3.6. Subgroup Analyses of Forest Plot of Effect Sizes (Hedges’ g) of Study-Level Data for Session Duration for HRQoL 46](#_Toc222917658)

[Appendix B3.7. Subgroup Analyses of Forest Plot of Effect Sizes (Hedges’ g) of Study-Level Data for Training Frequency for HRQoL 47](#_Toc222917659)

[Appendix B4.1. Forest Plot of Effect Sizes (Hedges’ g) of Meta-Level Data for ADL 48](#_Toc222917660)

[Appendix B4.2. Forest Plot of Effect Sizes (Hedges’ g) of Study-Level Data for ADL 49](#_Toc222917661)

[Appendix B4.3. Subgroup Analyses of Forest Plot of Effect Sizes (Hedges’ g) of Study-Level Data for NCD Nature for ADL 50](#_Toc222917662)

[Appendix B4.4. Subgroup Analyses of Forest Plot of Effect Sizes (Hedges’ g) of Study-Level Data for Intervention Type for ADL 51](#_Toc222917663)

[Appendix B4.5. Subgroup Analyses of Forest Plot of Effect Sizes (Hedges’ g) of Study-Level Data for Training Duration for ADL 52](#_Toc222917664)

[Appendix B4.6. Subgroup Analyses of Forest Plot of Effect Sizes (Hedges’ g) of Study-Level Data for Session Duration for ADL 53](#_Toc222917665)

[Appendix B4.7. Subgroup Analyses of Forest Plot of Effect Sizes (Hedges’ g) of Study-Level Data for Training Frequency for ADL 54](#_Toc222917666)

[Appendix B5.1. Forest Plot of Effect Sizes (Hedges’ g) of Meta-Level Data for Gait 55](#_Toc222917667)

[Appendix B5.2. Forest Plot of Effect Sizes (Hedges’ g) of Study-Level Data for Gait 56](#_Toc222917668)

[Appendix B5.3. Subgroup Analyses of Forest Plot of Effect Sizes (Hedges’ g) of Study-Level Data for NCD Nature for Gait 57](#_Toc222917669)

[Appendix B5.4. Subgroup Analyses of Forest Plot of Effect Sizes (Hedges’ g) of Study-Level Data for Intervention Type for Gait 58](#_Toc222917670)

[Appendix B5.5. Subgroup Analyses of Forest Plot of Effect Sizes (Hedges’ g) of Study-Level Data for Training Duration for Gait 59](#_Toc222917671)

[Appendix B5.6. Subgroup Analyses of Forest Plot of Effect Sizes (Hedges’ g) of Study-Level Data for Session Duration for Gait 60](#_Toc222917672)

[Appendix B5.7. Subgroup Analyses of Forest Plot of Effect Sizes (Hedges’ g) of Study-Level Data for Training Frequency for Gait 61](#_Toc222917673)

[Appendix B6.1. Forest Plot of Effect Sizes (Hedges’ g) of Meta-Level Data for Balance 62](#_Toc222917674)

[Appendix B6.2. Forest Plot of Effect Sizes (Hedges’ g) of Study-Level Data for Balance 63](#_Toc222917675)

[Appendix B6.3. Subgroup Analyses of Forest Plot of Effect Sizes (Hedges’ g) of Study-Level Data for NCD Nature for Balance 64](#_Toc222917676)

[Appendix B6.4. Subgroup Analyses of Forest Plot of Effect Sizes (Hedges’ g) of Study-Level Data for Intervention Type for Balance 65](#_Toc222917677)

[Appendix B6.5. Subgroup Analyses of Forest Plot of Effect Sizes (Hedges’ g) of Study-Level Data for Training Duration for Balance 66](#_Toc222917678)

[Appendix B6.6. Subgroup Analyses of Forest Plot of Effect Sizes (Hedges’ g) of Study-Level Data for Session Duration for Balance 67](#_Toc222917679)

[Appendix B6.7. Subgroup Analyses of Forest Plot of Effect Sizes (Hedges’ g) of Study-Level Data for Training Frequency for Balance 68](#_Toc222917680)

[Appendix B7. Funnel Plots and Publication Bias of Meta-Level Data Outcomes 69](#_Toc222917681)

[Appendix B8. Funnel Plots and Publication Bias of Study-Level Data Outcomes 70](#_Toc222917682)

# **Appendix A1.** PRIO-harms Checklist of Items to Include when Reporting an Overview of Systematic Reviews (OoSRs)

| **Section/topic** | **(Sub-)item#** | **Checklist item** | **Reported on page#** |
| --- | --- | --- | --- |
| **Title**  1. Title | 1a | Specify the study design with terms such as ‘‘overview of (systematic) reviews,’’ ‘‘umbrella review,’’ ‘‘(systematic) review of systematic reviews,’’ or ‘‘(systematic) meta-review’’ in the title of the OoSRs. | 1 |
|  | 1b | Mention ‘‘safety’’ or harms related terms, or the adverse event(s) of interest in the title of the OoSRs. | - |
| **Abstract**  2. Structured-like summary | 2a | Provide a structured-like abstract, as applicable: background, objective, data sources, selection criteria, data extraction, review appraisal, data synthesis methods, results, limitations, conclusions. | 1 |
|  | 2b | Report the main findings of analysis of harms undertaken in the OoSRs or/and in the included SRs. | 1 |
| **Introduction**  3.Rationale | 3a | Specify the rationale and the scope (wide or narrow agendas) for the overview in the context of an existing body of knowledge on the topic. | 3 |
|  | 3b | Provide a balanced presentation of potential benefits and harms of the intervention(s). | 3 |
|  | 3c^a^ | Define which events are considered harms according to previous literature and provide a clear rationale for the specific harms included in the OoSRs. | - |
| 4. Objectives (PICOS) | 4 | Provide an explicit statement of research question(s) that specifies PICOS: | 3 |
| **Methods** |  | Participants Interventions Comparators Outcomes Study design | 3-5 |
| 5. Protocol and registration | 5a | Indicate if a protocol exists or not. | 3 |
|  | 5b | If registered, provide the name of the registry (such as a valid Web address, PROSPERO). | 3 |
| 6. Eligibility criteria and outcomes of interest | 6a | Specify inclusion and exclusion criteria for study design, participants, interventions, and comparators in detail. | 3-4 |
|  | 6b | List (and define whenever it is necessary) the outcomes for which data were recorded, ideally include prioritization of main and additional outcomes. | 4 |
|  | 6c | Include adverse events as (primary or secondary) outcome of interest. Define them and grade their severity (such as mild, moderate, severe, fatal; severity could also be described in the appendix), if appropriate. | - |
|  | 6d^b^ | Specify report characteristics (such as language restrictions, publication status, and years considered) used as criteria for eligibility for the OoSRs (see also item 7). | 3-4 |
| 7. Information sources | 7a | Search at least two electronic databases. | 4 |
|  | 7b | Search supplementary sources (e.g., hand searching, reference lists, related reviews and guidelines, protocol registries, conference abstracts, and other gray literature). | 4 |
|  | 7c | Report the date of last search and/or dates of coverage for each database. | 4 |
| 8. Search strategy^c^ | 8a | Specify full electronic search strategy (algorithm) for at least one database including any limits used (e.g., language and date restrictions (see also subitems 6d and 7c) such that it could be repeated. | 3-4,  Supplementary material Table A4 |
|  | 8b | Present any additional search process (e.g., algorithm or filter for adverse events, searches in pertinent websites) specifically to identify adverse events that have been investigated. | - |
| 9. Data management and selection process | 9a^d^ | Describe the software that was used to manage records and data throughout the OoSRs. | 4 |
|  | 9b | Define what is an SR and provide the process for selecting SRs and its relevant details (screening the title and abstract or full text by at least two reviewers, selection by multiple independent investigators and resolving disagreements by consensus). | 4 |
|  | 9c | Report any attempt to handle overlapping (include one review among multiple potential candidates by choosing for example the most updated SR, the most methodologically rigorous SR or the SR with larger number of primary studies). | 4-5 |
| 10. Additional search for primary studies | 10 | Report additional search to identify eligible primary studies (e.g., searching in more databases or update the search) and its relevant details. | - |
| 11. Data collection process | 11a | Describe the method of data extraction from included SRs (e.g., data collection form, extraction in duplicate and independently, resolving disagreements by consensus). | 4,  Supplementary material Table A3 |
|  | 11b | Report any processes for obtaining, confirming, or updating data from investigators (e.g., contact with authors of included reviews, obtain data from primary studies of included reviews). | 4 |
| 12. Data items | 12 | List (and define whenever is necessary) the variables for which data were recorded (e.g., PICOS items, number of included studies and participants, dose, length of follow up, results, funding sources) and any data assumptions and simplifications made. | 4 |
| 13. Assessment of methodological quality and quality of evidence | 13a | State the evaluation of reporting or/and methodological quality (e.g., using PRISMA or PRISMA-harms, AMSTAR or R-AMSTAR) of the included reviews. | 4 |
|  | 13b^e^ | State the evaluation of quality for individual studies that are included in the SRs (inform whether tools such as Jadad or RoB of Cochrane were used by the included reviews) and for the additional primary studies. | 5, Table 2 |
|  | 13c | State the evaluation of quality of evidence (e.g., using GRADE approach). | 5 |
|  | 13d | Describe the methods (e.g., piloted forms, independently, in duplicate) used for the quality assessment. | 4-5 |
| 14. Meta-bias(es) | 14 | Specify any planned assessment of meta-bias(es) (such as publication bias or selective reporting across studies, ROBIS tool). | 5 |
| 15. Data synthesis | 15a | Specify clearly the method (narrative, meta-analysis, or network metaanalysis) of handling or synthesizing data and their details (e.g., state the principal summary measures that were extracted or calculated, how heterogeneity was assessed, what statistical approaches were used if a quantitative synthesis has been conducted) | 5 |
|  | 15b | Describe the software that was used to analyze the data if a quantitative synthesis has been conducted. | 5 |
|  | 15c | Report if zero events are included in the studies and how they were handled in statistical analyses, if relevant. | 4 |
|  | 15d | Describe methods of any prespecified additional analyses (such as sensitivity or subgroup analyses, meta-regression). | 5 |
| **Results**  16. Review and primary study selection | 16a | Provide the details of review selection (e.g., numbers of reviews screened, retrieved, and included and excluded in the overview) and the number of the additional eligible primary studies that were included, ideally with a flow diagram of the overview process. | 5, Figure 1 |
|  | 16b | Present a flow diagram that gives separately the number of studies focused on harms outcomes. | - |
|  | 16c^c^ | List the studies (full citation) that were excluded after reading the full text and provide reasons. | Supplementary material Table A8 |
| 17. Review and primary study characteristics | 17a^c^ | Describe characteristics of each included SR in tables (such as title or author, search date, PICOS, design and number of studies included, number and age range of participants, dose/frequency, follow up period [treatment duration], review limitations, results or conclusion) and of each additional primary study. | 5, Table 1 and Table 2 |
|  | 17b | For each included SR report language and publication status restrictions that have been used. | 5, Table 2 |
| 18. Overlapping | 18 | Present or/and discuss about overlapping of studies within SRs (at least one of the following): | 6-7 |
| 19. Present assessment of methodological quality and quality of evidence | 19 | Present measures of overlap (such as CCA).  Provide citation matrix.^c^  Give the number of index publications or/and discuss about overlapping.^f^  Present results in text or/and tables^c^ of any quality assessment (see also subitems 13aec):  Reporting or/and methodological quality of the included SRs.  Inform for the quality of the individual studies that were included in the SRs (report results for sequence generation, allocation concealment, blinding, withdrawals, bias etc.) and for the additional included primary studies. | 6-7,  Supplementary material Table A11.1-11.6 |
| 20. Present meta-bias(es) | 20 | Quality of evidence.  Present results of any assessment of meta-bias(es) (such as publication bias or selective reporting across studies, ROBIS assessment). | 7, Supplementary material Figure A7, A8 |
| 21. Synthesis of results | 21a | Summarize and present the main findings of the overview for benefits and harms. If a quantitative synthesis has been conducted, present each summary measure with a confidence interval, prediction interval, or a credible interval and measures of heterogeneity or inconsistency. | 6-7, Table 3 |
|  | 21b | Give results of any additional analyses, if done (such as sensitivity, subgroup analyses, or meta-regression). | 6-7 |
|  | 21c | Report results for adverse events separately for each intervention. | - |
| **Discussion**  22. Summary of evidence | 22 | Provide a concise summary of the main findings with the strengths and shortcomings of evidence for each main outcome. | 7-9 |
| 23. Limitations | 23a | Discuss limitations of either the overview or included studies (or both) (e.g., different eligibility criteria, limitations of searching reviews, language restrictions, publication and selection bias). | 8 |
|  | 23b | Report possible limitations of the included reviews related to harms (issues of missing data and information, definitions of harms, rare adverse effects). | - |
| 24. Conclusions | 24a | Provide a general interpretation of the results in coherence with the review findings and present implications for practice; consider the harms equally as carefully as the benefits and in the context of other evidence. | 9 |
|  | 24b | Present implications for future research. | 8-9 |
| **Authorship**  25. Contributions of authors | 25 | Provide contributions of authors. | 10 |
| 26. Dual (co-)authorship | 26 | Report about dual (co-)authorship in the limitation or declarations of interest section. | - |
| **Funding**  27. Funding or other support | 27a | Indicate sources of financial and other support for the OoSRs (direct funding) or for the authors (indirect funding), or report no funding. | 10 |
|  | 27b | Provide name for the overview funder and/or sponsor, or for the authors’ supporters. | - |
|  | 27c | Describe roles of funder(s), sponsor(s), and/or institution(s), if any, in conducted the OoSRs. | - |

Abbreviations: SRs, systematic reviews; PICOS, participants, interventions, comparisons, outcomes, and study design; CCA, corrected covered area; PRIO-harms, Preferred Reporting Items for OoSRs.

a Applicable mainly for OoSRs that focus on adverse events. The description could be placed in methods section.

b Language restrictions, publication status, and years could also be reported in information sources topic - see item 7.

c It could also be placed in an appendix as a supplementary material.

d The software used for the management of the records and data could be placed in the data collection process - see item 11.

e The way of evaluation (e.g., instruments) can be reported in item 19.

^f^ Index publication is the first occurrence of a primary publication in the included reviews. Discussion for overlapping might be placed in the discussion section.

Modified and extended for overviews of systematic reviews (OoSRs) from: Moher D, Liberati A, Tetzlaff J, Altman DG, The PRISMA Group (2009). Preferred Reporting Items for Systematic Reviews and Meta-Analyses: The PRISMA Statement. PLoS Med 6(7): e1000097. https://doi.org/10.1371/journal.pmed.1000097

# **Appendix A2.** Preferred Reporting Items for Systematic Reviews and Meta-Analyses

| **Section and Topic** | **Item #** | **Checklist item** | **Location where item is reported** |
| --- | --- | --- | --- |
| **TITLE** | | |  |
| Title | 1 | Identify the report as a systematic review. | 1 |
| **ABSTRACT** | | |  |
| Abstract | 2 | See the PRISMA 2020 for Abstracts checklist. | 1 |
| **INTRODUCTION** | | |  |
| Rationale | 3 | Describe the rationale for the review in the context of existing knowledge. | 3 |
| Objectives | 4 | Provide an explicit statement of the objective(s) or question(s) the review addresses. | 3 |
| **METHODS** | | |  |
| Eligibility criteria | 5 | Specify the inclusion and exclusion criteria for the review and how studies were grouped for the syntheses. | 3-4 |
| Information sources | 6 | Specify all databases, registers, websites, organisations, reference lists and other sources searched or consulted to identify studies. Specify the date when each source was last searched or consulted. | 4 |
| Search strategy | 7 | Present the full search strategies for all databases, registers and websites, including any filters and limits used. | 3-4, Supplementary material Table A4 |
| Selection process | 8 | Specify the methods used to decide whether a study met the inclusion criteria of the review, including how many reviewers screened each record and each report retrieved, whether they worked independently, and if applicable, details of automation tools used in the process. | 3-4 |
| Data collection process | 9 | Specify the methods used to collect data from reports, including how many reviewers collected data from each report, whether they worked independently, any processes for obtaining or confirming data from study investigators, and if applicable, details of automation tools used in the process. | 4, Supplementary material Table A3 |
| Data items | 10a | List and define all outcomes for which data were sought. Specify whether all results that were compatible with each outcome domain in each study were sought (e.g. for all measures, time points, analyses), and if not, the methods used to decide which results to collect. | 4, Supplementary material Table A3 |
|  | 10b | List and define all other variables for which data were sought (e.g. participant and intervention characteristics, funding sources). Describe any assumptions made about any missing or unclear information. | 4 |
| Study risk of bias assessment | 11 | Specify the methods used to assess risk of bias in the included studies, including details of the tool(s) used, how many reviewers assessed each study and whether they worked independently, and if applicable, details of automation tools used in the process. | 5 |
| Effect measures | 12 | Specify for each outcome the effect measure(s) (e.g. risk ratio, mean difference) used in the synthesis or presentation of results. | 5 |
| Synthesis methods | 13a | Describe the processes used to decide which studies were eligible for each synthesis (e.g. tabulating the study intervention characteristics and comparing against the planned groups for each synthesis (item #5)). | 3-4 |
|  | 13b | Describe any methods required to prepare the data for presentation or synthesis, such as handling of missing summary statistics, or data conversions. | 4-5 |
|  | 13c | Describe any methods used to tabulate or visually display results of individual studies and syntheses. | 5 |
|  | 13d | Describe any methods used to synthesize results and provide a rationale for the choice(s). If meta-analysis was performed, describe the model(s), method(s) to identify the presence and extent of statistical heterogeneity, and software package(s) used. | 5 |
|  | 13e | Describe any methods used to explore possible causes of heterogeneity among study results (e.g. subgroup analysis, meta-regression). | 5 |
|  | 13f | Describe any sensitivity analyses conducted to assess robustness of the synthesized results. | 5 |
| Reporting bias assessment | 14 | Describe any methods used to assess risk of bias due to missing results in a synthesis (arising from reporting biases). | 5 |
| Certainty assessment | 15 | Describe any methods used to assess certainty (or confidence) in the body of evidence for an outcome. | 5 |
| **RESULTS** | | |  |
| Study selection | 16a | Describe the results of the search and selection process, from the number of records identified in the search to the number of studies included in the review, ideally using a flow diagram. | 5, Figure 1 |
|  | 16b | Cite studies that might appear to meet the inclusion criteria, but which were excluded, and explain why they were excluded. | 5, Supplementary material Table A8 |
| Study characteristics | 17 | Cite each included study and present its characteristics. | 5, Table 1, Table 2 |
| Risk of bias in studies | 18 | Present assessments of risk of bias for each included study. | 5-6, Table 2, |
| Results of individual studies | 19 | For all outcomes, present, for each study: (a) summary statistics for each group (where appropriate) and (b) an effect estimate and its precision (e.g. confidence/credible interval), ideally using structured tables or plots. | 5, Table 1 |
| Results of syntheses | 20a | For each synthesis, briefly summarise the characteristics and risk of bias among contributing studies. | 7 |
|  | 20b | Present results of all statistical syntheses conducted. If meta-analysis was done, present for each the summary estimate and its precision (e.g. confidence/credible interval) and measures of statistical heterogeneity. If comparing groups, describe the direction of the effect. | 6-7, Table 3 |
|  | 20c | Present results of all investigations of possible causes of heterogeneity among study results. | 6-7, Table 3 |
|  | 20d | Present results of all sensitivity analyses conducted to assess the robustness of the synthesized results. | 6-7. Table 3 |
| Reporting biases | 21 | Present assessments of risk of bias due to missing results (arising from reporting biases) for each synthesis assessed. | 7 |
| Certainty of evidence | 22 | Present assessments of certainty (or confidence) in the body of evidence for each outcome assessed. | 7, Table 4 |
| **DISCUSSION** | | |  |
| Discussion | 23a | Provide a general interpretation of the results in the context of other evidence. | 7-9 |
|  | 23b | Discuss any limitations of the evidence included in the review. | 8 |
|  | 23c | Discuss any limitations of the review processes used. | 8 |
|  | 23d | Discuss implications of the results for practice, policy, and future research. | 8-9 |
| **OTHER INFORMATION** | | |  |
| Registration and protocol | 24a | Provide registration information for the review, including register name and registration number, or state that the review was not registered. | 3 |
|  | 24b | Indicate where the review protocol can be accessed, or state that a protocol was not prepared. | 3 |
|  | 24c | Describe and explain any amendments to information provided at registration or in the protocol. | - |
| Support | 25 | Describe sources of financial or non-financial support for the review, and the role of the funders or sponsors in the review. | 10 |
| Competing interests | 26 | Declare any competing interests of review authors. | 10 |
| Availability of data, code and other materials | 27 | Report which of the following are publicly available and where they can be found: template data collection forms; data extracted from included studies; data used for all analyses; analytic code; any other materials used in the review. | Supplementary material Tables and Figures. |

*From:*  Page MJ, McKenzie JE, Bossuyt PM, Boutron I, Hoffmann TC, Mulrow CD, et al. The PRISMA 2020 statement: an updated guideline for reporting systematic reviews. BMJ 2021;372:n71. doi: 10.1136/bmj.n71. This work is licensed under CC BY 4.0. To view a copy of this license, visit <https://creativecommons.org/licenses/by/4.0/>

# **Appendix A3.** Eligibility Criteria for the Umbrella Review

| Criteria | Inclusion Criteria | Exclusion Criteria |
| --- | --- | --- |
| Population | Participants with a mean age of at least 50 years old, regardless of gender, ethnicity, and socioeconomic status in the community or care facility setting; diagnosed or clinically evaluated to have age-related neurocognitive disorders of mild cognitive impairment and dementias of similar aetiologies (Chen et al., 2023; O'Hara et al., 2022). | Reviews with less than 50% of older adults diagnosed with neurocognitive disorders meeting the inclusion list; Neurocognitive disorders derived from the following: Vascular dementia as it is derived from different aetiologies (Sanders et al., 2023), traumatic brain injuries, brain lesions or masses, infectious diseases (neurocognitive disorders resulting from infections such as HIV/AIDS, encephalitis or meningitis), metabolic or endocrine systemic, genetic or psychiatric disorders, substance use, and external environment (environmental toxins, radiation exposure etc.), as they are all of different aetiologies (Dhakal & Bobrin, 2023). |
| Intervention | Physio-cognitive dual-task training combining a physical intervention with a cognitive intervention, either simultaneous or sequential or in nature. | Interventions that were not physio-cognitive dual-task or had unclear interventions. |
| Comparator/ Control | Active controls (physical, cognitive, or sham interventions in nature), passive controls (treatment as usual, no intervention, wait-list), or a combination of both. |  |
| Outcomes | Reported one or more of the following outcomes: Global cognition, Physical function (Activities of daily living, Gait, Balance), Health-related quality of life. |  |
| Review Typology | Systematic reviews with meta-analyses and meta-analyses. | Incomplete reviews, conference papers, book chapters, letters, and editorials. |
| Publication Year | From inception to 31^st^ December 2024. |  |
| Publication Language | English. | Reviews with no English translated publications. |

# **Appendix A4.** Search Strategy

| Database | Index and Keyword Terms | |
| --- | --- | --- |
| Pubmed  (Medline)  Filter English Language, Inception to 31 Dec 2024: 946 results | 1 | ("Aged"[Mesh]) OR (Old[tiab] OR Elder*[tiab] OR Aging[tiab] OR Aged[tiab] OR “Older Adults”[tiab] OR “Older-adults”[tiab]) |
|  | 2 | ("Dementia"[Mesh] OR "Cognitive Dysfunction"[Mesh]) OR ((Cogniti*[tiab] AND (Impair*[tiab] OR Decline[tiab] OR Dysfunction[tiab] OR Disorder[tiab] OR Problem[tiab] OR Issue[tiab])) OR Alzheimer*[tiab] OR Dementia[tiab]) |
|  | 3 | (“Cognitive Behavioral Therapy”[MESH]) OR (“Cognitive Behavioral Therapy”[tiab] OR “Cognitive Behavioural Therapy”[tiab] OR CBT[tiab] OR “Cognitive Training”[tiab] OR “Cognitive Therapy”[tiab] OR “Cognitive Exercise*”[tiab] OR “Cognitive Rehabilitation”[tiab] OR “Cognitive Intervention”[tiab] OR “Cognitive Stimulation”[tiab] OR “Cognitive Method”[tiab] OR “Cognitive Program*”[tiab] OR “Cognitive Treatment”[tiab]) |
|  | 4 | (“Exercise”[MESH]) OR (“Physical Training”[tiab] OR “Physical Therapy”[tiab] OR “Physical Exercise*”[tiab] OR “Physical Rehabilitation”[tiab] OR “Physical Activit*”[tiab] OR “Motor Training”[tiab] OR “Motor Therapy”[tiab] OR “Motor Exercise*”[tiab] OR “Motor Stimulation”[tiab] OR “Motor Activit*”[tiab] OR “Aerobic Training*”[tiab] OR “Aerobic Therapy*”[tiab] OR “Aerobic Exercise*”[tiab] OR “Aerobic Rehabilitation”[tiab] OR “Aerobic Activit*”[tiab] OR “Strength Training”[tiab] OR “Strength Exercise*”[tiab] OR “Exercise”[tiab]) |
|  | 5 | (Multi-component[tiab] OR Multicomponent[tiab] OR Multidomain[tiab] OR Multi-domain[tiab] OR Multimodal[tiab] OR Multi-modal[tiab] OR Combin*[tiab] OR Dual-Task[tiab] OR Dualtask[tiab] OR “Combined Modality Therapy”[tiab] OR Simultaneous[tiab] OR Sequential[tiab] OR Synergistic[tiab] OR Exergam*[tiab]) |
|  | 6 | ((“Cognitive Motor”[tiab] OR “Cognitive-Motor”[tiab] OR “Motor-Cognitive”[tiab] OR “Motor-cognitive”[tiab]) AND (Therapy[tiab] OR Intervention[tiab] OR “Exercise*”[tiab] OR Rehabilitation[tiab])) OR ((“Non-pharmacological”[tiab] AND (Cogniti*[tiab]) AND (Physical[tiab] OR Motor[tiab] OR Aerobic[tiab] OR Strength[tiab]))) AND (Training[tiab] OR Exercise[tiab] OR Therapy[tiab] OR Treatment[tiab] OR Intervention[tiab]) OR Exergam*[tiab] |
|  | 7 | #1 AND #2 AND (((#3 AND #4) AND #5) OR #6) |
| Cochrane Library  Filter English Language, Inception to 31 Dec 2024: 635 results | 1 | MeSH descriptor: [Aged] explode all trees  (Old* OR Elder* OR Aging OR Aged OR “Older Adults” OR “Older-adults”):ti,ab,kw |
|  | 2 | MeSH descriptor: [Dementia] explode all trees  MeSH descriptor: [Cognitive Dysfunction] explode all trees  (((Cogniti*) NEAR/4 (Impair* OR Decline OR Dysfunction OR Disorder OR Problem OR Issue)) OR Alzheimer* OR Dementia):ti,ab,kw |
|  | 3 | MeSH descriptor: [Cognitive Behavioral Therapy] explode all trees  (“Cognitive Behavio#ral Therapy” OR CBT OR “Cognitive Training” OR “Cognitive Therapy” OR “Cognitive Exercise*” OR “Cognitive Rehabilitation” OR “Cognitive Intervention” OR “Cognitive Stimulation” OR “Cognitive Method” OR “Cognitive Program*” OR “Cognitive Treatment”):ti,ab,kw |
|  | 4 | MeSH descriptor: [Exercise] explode all trees  (“Physical Training” OR “Physical Therapy” OR “Physical Exercise*” OR “Physical Rehabilitation” OR “Physical Activit*” OR “Motor Training” OR “Motor Therapy” OR “Motor Exercise*” OR “Motor Stimulation” OR “Motor Activit*” OR “Aerobic Training*” OR “Aerobic Therapy*” OR “Aerobic Exercise*” OR “Aerobic Rehabilitation” OR “Aerobic Activit*” OR “Strength Training” OR “Strength Exercise*” OR “Exercise”):ti,ab,kw |
|  | 5 | (Multi-component OR Multicomponent OR Multidomain OR Multi-domain OR Multimodal OR Multi-modal OR Combin* OR Dual-Task OR Dualtask OR “Combined Modality Therapy” OR Simultaneous OR Sequential OR Synergistic OR Exergam*):ti,ab,kw |
|  | 6 | (((“Cognitive Motor” OR “Cognitive-Motor” OR “Motor-Cognitive” OR “Motor-cognitive”) AND (Therapy OR Intervention OR “Exercise*” OR Rehabilitation)) OR ((“Non-pharmacological” AND (Cogniti*) AND (Physical OR Motor OR Aerobic OR Strength))) AND (Training OR Exercise OR Therapy OR Treatment OR Intervention) OR Exergam*):ti,ab,kw |
|  | 7 | #1 AND #2 AND (((#3 AND #4) AND #5) OR #6) |
| Cinahl Complete  (EBSCOhost)  Filter English Language, Inception to 31 Dec 2024: 448 results | 1 | (MH “Aged+”) OR Old* OR Elder* OR Aging OR Aged OR “Older Adults” OR “Older-adults” |
|  | 2 | (MM “Dementia+”) OR (MM “Mild Cognitive Impairment) OR (Cogniti* AND (Impair* OR Decline OR Dysfunction OR Disorder OR Problem OR Issue) OR Alzheimer* OR Dementia) |
|  | 3 | (MM "Rehabilitation, Cognitive") OR (MH "Cognitive Therapy+") OR (“Cognitive Behavio#ral therapy” OR CBT OR “Cognitive Training” OR “Cognitive Therapy” OR “Cognitive Exercise*” OR “Cognitive Rehabilitation” OR “Cognitive Intervention” OR “Cognitive Stimulation” OR “Cognitive Method” OR “Cognitive Program*” OR “Cognitive Treatment”) |
|  | 4 | (MM “Exercise+”) OR (“Physical Training”OR “Physical Therapy” OR “Physical Exercise*” OR “Physical Rehabilitation” OR “Physical Activit*” OR “Motor Training” OR “Motor Therapy” OR “Motor Exercise*” OR “Motor Stimulation” OR “Motor Activit*” OR “Aerobic Training*” OR “Aerobic Therapy*” OR “Aerobic Exercise*” OR “Aerobic Rehabilitation” OR “Aerobic Activit*” OR “Strength Training” OR “Strength Exercise*” OR “Exercise”) |
|  | 5 | (Multi-component OR Multicomponent OR Multidomain OR Multi-domain OR Multimodal OR Multi-modal OR Combin* OR Dual-Task OR Dualtask OR “Combined Modality Therapy” OR Simultaneous OR Sequential OR Synergistic OR Exergam*) |
|  | 6 | ((“Cognitive Motor” OR “Cognitive-Motor” OR “Motor-Cognitive” OR “Motor-cognitive”) AND (Therapy OR Intervention OR “Exercise*” OR Rehabilitation)) OR ((“Non-pharmacological” AND (Cogniti*) AND (Physical OR Motor OR Aerobic OR Strength))) AND (Training OR Exercise OR Therapy OR Treatment OR Intervention) OR Exergam* |
|  | 7 | TI (S1 AND S2 AND (((S3 AND S4) AND S5) OR S6)) OR AB (S1 AND S2 AND (((S3 AND S4) AND S5) OR S6)) |
| Embase  (Elsevier)  Filter English Language, Inception to 31 Dec 2024: 1435 results | 1 | (‘aged’/exp OR (Old OR Elder* OR Aging OR Aged OR “Older Adults” OR “Older-adults”):ti,ab,kw) |
|  | 2 | (‘cognitive defect’/exp OR (Cogniti* AND (Impair* OR Decline OR Dysfunction OR Disorder OR Problem OR Issue) OR Alzheimer* OR Dementia OR "Cognitive Dysfunction"):ti,ab,kw) |
|  | 3 | (‘cognitive rehabilitation’/exp OR ‘cognitive behavioral therapy’/exp OR (“Cognitive Behavioral Therapy” OR “Cognitive Behavioural Therapy” OR CBT OR “Cognitive Training” OR “Cognitive Therapy” OR “Cognitive Exercise*” OR “Cognitive Intervention” OR “Cognitive Stimulation” OR “Cognitive Method” OR “Cognitive Program*” OR “Cognitive Treatment”):ti,ab,kw) |
|  | 4 | (‘exercise’/exp OR (“Physical Training” OR “Physical Therapy” OR “Physical Exercise*” OR “Physical Rehabilitation” OR “Physical Activit*” OR “Motor Training” OR “Motor Therapy” OR “Motor Exercise*” OR “Motor Stimulation” OR “Motor Activit*” OR “Aerobic Training*” OR “Aerobic Therapy*” OR “Aerobic Exercise*” OR “Aerobic Rehabilitation” OR “Aerobic Activit*” OR “Strength Training” OR “Strength Exercise*” OR Exercise*):ti,ab,kw) |
|  | 5 | ((Multi-component OR Multicomponent OR Multidomain OR Multi-domain OR Multimodal OR Multi-modal OR Combin* OR Dual-Task OR Dualtask OR “Combined Modality Therapy” OR Simultaneous OR Sequential OR Synergistic OR Exergam*):ti,ab,kw) |
|  | 6 | ((((“Cognitive Motor” OR Cognitive-Motor OR “Motor Cognitive” OR Motor-cognitive) AND (Therapy OR Intervention OR Exercise* OR Rehabilitation)) OR ((Non-pharmacological AND (Cogniti*) AND (Physical OR Motor OR Aerobic OR Strength))) AND (Training OR Exercise OR Therapy OR Treatment OR Intervention) OR Exergam*):ti,ab,kw) |
|  | 7 | #1 AND #2 AND (((#3 AND #4) AND #5) OR #6) |
| Scopus  (Elsevier)  Filter English Language, Inception to 31 Dec 2024: 2184 results | 1 | TITLE-ABS-KEY(Old* OR Elder* OR Aging OR Aged OR “Older Adults” OR “Older-adults”) |
|  | 2 | TITLE-ABS-KEY((Cogniti* AND (Impair* OR Decline OR Dysfunction OR Disorder OR Problem OR Issue)) OR Alzheimer* OR Dementia) |
|  | 3 | TITLE-ABS-KEY ((“Cognitive Behavio#ral Therapy” OR CBT OR “Cognitive Training” OR “Cognitive Therapy” OR “Cognitive Exercise” OR “Cognitive Rehabilitation” OR “Cognitive Intervention” OR “Cognitive Stimulation” OR “Cognitive Method” OR “Cognitive Program*” OR “Cognitive Treatment”)) |
|  | 4 | TITLE-ABS-KEY ((“Physical Training” OR “Physical Therapy” OR “Physical Exercise*” OR “Physical Rehabilitation” OR “Physical Activit*” OR “Motor Training” OR “Motor Therapy” OR “Motor Exercise*” OR “Motor Stimulation” OR “Motor Activit*” OR “Aerobic Training*” OR “Aerobic Therapy*” OR “Aerobic Exercise*” OR “Aerobic Rehabilitation” OR “Aerobic Activit*” OR “Aerobic Activit*” OR “Strength Training” OR “Strength Exercise*” OR Exercise)) |
|  | 5 | TITLE-ABS-KEY ((Multi-component OR Multicomponent OR Multidomain OR Multi-domain OR Multimodal OR Multi-modal OR Combin* OR Dual-Task OR Dualtask OR “Combined Modality Therapy” OR Simultaneous OR Sequential OR Synergistic OR Exergam*)) |
|  | 6 | TITLE-ABS-KEY (((“Cognitive Motor” OR “Cognititve-Motor” OR “Motor-Cognitive” OR “Motor Cognitive”) AND (Therapy OR Intervention OR Exercise* OR Rehabilitation)) OR ((“Non-pharmacological” AND (Cogniti*) AND (Physical OR Motor OR Aerobic OR Strength)) AND (Training OR Exercise OR Therapy OR Treatment OR Intervention)) OR Exergam*) |
|  | 7 | #1 AND #2 AND (((#3 AND #4) AND #5) OR #6) |
| Web of Science Core Collection  (Clarivate)  Filter English Language, Inception to 31 Dec 2024: 1088 results | 1 | TI=(Old* OR Elder* OR Aging OR Aged OR “Older Adults” OR “Older-adults”) OR AB=(Old* OR Elder* OR Aging OR Aged OR “Older Adults” OR “Older-adults”) |
|  | 2 | TI=((Cogniti* AND (Impair* OR Decline OR Dysfunction OR Disorder OR Problem OR Issue)) OR Alzheimer* OR Dementia) OR AB=((Cogniti* AND (Impair* OR Decline OR Dysfunction OR Disorder OR Problem OR Issue)) OR Alzheimer* OR Dementia) |
|  | 3 | TI=((“Cognitive Behavio#ral Therapy” OR CBT OR “Cognitive Training” OR “Cognitive Therapy” OR “Cognitive Exercise” OR “Cognitive Rehabilitation” OR “Cognitive Intervention” OR “Cognitive Stimulation” OR “Cognitive Method” OR “Cognitive Program*” OR “Cognitive Treatment”)) OR AB=((“Cognitive Behavio#ral Therapy” OR CBT OR “Cognitive Training” OR “Cognitive Therapy” OR “Cognitive Exercise” OR “Cognitive Rehabilitation” OR “Cognitive Intervention” OR “Cognitive Stimulation” OR “Cognitive Method” OR “Cognitive Program*” OR “Cognitive Treatment”)) |
|  | 4 | TI=((“Physical Training” OR “Physical Therapy” OR “Physical Exercise*” OR “Physical Rehabilitation” OR “Physical Activit*” OR “Motor Training” OR “Motor Therapy” OR “Motor Exercise*” OR “Motor Stimulation” OR “Motor Activit*” OR “Aerobic Training*” OR “Aerobic Therapy*” OR “Aerobic Exercise*” OR “Aerobic Rehabilitation” OR “Aerobic Activit*” OR “Aerobic Activit*” OR “Strength Training” OR “Strength Exercise*” OR Exercise)) OR AB=((“Physical Training” OR “Physical Therapy” OR “Physical Exercise*” OR “Physical Rehabilitation” OR “Physical Activit*” OR “Motor Training” OR “Motor Therapy” OR “Motor Exercise*” OR “Motor Stimulation” OR “Motor Activit*” OR “Aerobic Training*” OR “Aerobic Therapy*” OR “Aerobic Exercise*” OR “Aerobic Rehabilitation” OR “Aerobic Activit*” OR “Aerobic Activit*” OR “Strength Training” OR “Strength Exercise*” OR Exercise)) |
|  | 5 | TI=((Multi-component OR Multicomponent OR Multidomain OR Multi-domain OR Multimodal OR Multi-modal OR Combin* OR Dual-Task OR Dualtask OR “Combined Modality Therapy” OR Simultaneous OR Sequential OR Synergistic OR Exergam*)) OR AB=((Multi-component OR Multicomponent OR Multidomain OR Multi-domain OR Multimodal OR Multi-modal OR Combin* OR Dual-Task OR Dualtask OR “Combined Modality Therapy” OR Simultaneous OR Sequential OR Synergistic OR Exergam*)) |
|  | 6 | TI=(((“Cognitive Motor” OR “Cognititve-Motor” OR “Motor-Cognitive” OR “Motor Cognitive”) AND (Therapy OR Intervention OR Exercise* OR Rehabilitation)) OR ((“Non-pharmacological” AND (Cogniti*) AND (Physical OR Motor OR Aerobic OR Strength)) AND (Training OR Exercise OR Therapy OR Treatment OR Intervention)) OR Exergam*) OR **AB=**(((“Cognitive Motor” OR “Cognititve-Motor” OR “Motor-Cognitive” OR “Motor Cognitive”) AND (Therapy OR Intervention OR Exercise* OR Rehabilitation)) OR ((“Non-pharmacological” AND (Cogniti*) AND (Physical OR Motor OR Aerobic OR Strength)) AND (Training OR Exercise OR Therapy OR Treatment OR Intervention)) OR Exergam*) |
|  | 7 | #1 AND #2 AND (((#3 AND #4) AND #5) OR #6) |
| PsycInfo  (OVID)  Filter English Language, Inception to 31 Dec 2024: 361 results | 1 | Exp Aging/ or (Old* OR Elder* or Aging or Aged or “Older Adults” or “Older-adults”).tw. |
|  | 2 | Exp cognitive aging/ or exp cognitive impairment/ or exp dementia/ or exp alzheimer’s disease/ or (Cogniti* AND (Impair* or Decline or Dysfunction or Disorder or Problem or Issue) OR Alzheimer* or Dementia).tw. |
|  | 3 | Exp cognitive behavior therapy/ or exp cognitive therapy/ or exp cognitive rehabilitation/ or exp brain training/ or (“Cognitive Behavio#ral Therapy” OR CBT OR “Cognitive Training” or “Cognitive Therapy” or “Cognitive Exercise*” or “Cognitive Rehabilitation” or “Cognitive Intervention” or “Cognitive Stimulation” or “Cognitive Method” or “Cognitive Program*” or “Cognitive Treatment”).tw. |
|  | 4 | Exp exercise/ or exp physical activity/ or (“Physical Training” or “Physical Therapy” or “Physical Exercise*” or “Physical Rehabilitation” or “Physical Activit*” or “Motor Training” or “Motor Therapy” or “Motor Exercise*” or “Motor Stimulation” or “Motor Activit*” or “Aerobic Training*” or “Aerobic Therapy*” or “Aerobic Exercise*” or “Aerobic Rehabilitation” or “Aerobic Activit*” or “Strength Training” or “Strength Exercise*” or “Exercise”).tw. |
|  | 5 | Exp dual task performance/ or exp multimodal treatment approach/ or (Multi-component or Multicomponent or Multidomain or Multi-domain or Multimodal or Multi-modal or Combin* or Dual-Task or Dualtask or “Combined Modality Therapy” or Simultaneous or Sequential or Synergistic or Exergam*).tw. |
|  | 6 | (((“Cognitive Motor” or “Cognitive-Motor” or “Motor-Cognitive” or “Motor-cognitive”) and (Therapy or Intervention or “Exercise*” or Rehabilitation)) or ((“Non-pharmacological” and (Cogniti*) and (Physical or Motor or Aerobic or Strength))) and (Training or Exercise or Therapy or Treatment or Intervention) or Exergam*).tw. |
|  | 7 | #1 AND #2 AND (((#3 AND #4) AND #5) OR #6) |
| ProQuest Dissertations and Thesis  (Clarivate)  Filter English Language, Inception to 31 Dec 2024: 99 results | 1 | TI=(Old* OR Elder* OR Aging OR Aged OR “Older Adults” OR “Older-adults”) OR AB=(Old* OR Elder* OR Aging OR Aged OR “Older Adults” OR “Older-adults”) |
|  | 2 | TI=((Cogniti* AND (Impair* OR Decline OR Dysfunction OR Disorder OR Problem OR Issue)) OR Alzheimer* OR Dementia) OR AB=((Cogniti* AND (Impair* OR Decline OR Dysfunction OR Disorder OR Problem OR Issue)) OR Alzheimer* OR Dementia) |
|  | 3 | TI=((“Cognitive Behavio#ral Therapy” OR CBT OR “Cognitive Training” OR “Cognitive Therapy” OR “Cognitive Exercise” OR “Cognitive Rehabilitation” OR “Cognitive Intervention” OR “Cognitive Stimulation” OR “Cognitive Method” OR “Cognitive Program*” OR “Cognitive Treatment”)) OR AB=((“Cognitive Behavio#ral Therapy” OR CBT OR “Cognitive Training” OR “Cognitive Therapy” OR “Cognitive Exercise” OR “Cognitive Rehabilitation” OR “Cognitive Intervention” OR “Cognitive Stimulation” OR “Cognitive Method” OR “Cognitive Program*” OR “Cognitive Treatment”)) |
|  | 4 | TI=((“Physical Training” OR “Physical Therapy” OR “Physical Exercise*” OR “Physical Rehabilitation” OR “Physical Activit*” OR “Motor Training” OR “Motor Therapy” OR “Motor Exercise*” OR “Motor Stimulation” OR “Motor Activit*” OR “Aerobic Training*” OR “Aerobic Therapy*” OR “Aerobic Exercise*” OR “Aerobic Rehabilitation” OR “Aerobic Activit*” OR “Aerobic Activit*” OR “Strength Training” OR “Strength Exercise*” OR Exercise)) OR AB=((“Physical Training” OR “Physical Therapy” OR “Physical Exercise*” OR “Physical Rehabilitation” OR “Physical Activit*” OR “Motor Training” OR “Motor Therapy” OR “Motor Exercise*” OR “Motor Stimulation” OR “Motor Activit*” OR “Aerobic Training*” OR “Aerobic Therapy*” OR “Aerobic Exercise*” OR “Aerobic Rehabilitation” OR “Aerobic Activit*” OR “Aerobic Activit*” OR “Strength Training” OR “Strength Exercise*” OR Exercise)) |
|  | 5 | TI=((Multi-component OR Multicomponent OR Multidomain OR Multi-domain OR Multimodal OR Multi-modal OR Combin* OR Dual-Task OR Dualtask OR “Combined Modality Therapy” OR Simultaneous OR Sequential OR Synergistic OR Exergam*)) OR AB=((Multi-component OR Multicomponent OR Multidomain OR Multi-domain OR Multimodal OR Multi-modal OR Combin* OR Dual-Task OR Dualtask OR “Combined Modality Therapy” OR Simultaneous OR Sequential OR Synergistic OR Exergam*)) |
|  | 6 | TI=(((“Cognitive Motor” OR “Cognititve-Motor” OR “Motor-Cognitive” OR “Motor Cognitive”) AND (Therapy OR Intervention OR Exercise* OR Rehabilitation)) OR ((“Non-pharmacological” AND (Cogniti*) AND (Physical OR Motor OR Aerobic OR Strength)) AND (Training OR Exercise OR Therapy OR Treatment OR Intervention)) OR Exergam*) OR **AB=**(((“Cognitive Motor” OR “Cognititve-Motor” OR “Motor-Cognitive” OR “Motor Cognitive”) AND (Therapy OR Intervention OR Exercise* OR Rehabilitation)) OR ((“Non-pharmacological” AND (Cogniti*) AND (Physical OR Motor OR Aerobic OR Strength)) AND (Training OR Exercise OR Therapy OR Treatment OR Intervention)) OR Exergam*) |
|  | 7 | #1 AND #2 AND (((#3 AND #4) AND #5) OR #6) |

# **Appendix A5.** Data Extraction Form

| References (Author/ Year of publication): |  |
| --- | --- |
| Objectives: |  |
| Sample size/ NCD nature: |  |
| Total trials: |  |
| Trials that met this UR's eligibility criteria: |  |
| Comparators (Active/ Passive): |  |
| Measured outcomes in relation to PICO (Global cog, ADL, Gait, Balance, HRQoL), and affiliated directional effects: |  |
| Review typology: |  |
| Gender included: |  |
| Age (range or mean or median or minimum): |  |
| Number of databases searched: |  |
| Search period: |  |
| Publication range of year: |  |
| Publication language: |  |
| Quality appraisal instruments/ Certainty of Evidence: |  |
| Study affiliation |  |
| Funding: |  |
| Publisher: |  |
| Protocol Registration Availability: |  |

# **Appendix A6.** Critical Appraisal for Systematic Reviews Using AMSTAR-2

| **Authors** | **Q1** | **Q2*** | **Q3** | **Q4*** | **Q5** | **Q6** | **Q7*** | **Q8** | **Q9*^a^** | **Q9^b^** | **Q10** | **Q11*^a^** | **Q11^b^** | **Q12** | **Q13*** | **Q14** | **Q15*** | **Q16** | **Overall confidence** |
| --- | --- | --- | --- | --- | --- | --- | --- | --- | --- | --- | --- | --- | --- | --- | --- | --- | --- | --- | --- |
| Ali et al. (2022) | Y | Y | Y | Y | Y | Y | N | PY | Y | NA | N | Y | NA | Y | Y | Y | Y | Y | Low |
| Cai et al. (2023) | Y | Y | N | PY | N | Y | N | PY | Y | NA | N | Y | NA | N | N | Y | N | Y | Critically low |
| Cai et al. (2024) | Y | PY | Y | PY | Y | Y | N | PY | Y | NA | N | Y | NA | Y | Y | Y | Y | Y | Low |
| Chan et al. (2024) | Y | Y | N | Y | Y | Y | Y | Y | Y | NA | N | Y | NA | Y | Y | Y | Y | Y | Moderate |
| Gómez-Soria et al. (2022) | Y | Y | N | Y | Y | Y | N | Y | Y | Y | N | Y | N | Y | Y | Y | Y | Y | Low |
| Han et al. (2022) | Y | Y | N | Y | Y | Y | N | Y | Y | Y | N | Y | N | Y | Y | Y | N | Y | Critically Low |
| Hong et al. (2024) | Y | Y | Y | PY | Y | Y | Y | PY | Y | NA | N | Y | NA | Y | Y | Y | Y | Y | High |
| Karssemeijer et al. (2017) | Y | Y | Y | PY | Y | N | N | PY | Y | NA | N | Y | NA | Y | Y | Y | Y | Y | Low |
| Li et al. (2022) | Y | Y | N | PY | Y | Y | N | PY | Y | NA | N | Y | NA | Y | Y | Y | N | Y | Critically Low |
| Liu et al. (2023) | Y | Y | Y | PY | Y | N | N | PY | Y | NA | N | Y | NA | Y | Y | Y | Y | Y | Low |
| Meng et al. (2022) | Y | PY | N | PY | Y | Y | N | Y | Y | NA | N | Y | NA | N | N | Y | N | Y | Critically Low |
| Wati et al. (2024) | Y | Y | N | PY | Y | Y | N | PY | Y | NA | N | Y | NA | Y | Y | Y | N | Y | Critically Low |
| Xu et al. (2021) | Y | Y | Y | PY | Y | Y | N | PY | Y | NA | N | Y | NA | N | N | Y | Y | Y | Critically Low |
| Xue et al. (2023) | Y | Y | N | PY | Y | Y | N | PY | Y | NA | N | Y | NA | Y | Y | Y | Y | Y | Low |
| Ye et al. (2024) | Y | Y | Y | Y | N | Y | N | Y | Y | NA | N | Y | NA | Y | Y | Y | Y | Y | Low |
| Zhao et al. (2022) | Y | Y | Y | Y | Y | Y | N | PY | Y | NA | N | Y | NA | N | Y | Y | Y | Y | Low |
| Zhu et al. (2021) | Y | PY | Y | PY | Y | N | N | PY | Y | NA | N | Y | NA | N | N | Y | Y | Y | Critically Low |
| ***Number of Y/PY*** | 17 | 17 | 9 | 17 | 15 | 14 | 2 | 17 | 17 | 2 | 0 | 17 | 0 | 12 | 13 | 17 | 12 | 17 |  |

*Note:* ^a^ = Randomized controlled trials; ^b^ = Non-randomized studies of intervention, Y = Yes, PY = Partial Yes, N = No, NA = Non-applicable;

**AMSTAR-2 16-item: (*** = Critical domain)**:** Q1=Components of PICO question? Q2*=Review protocol? Q3=Explanation of study design? Q4* =Comprehensive literature searching strategy? Q5=Study selection in duplicate? Q6 = Data extraction in duplicate? Q7*=List of excluded studies and justify the exclusions? Q8=Study characteristics? Q9* =Satisfactory technique for assessing risk of bias? Q10=Sources of funding for each study? Q11*=Appropriate methods? Q12=Assess potential impact of risk of bias on the results? Q13*=Account for risk of bias when interpreting/discussing? Q14=Satisfactory explanation for and discussion of any heterogeneity? Q15*=Publication bias (small sample bias) assessed and discussed? Q16= Potential sources of conflict of interest?

**Overall confidence:** High = no or 1 non-critical weakness; Moderate = > 1 non-critical weakness; Low = 1 critical flaw with or without non-critical weakness; Critically low = > 1 critical flaw with or without non-critical weakness

# **Appendix A7.1.** Algorithm for ‘‘Downgrades’’ to Levels of Evidence in Reviews

| Area assessed |  | Imprecision |  | Risk of bias (study quality) |  | Inconsistency |  | Risk of bias (review quality) |
| --- | --- | --- | --- | --- | --- | --- | --- | --- |
| Method of assessment |  | Number of participants within pooled analysis |  | Proportion of participants included in the pooled analysis judged to have low ROB for randomization and observer blinding |  | Heterogeneity, assessed by *I*^2^ statistic |  | Responses to AMSTAR-2’s 7 critical questions (review protocol, comprehensive search strategy, list of excluded studies and justifications, satisfactory technique for assessing ROB, appropriate methods for statistical combination of results for MA, accounted for ROB when discussing, publication bias assessed and discussed) |
| No downgrade  (No serious limitations) |  | ≥200 |  | ≥75% of participants have low ROB |  | *I*^2^ ≤ 75% |  | 7/7 are all “yes” |
| Downgrade 1 level  (Serious limitations) |  | 100-199 |  | <75% of participants have low ROB |  | *I*^2^ > 75% |  | 6/7 are “yes” and 1 is “unclear” or “no” on AMSTAR-2 |
| Downgrade 2 levels  (very serious limitations) |  | 1-99 |  |  |  |  |  | < 6/7 are “yes” and remainder are “unclear” or “no” on AMSTAR-2 |
| Notes |  |  |  | If ROB for individual trials was not reported within the review, we were conservative and assumed that less than 75% of participants had low ROB. |  | If only one trial contributed to analysis, no downgrade; if *I*^2^ not reported, assumed to be greater than 75%. |  |  |

*Note*. AMSTAR: AMSTAR-2 quality assessment tool; MA: Meta-analysis; PICO: Population, intervention, control, outcome; ROB: Risk of bias

Indirectness was not indicated to be marked for downgrading but was still subjected to qualitative assessment of reviews and quantitative assessment of meta-analysis. Downgrades were specifically applied as follows: (1) Imprecision was downgraded by one level if 100-199 participants were included in the pooled analysis, and two levels if <100 participants; (2) Risk of bias at the included study level was downgraded by one level if >75% of studies were high risk of bias; (3) Inconsistency was downgraded by one level if *I*^2^ statistics were ≥75%; (4) Risk of bias at the review level was downgraded by one level if one critical domain was negative, and two levels if ≥2 were negative.

# **Appendix A7.2.** Formula for GRADE Level of Evidence from Number of Downgrades Determined Using the Algorithm

| **GRADE level of evidence** | **Number of downgrades (derived from objective assessment)** |
| --- | --- |
| High | 0 downgrade |
| Moderate | 1 or 2 downgrades |
| Low | 3 or 4 downgrades |
| Very low | 5 or 6 downgrades |

GRADE: Grading of recommendations assessment, development and evaluation

# **Appendix A8.** Lists of Excluded Reviews with Exclusion Reasons

| DOI: | Authors | Title | Reason for exclusion |
| --- | --- | --- | --- |
| DOI:10.2196/43607 | Abd-alrazaq et al. (2023) | Serious Games for Learning among Older Adults with Cognitive Impairment: Systematic Review and Meta-analysis | Intervention not eligible; No PCDT intervention |
| DOI:10.1177/01939459211032272 | Ahn et al. (2022) | The Effects of Multi-Domain Interventions on Cognition: A Systematic Review | Population not eligible; <50% cognitive impaired |
| DOI:10.1016/j.arr.2018.04.002 | Bruderer-Hofstetter et al. (2022) | Effective multicomponent interventions in comparison to active control and no interventions on physical capacity, cognitive function and instrumental activities of daily living in elderly people with and without mild impaired cognition - A systematic review and network meta-analysis | Population not eligible; <50% cognitive impaired |
| DOI:10.3389/fnagi.2023.1191729 | Buele et al. (2023) | Cognitive-motor interventions based on virtual reality and instrumental activities of daily living (iADL): an overview | Study type not eligible: No Meta-Analysis |
| DOI:10.3389/fneur.2022.903673 | Buyle et al. (2022) | The role of motivation factors in exergame interventions for fall prevention in older adults: A systematic review and meta-analysis | Population not eligible; Healthy older adults |
| DOI:10.1002/pmrj.12444 | Cugusi et al. (2020) | Exergaming for Quality of Life in Persons Living with Chronic Diseases: A Systematic Review and Meta-analysis | Population not eligible; <50% cognitive impaired |
| DOI:10.1016/j.neubiorev.2021.07.008 | Dhir et al. (2021) | The Effects of Combined Physical and Cognitive Training on Inhibitory Control: A Systematic Review and Meta-Analysis | Population not eligible; <50% cognitive impaired |
| DOI:10.1186/s13643-020-01555-8 | Faieta et al. (2021) | Exercise interventions for older adults with Alzheimer’s disease: a systematic review and meta-analysis protocol | Study type not eligible: Protocol |
| DOI:10.1016/j.arr.2020.101232 | Gavelin et al. (2020) | Combined physical and cognitive training for older adults with and without cognitive impairment: A systematic review and network meta-analysis of randomized controlled trials | Population not eligible; <50% cognitive impaired |
| DOI:10.2147/CIA.S125201 | Ghai et al. (2017) | Effects of dual tasks and dual-task training on postural stability: A systematic review and meta-analysis | Population not eligible; <50% cognitive impaired |
| DOI:10.1186/s12966-018-0697-x | Gheysen et al. (2018) | Physical activity to improve cognition in older adults: Can physical activity programs enriched with cognitive challenges enhance the effects? A systematic review and meta-analysis | Population not eligible; <50% cognitive impaired |
| DOI:10.3390/ijerph17176166 | Guo et al. (2020) | Effect of combined physical and cognitive interventions on executive functions in older adults: A meta-analysis of outcomes | Population not eligible; <50% cognitive impaired |
| DOI:10.7717/peerj.13194 | Jiang et al. (2022) | Effects of exergaming on executive function of older adults: a systematic review and meta-analysis | Population not eligible; <50% cognitive impaired |
| DOI:10.3390/jpm12020276 | Kiper et al. (2022) | Combined Motor and Cognitive Rehabilitation: The Impact on Motor Performance in Patients with Mild Cognitive Impairment. Systematic Review and Meta-Analysis | Unable to retrieve full data; characteristics of included studies locked behind missing appendices |
| DOI:10.1155/2019/2308475 | Li et al. (2019) | Effectiveness of Exercise Programs on Patients with Dementia: A Systematic Review and Meta-Analysis of Randomized Controlled Trials | Intervention not eligible; No PCDT intervention |
| DOI:10.23736/s1973-9087.17.04680-9 | Mura et al. (2017) | Active exergames to improve cognitive functioning in neurological disabilities: a systematic review and meta-analysis | Population not eligible; <50% cognitive impaired |
| DOI:10.1093/ageing/afac251 | Ning et al. (2022) | Older adults' experiences of implementing exergaming programs: a systematic review and qualitative meta-synthesis | Outcome not eligible; Irrelevant objective |
| DOI:10.1002/14651858.CD011961.pub2 | Orgeta et al. (2020) | Cognitive training interventions for dementia and mild cognitive impairment in Parkinson’s disease | Population not eligible; Parkinson's |
| DOI:10.1007/s00415-020-09918-w | Prosperini et al. (2020) | Exergames for balance dysfunction in neurological disability: a meta-analysis with meta-regression | Population not eligible; <50% cognitive impaired |
| DOI:10.1016/j.neubiorev.2017.04.011 | Stanmore et al. (2017) | The effect of active video games on cognitive functioning in clinical and non-clinical populations: A meta-analysis of randomized controlled trials | Population not eligible; <50% cognitive impaired |
| DOI:10.1155/2021/8882961 | Sun et al. (2021) | Effects of Combined Physical Activity and Cognitive Training on Cognitive Function in Older Adults with Subjective Cognitive Decline: A Systematic Review and Meta-Analysis of Randomized Controlled Trials | Population not eligible; Subjective Cognitive Decline |
| DOI:10.3389/fpsyg.2022.837710 | Teraz et al. (2022) | Impact of Motor-Cognitive Interventions on Selected Gait and Balance Outcomes in Older Adults: A Systematic Review and Meta-Analysis of Randomized Controlled Trials | Population not eligible; Healthy older adults |
| DOI:10.3389/fnagi.2021.808539 | Torre et al. (2022) | A Review of Combined Training Studies in Older Adults According to a New Categorization of Conventional Interventions | Population not eligible; Healthy older adults |
| DOI:10.1108/JHR-11-2018-0135 | Untari et al. (2019) | A combination of cognitive training and physical exercise for elderly with the mild cognitive impairment: A systematic review | Outcomes not eligible; Objectives did not correlate with findings; Unclear data reporting |
| DOI:10.1186/s12877-022-03302-1 | Venegas-Sanabria et al. (2022) | Effect of multicomponent exercise in cognitive impairment: a systematic review and meta-analysis | Intervention not eligible; No PCDT intervention |
| DOI:10.1007/s40520-019-01142-5 | Wang et al. (2019) | Efficacy of different types of exercises on global cognition in adults with mild cognitive impairment: a network meta-analysis | Intervention not eligible; No PCDT intervention |
| DOI:10.1002/gps.5289 | Wang et al. (2020) | Effects of non-pharmacological therapies for people with mild cognitive impairment. A Bayesian network meta-analysis | Outcome not eligible; Unclear dual-task intervention results |
| DOI:10.1016/j.ajp.2023.103635 | Wang et al. (2023) | A systematic review and network meta-analysis comparing various non-pharmacological treatments for older people with mild cognitive impairment | Intervention not eligible; No PCDT intervention |
| DOI:10.1186/s11556-020-00240-y | Wollesen et al. (2022) | The effects of cognitive-motor training interventions on executive functions in older people: A systematic review and meta-analysis | Population not eligible; Healthy older adults |
| DOI:10.7717/peerj.15108 | Wu et al. (2023) | Does the combination of exercise and cognitive training improve working memory in older adults? A systematic review and meta-analysis | Population not eligible; <50% cognitive impaired |
| DOI:10.1016/j.jamda.2021.03.009 | Yen et al. (2021) | Virtual Reality Exergames for Improving Older Adults’ Cognition and Depression: A Systematic Review and Meta-Analysis of Randomized Control Trials | Population not eligible; <50% cognitive impaired |
| DOI:10.1371/journal.pone.0308466 | Yi et al. (2024) | Cognitive and physical impact of combined exercise and cognitive intervention in older adults with mild cognitive impairment: A systematic review and meta-analysis | Unclear data reporting; Unclear meta-analyses reporting |
| DOI:10.1016/j.gerinurse.2024.02.032 | Yu et al. (2024) | Virtual reality exergames for improving physical function, cognition and depression among older nursing home residents: A systematic review and meta-analysis | Population not eligible; <50% cognitive impaired |
| DOI:10.1093/ageing/afac175 | Zhang et al. (2022) | Effectiveness of exergaming-based interventions for mobility and balance performance in older adults with Parkinson's disease: systematic review and meta-analysis of randomised controlled trials | Population not eligible; Parkinson's |

PCDT: Physio-cognitive dual-task training

# **Appendix A9.** Lists of Excluded Primary Studies from Corresponding Study-Level Meta-Analyses with Exclusion Reasons

| Outcomes | Studies/ Authors | Reasons for exclusion |
| --- | --- | --- |
| Global cognition | Han et al. (2017) | Employed a cross-over study but did not report pre-cross-over effect sizes. |
|  | Olazarán et al. (2004) | Did not clearly report global cognition changes. |
| HRQoL | Hagovska et al. (2016) | The corresponding review by Hong et al. (2024) reported values lifted from another study conducted by Hagovská & Olekszyová (2016), but referenced as Hagovska et al. (2016), along with the incorrect references. |
| ADL | Han et al. (2017) | Employed a cross-over study but did not report pre-cross-over effect sizes. |
| Gait | Karssemeijer et al. (2019b) | Incomplete reporting of gait/ TUG data. |

ADL: Activities of daily living; HRQoL: Health-related quality of life; TUG: Timed-up-and-go.

# **Appendix A10.** Summary of Affiliation, Funding, Publisher, Protocol Registration of Included Reviews

| References | Study affiliation | Funding | Publisher | Protocol Registration Availability |
| --- | --- | --- | --- | --- |
| Ali et al. (2022) | Nanjing Medical University. | Funded by: National Natural Science Foundation of China, National Key R&D Program of China, Nanjing Municipal Science and Technology Bureau | The Journal of Prevention of Alzheimer's Disease. | Yes |
| Cai et al. (2023) | Harbin Medical University. | Not funded. | Geriatric Nursing. | Yes |
| Cai et al. (2024) | Beijing Sports University. | Funded by: National Key Research and Development Program of China. | Frontiers in Neurology. | Not registered |
| Chan et al. (2024) | Chinese University of Hong Kong. | Not funded. | npj Digital Medicine. | Yes |
| Gómez-Soria et al. (2022) | University of Zaragoza. | Not funded. | Archives of Gerontology and Geriatrics. | Yes |
| Han et al. (2022) | Capital Medical University. | Funded by: National Key Research and Development Program of China, Capital Health Research and Development of Special Fund, China Rehabilitation Research Center Key Project. | Frontiers in Aging Neuroscience. | Yes |
| Hong et al. (2024) | National University of Singapore. | Not funded. | Archives of Gerontology and Geriatrics. | Yes |
| Karssemeijer et al. (2017) | Radboud University Medical Centre. | Funded by: Netherlands Organisation for Health  Research and Development (ZonMw). | Ageing Research Reviews. | Yes |
| Li et al. (2022) | Guangxi Medical University. | Not funded. | Complementary Therapies in Medicine. | Yes |
| Liu et al. (2023) | Shandong University. | Not funded. | Aging Clinical and Experimental Research. | Yes |
| Meng et al. (2020) | Jilin University. | Funded by: Health and Family Planning Committee of Jilin Province China, Education Department of Jilin Province China. | Aging Clinical and Experimental Research. | Not registered |
| Wati et al. (2024) | National Cheng Kung University. | Not funded. | Narra J | Yes |
| Xu et al. (2021) | Chinese University of Hong Kong. | Not funded. | Frontiers in Aging Neuroscience. | Yes |
| Xue et al. (2023) | Hong Kong University. | Not funded. | International Journal of Nursing Studies. | Yes |
| Ye et al. (2024) | Taipei Medical University. | Not funded. | International Journal of Nursing Studies. | Yes |
| Zhao et al. (2022) | Nursing School of Peking University. | Funded by: National Natural Science Foundation of China. | Aging & Mental Health. | Yes |
| Zhu et al. (2021) | Nanjing Medical University. | Funded by: National Key R&D Program of China. | Frontiers in Aging Neuroscience. | Not registered |

# **Appendix A11.1.** Between-Studies Overlaps for Meta-Analyses Measuring Outcomes Relevant to this Umbrella Review

|  | Systematic reviews and meta-analyses included in the umbrella review (n=17) | | | | | | | | | | | | | | | | |
| --- | --- | --- | --- | --- | --- | --- | --- | --- | --- | --- | --- | --- | --- | --- | --- | --- | --- |
| Primary Studies (n = 81) | Ali et al. (2022) | Cai et al. (2023) | Cai et al. (2024) | Chan et al. (2024) | Gómez-Soria et al. (2022) | Han et al. (2022) | Hong et al. (2024) | Karssemeijer et al. (2017) | Li et al. (2022) | Liu et al. (2023) | Meng et al. (2020) | Wati et al. (2024) | Xu et al. (2021) | Xue et al. (2023) | Ye et al. (2024) | Zhao et al. (2022) | Zhu et al. (2021) |
| Amjad et al. (2019) |  | X | X |  |  |  |  |  |  |  | X |  |  |  |  |  |  |
| Bae et al. (2019) |  |  |  |  |  |  |  |  |  | X |  |  | X |  |  |  |  |
| Binns et al. (2020) |  |  |  |  |  |  | X |  |  |  |  | X |  | X |  |  |  |
| Bischoff et al. (2021) |  |  |  |  |  |  |  |  |  |  |  |  |  |  | X |  |  |
| Burgener et al. (2008) |  |  |  |  |  |  |  | X |  |  |  |  |  |  |  |  |  |
| Cai & Zhang (2019) |  |  |  |  |  |  |  |  |  |  |  |  |  | X |  |  |  |
| Callisaya et al. (2021) |  |  |  |  |  |  |  |  |  |  |  |  |  |  | X |  |  |
| Chen et al. (2018) | X |  |  |  |  |  |  |  |  |  |  |  |  |  |  |  |  |
| Cintoli et al. (2021) |  |  |  |  |  |  | X |  |  |  |  |  |  |  |  |  |  |
| Combourieu-Donnezan et al. (2018) | X |  |  |  |  |  |  |  |  |  |  | X |  |  | X |  |  |
| Delbroek et al. (2017) | X |  | X | X |  | X | X |  |  |  | X |  |  | X | X | X | X |
| Fiatarone et al. (2014) |  |  |  |  |  |  | X | X |  |  | X |  |  | X |  | X |  |
| Gill et al. (2016) | X |  |  |  |  |  |  |  |  |  |  |  |  |  |  |  |  |
| Graessel et al. (2011) |  |  |  |  |  |  | X | X |  |  |  |  |  |  |  |  |  |
| Hagovska & Nagyova (2017) |  |  |  |  |  |  |  |  |  |  | X |  | X | X |  |  |  |
| Hagovská & Olekszyová (2016) |  |  |  |  | X |  |  |  |  |  |  | X | X |  |  | X |  |
| Hagovska et al. (2016) |  |  |  |  |  |  | X |  |  |  |  |  |  |  | X |  |  |
| Han et al. (2017) |  |  |  |  |  |  |  | X |  |  |  |  |  |  |  |  |  |
| Holthoff et al. (2015) |  |  |  |  |  |  |  | X |  |  |  |  |  |  |  |  |  |
| Hu et al. (2018) |  |  | X |  |  |  |  |  |  |  |  |  |  |  |  |  |  |
| Hughes et al. (2014) |  |  |  | X |  |  |  |  |  |  | X |  |  | X |  |  | X |
| Hwang & Lee (2017) |  |  |  |  |  |  |  |  |  |  |  |  |  |  |  |  | X |
| Jeong et al. (2021) |  |  |  |  |  |  | X |  |  |  |  |  |  | X |  |  |  |
| Jiao et al. (2022) |  |  |  |  |  |  |  |  |  |  |  |  |  | X |  |  |  |
| Karssemeijer et al. (2019a) |  | X |  |  |  |  |  |  |  |  |  |  |  |  |  |  |  |
| Karssemeijer et al. (2019b) |  |  |  |  |  |  | X |  |  |  |  |  |  |  |  |  |  |
| Kounti et al. (2011) |  |  |  |  | X | X |  |  |  |  |  |  |  |  |  |  |  |
| Kouzuki et al. (2020) |  |  |  |  |  |  | X |  |  |  |  |  |  |  |  |  |  |
| Kuo et al. (2022) |  |  |  |  |  |  |  |  |  |  |  |  |  |  | X |  |  |
| Lam et al. (2015) |  |  |  |  |  | X |  |  |  | X | X |  | X | X |  |  |  |
| Lam et al. (2022) |  |  |  |  |  |  | X |  |  |  |  |  |  |  |  |  |  |
| Law et al. (2019) |  |  |  |  |  |  | X |  |  |  | X |  |  |  |  |  |  |
| Lemke et al. (2019) |  |  |  |  |  |  |  |  |  |  |  |  |  |  | X |  |  |
| Li et al. (2022) |  |  |  |  |  |  |  |  |  | X |  |  |  |  | X |  |  |
| Liao et al. (2020) |  | X |  | X |  |  |  |  |  |  |  |  |  |  |  | X |  |
| Liao et al. (2019) |  |  |  |  |  |  |  |  |  |  |  |  |  |  |  |  | X |
| Liu et al. (2022) |  | X |  | X |  |  |  |  |  | X |  |  |  |  |  |  |  |
| Luttenberger et al. (2015) |  |  |  |  |  |  | X |  |  |  |  |  |  |  |  |  |  |
| Maci et al. (2012) |  |  |  |  |  |  | X |  |  |  |  |  |  |  |  |  |  |
| Maffei et al. (2017) | X |  |  |  |  |  | X | X |  |  |  |  |  |  |  |  |  |
| Makizako et al. (2012) | X |  |  |  |  |  |  |  |  |  |  |  |  |  |  |  |  |
| Menengiç et al. (2022) |  |  |  |  |  |  |  |  |  |  |  |  |  |  | X |  |  |
| Min-Ki et al. (2019) |  |  |  |  |  |  |  |  |  |  |  |  |  |  |  | X |  |
| Mrakic-Sposta et al. (2018) |  |  |  | X |  | X | X |  |  |  |  |  |  | X | X | X |  |
| Muñiz et al. (2015) |  |  |  |  |  |  | X |  |  |  |  |  |  |  |  |  |  |
| Nam & Kim (2021) |  |  |  |  |  |  |  |  |  |  |  |  |  |  | X |  |  |
| Obman et al. (2016) | X |  |  |  |  |  |  |  |  |  |  |  |  |  |  |  |  |
| Okamura et al. (2018) |  |  |  |  |  |  | X |  |  |  |  |  |  | X |  | X |  |
| Olazarán et al. (2004) |  |  |  |  |  |  |  | X |  |  |  |  |  |  |  |  |  |
| Padala et al. (2012) |  | X |  | X |  |  |  |  |  |  |  |  |  |  |  |  | X |
| Padala et al. (2017) |  | X |  | X |  |  |  |  |  |  |  |  |  |  |  |  |  |
| Parial et al. (2022) |  |  |  |  |  |  |  |  |  |  |  |  |  |  | X |  |  |
| Park (2017) |  |  |  |  |  | X |  |  |  |  |  |  |  |  |  |  |  |
| Park et al. (2019) | X |  |  |  |  | X | X |  |  | X |  |  |  | X | X | X |  |
| Park et al. (2020) |  |  | X |  |  | X |  |  |  |  | X |  |  |  |  |  |  |
| Reuter et al. (2012) |  |  |  |  |  |  | X |  |  |  |  |  |  |  |  |  |  |
| Rojasavastera et al. (2020) |  |  |  |  |  | X |  |  |  |  |  |  |  |  |  |  |  |
| Santos et al. (2015) |  |  |  |  |  |  |  | X |  |  |  |  |  |  |  |  |  |
| Schwenk et al. (2010) |  |  |  |  |  |  |  |  |  |  |  |  |  |  | X |  |  |
| Schwenk et al. (2016) |  |  |  | X |  |  |  |  |  |  | X |  |  |  |  |  | X |
| Shimada et al. (2018) | X |  |  |  |  |  | X |  |  |  | X |  | X | X | X | X |  |
| Siu & Lee (2018) |  |  |  |  |  |  |  |  | X |  |  |  |  |  |  |  |  |
| Straubmeier et al. (2017) |  |  |  |  |  |  | X |  |  |  |  |  |  |  |  |  |  |
| Styliadis et al. (2015) |  |  |  |  | X |  |  |  |  |  |  |  |  |  |  |  |  |
| Suzuki et al. (2012) | X |  |  |  |  |  |  |  |  | X |  |  |  |  |  |  |  |
| Suzuki et al. (2013) | X |  |  |  |  |  |  | X |  | X | X |  |  | X |  | X |  |
| Swinnen et al. (2021) |  |  |  |  |  |  |  |  |  |  |  |  |  |  | X |  |  |
| Tanaka et al. (2020) |  |  |  |  |  |  |  |  |  |  |  |  |  | X |  |  |  |
| Tarnanas et al. (2014) |  |  | X | X |  |  |  |  |  |  |  |  |  |  |  |  | X |
| Tarnanas et al. (2015) |  |  | X |  |  |  |  |  |  |  |  |  |  |  |  |  |  |
| Thapa et al. (2020) |  |  | X | X |  |  |  |  |  |  |  |  |  |  | X |  | X |
| Torpil et al. (2021) |  | X |  | X |  |  |  |  |  |  |  |  |  |  |  |  |  |
| Tsai et al. (2013) |  |  |  |  |  |  |  |  | X |  |  |  |  |  |  |  |  |
| Van-Santen et al. (2020) |  | X |  | X |  |  |  |  |  |  |  |  |  | X | X |  |  |
| Venturelli et al. (2016) | X |  |  |  |  |  |  | X |  |  |  |  |  | X |  | X |  |
| Xu et al. (2020) |  |  |  |  |  |  |  |  | X | X | X |  |  |  |  |  |  |
| Yang et al. (2022) |  |  |  |  |  |  |  |  |  | X |  |  |  |  |  |  |  |
| Young (2020) |  |  |  |  |  |  |  |  | X |  |  |  |  | X |  |  |  |
| Young et al. (2019) |  |  |  |  |  |  |  |  | X |  |  |  |  | X |  |  |  |
| Zhang (2019) |  |  |  |  |  |  |  |  |  |  |  |  |  | X |  |  |  |
| Zheng et al. (2022) |  | X |  | X |  |  |  |  |  |  |  |  |  |  |  |  |  |
| Total | 12 | 9 | 7 | 13 | 3 | 8 | 21 | 10 | 5 | 9 | 12 | 3 | 5 | 20 | 18 | 11 | 8 |
| % Overlap | 38/81 = 46.91% | | | | | | | | | | | | | | | | |
| Covered Area (CA) | (174/17*81) = 174/1377= 0.13 | | | | | | | | | | | | | | | | |
| Corrected Covered Area (CCA) | (174-81)/(1377-81) = 93/1296 = 0.0717 = 7.17% = Moderate (6-10%) | | | | | | | | | | | | | | | | |

# **Appendix A11.2.** Within-Studies Overlaps for Global Cognitive Outcomes Using PCDT

|  | Systematic reviews and meta-analyses included in the umbrella review (n = 16) | | | | | | | | | | | | | | | |
| --- | --- | --- | --- | --- | --- | --- | --- | --- | --- | --- | --- | --- | --- | --- | --- | --- |
| Primary Studies (n = 67) | Ali et al. (2022) | Cai et al. (2023) | Cai et al. (2024) | Chan et al. (2024) | Gómez-Soria et al. (2022) | Han et al. (2022) | Hong et al. (2024) | Karssemeijer et al. (2017) | Li et al. (2022) | Liu et al. (2023) | Meng et al. (2020) | Xu et al. (2021) | Xue et al. (2023) | Ye et al. (2024) | Zhao et al. (2022) | Zhu et al. (2021) |
| Amjad et al. (2019) |  | X | X |  |  |  |  |  |  |  | X |  |  |  |  |  |
| Bae et al. (2019) |  |  |  |  |  |  |  |  |  | X |  | X |  |  |  |  |
| Binns et al. (2020) |  |  |  |  |  |  | X |  |  |  |  |  | X |  |  |  |
| Burgener et al. (2008) |  |  |  |  |  |  |  | X |  |  |  |  |  |  |  |  |
| Cai & Zhang (2019) |  |  |  |  |  |  |  |  |  |  |  |  | X |  |  |  |
| Delbroek et al. (2017) | X |  | X | X |  | X | X |  |  |  | X |  | X | X | X | X |
| Fiatarone et al. (2014) |  |  |  |  |  |  | X | X |  |  | X |  | X |  | X |  |
| Gill et al. (2016) | X |  |  |  |  |  |  |  |  |  |  |  |  |  |  |  |
| Graessel et al. (2011) |  |  |  |  |  |  | X | X |  |  |  |  |  |  |  |  |
| Hagovska & Nagyova (2017) | X |  |  |  |  |  |  |  |  |  | X | X | X |  |  |  |
| Hagovská & Olekszyová (2016) |  |  |  |  | X |  |  |  |  |  |  | X |  |  | X |  |
| Hagovska et al. (2016) |  |  |  |  |  |  | X |  |  |  |  |  |  | X |  |  |
| Han et al. (2017) |  |  |  |  |  |  |  | X |  |  |  |  |  |  |  |  |
| Holthoff et al. (2015) |  |  |  |  |  |  |  | X |  |  |  |  |  |  |  |  |
| Hu et al. (2018) |  |  | X |  |  |  |  |  |  |  |  |  |  |  |  |  |
| Hughes et al. (2014) |  |  |  | X |  |  |  |  |  |  | X |  | X |  |  | X |
| Jeong et al. (2021) |  |  |  |  |  |  | X |  |  |  |  |  | X |  |  |  |
| Jiao et al. (2022) |  |  |  |  |  |  |  |  |  |  |  |  | X |  |  |  |
| Kounti et al. (2011) |  |  |  |  | X | X |  |  |  |  |  |  |  |  |  |  |
| Kouzuki et al. (2020) |  |  |  |  |  |  | X |  |  |  |  |  |  |  |  |  |
| Lam et al. (2015) |  |  |  |  |  | X |  |  |  | X | X | X | X |  |  |  |
| Lam et al. (2022) |  |  |  |  |  |  | X |  |  |  |  |  |  |  |  |  |
| Law et al. (2019) |  |  |  |  |  |  | X |  |  |  | X |  |  |  |  |  |
| Li et al. (2022) |  |  |  |  |  |  |  |  |  | X |  |  |  | X |  |  |
| Liao et al. (2020) |  | X |  | X |  |  |  |  |  |  |  |  |  |  | X |  |
| Liu et al. (2022) |  | X |  | X |  |  |  |  |  | X |  |  |  |  |  |  |
| Maci et al. (2012) |  |  |  |  |  |  | X |  |  |  |  |  |  |  |  |  |
| Maffei et al. (2017) | X |  |  |  |  |  | X | X |  |  |  |  |  |  |  |  |
| Menengi et al. (2022) |  |  |  |  |  |  |  |  |  |  |  |  |  | X |  |  |
| Min-Ki et al. (2019) |  |  |  |  |  |  |  |  |  |  |  |  |  |  | X |  |
| Mrakic-Sposta et al. (2018) |  |  |  | X |  | X | X |  |  |  |  |  | X | X | X |  |
| Muñiz et al. (2015) |  |  |  |  |  |  | X |  |  |  |  |  |  |  |  |  |
| Nam & Kim (2021) |  |  |  |  |  |  |  |  |  |  |  |  |  | X |  |  |
| Obman et al. (2016) | X |  |  |  |  |  |  |  |  |  |  |  |  |  |  |  |
| Okamura et al. (2018) |  |  |  |  |  |  | X |  |  |  |  |  | X |  | X |  |
| Olazarán et al. (2004) |  |  |  |  |  |  |  | X |  |  |  |  |  |  |  |  |
| Padala et al. (2012) |  | X |  | X |  |  |  |  |  |  |  |  |  |  |  | X |
| Padala et al. (2017) |  | X |  | X |  |  |  |  |  |  |  |  |  |  |  |  |
| Parial et al. (2022) |  |  |  |  |  |  |  |  |  |  |  |  |  | X |  |  |
| Park (2017) |  |  |  |  |  | X |  |  |  |  |  |  |  |  |  |  |
| Park et al. (2019) | X |  |  |  |  | X | X |  |  | X |  |  | X | X | X |  |
| Park et al. (2020) |  |  | X |  |  | X |  |  |  |  | X |  |  |  |  |  |
| Reuter et al. (2012) |  |  |  |  |  |  | X |  |  |  |  |  |  |  |  |  |
| Rojasavastera et al. (2020) |  |  |  |  |  | X |  |  |  |  |  |  |  |  |  |  |
| Santos et al. (2015) |  |  |  |  |  |  |  | X |  |  |  |  |  |  |  |  |
| Schwenk et al. (2016) |  |  |  | X |  |  |  |  |  |  | X |  |  |  |  | X |
| Shimada et al. (2018) | X |  |  |  |  |  | X |  |  |  | X | X | X | X | X |  |
| Siu & Lee (2018) |  |  |  |  |  |  |  |  | X |  |  |  |  |  |  |  |
| Straubmeier et al. (2017) |  |  |  |  |  |  | X |  |  |  |  |  |  |  |  |  |
| Styliadis et al. (2015) |  |  |  |  | X |  |  |  |  |  |  |  |  |  |  |  |
| Suzuki et al. (2012) | X |  |  |  |  |  |  |  |  | X |  |  |  |  |  |  |
| Suzuki et al. (2013) | X |  |  |  |  |  |  | X |  | X | X |  | X |  | X |  |
| Swinnen et al. (2021) |  |  |  |  |  |  |  |  |  |  |  |  |  | X |  |  |
| Tanaka et al. (2020) |  |  |  |  |  |  |  |  |  |  |  |  | X |  |  |  |
| Tarnanas et al. (2014) |  |  | X | X |  |  |  |  |  |  |  |  |  |  |  | X |
| Tarnanas et al. (2015) |  |  | X |  |  |  |  |  |  |  |  |  |  |  |  |  |
| Thapa et al. (2020) |  |  | X | X |  |  |  |  |  |  |  |  |  | X |  | X |
| Torpil et al. (2021) |  | X |  | X |  |  |  |  |  |  |  |  |  |  |  |  |
| Tsai et al. (2013) |  |  |  |  |  |  |  |  | X |  |  |  |  |  |  |  |
| Van-Santen et al. (2020) |  | X |  | X |  |  |  |  |  |  |  |  | X | X |  |  |
| Venturelli et al. (2016) | X |  |  |  |  |  |  | X |  |  |  |  | X |  | X |  |
| Xu et al. (2020) |  |  |  |  |  |  |  |  | X | X | X |  |  |  |  |  |
| Yang et al. (2022) |  |  |  |  |  |  |  |  |  | X |  |  |  |  |  |  |
| Young (2020) |  |  |  |  |  |  |  |  | X |  |  |  |  |  |  |  |
| Young et al. (2019) |  |  |  |  |  |  |  |  | X |  |  |  | X |  |  |  |
| Zhang (2019) |  |  |  |  |  |  |  |  |  |  |  |  | X |  |  |  |
| Zheng et al. (2022) |  | X |  | X |  |  |  |  |  |  |  |  |  |  |  |  |
| Total | 10 | 8 | 7 | 13 | 3 | 8 | 18 | 10 | 5 | 9 | 12 | 5 | 19 | 12 | 11 | 6 |
| % Overlap | 36/67 = 53.73% | | | | | | | | | | | | | | | |
| Covered Area (CA) | 156/16*67 = 156/1072= 0.15 | | | | | | | | | | | | | | | |
| Corrected Covered Area (CCA) | (156-67)/(1072-67) = 89/1005= 0.0886 = 8.86% = Moderate (6-10%) | | | | | | | | | | | | | | | |

# **Appendix A11.3.** Within-Studies Overlaps for HRQoL Outcomes Using PCDT

|  | Systematic reviews and meta-analyses included in the umbrella review (n = 5) | | | | |
| --- | --- | --- | --- | --- | --- |
| Primary Studies (n = 15) | Cai et al. (2023) | Gómez-Soria et al. (2022) | Hong et al. (2024) | Li et al. (2022) | Xue et al. (2023) |
| Binns et al. (2020) |  |  | X |  |  |
| Cintoli et al. (2021) |  |  | X |  |  |
| Hagovská & Olekszyová (2016) |  | X |  |  |  |
| Hagovska et al. (2016) |  |  | X |  |  |
| Jiao et al. (2022) |  |  |  |  | X |
| Maci et al. (2012) |  |  | X |  |  |
| Maffei et al. (2017) |  |  | X |  |  |
| Muñiz et al. (2015) |  |  | X |  |  |
| Padala et al. (2012) | X |  |  |  |  |
| Padala et al. (2017) | X |  |  |  |  |
| Tanaka et al. (2020) |  |  |  |  | X |
| Van-Santen et al. (2020) | X |  |  |  | X |
| Xu et al. (2020) |  |  |  | X |  |
| Young (2020) |  |  |  | X | X |
| Zheng et al. (2022) | X |  |  |  |  |
| Total | 4 | 1 | 6 | 2 | 4 |
| % Overlap | 2/14 = 14.29% | | | | |
| Covered Area (CA) | 17/5*15 = 17/75 = 0.23 | | | | |
| Corrected Covered Area (CCA) | (17-15)/(75-15) = 2/60 = 0.0333 = 3.33% = Slight (0-5%) | | | | |

# **Appendix A11.4.** Within-Studies Overlaps for ADL Outcomes Using PCDT

|  | Systematic reviews and meta-analyses included in the umbrella review (n = 6) | | | | | |
| --- | --- | --- | --- | --- | --- | --- |
| Primary Studies (n = 17) | Cai et al. (2023) | Gómez-Soria et al. (2022) | Hong et al. (2024) | Karssemeijer et al. (2017) | Xue et al. (2023) | Ye et al. (2024) |
| Fiatarone et al. (2014) |  |  | X | X | X |  |
| Graessel et al. (2011) |  |  |  | X |  |  |
| Hagovska & Nagyova (2017) |  |  |  |  | X |  |
| Hagovska et al. (2016) |  |  | X |  |  | X |
| Han et al. (2017) |  |  |  | X |  |  |
| Holthoff et al. (2015) |  |  |  | X |  |  |
| Jiao et al. (2022) |  |  |  |  | X |  |
| Karssemeijer et al. (2019b) |  |  | X |  |  |  |
| Kounti et al. (2011) |  | X |  |  |  |  |
| Luttenberger et al. (2015) |  |  | X |  |  |  |
| Menengi et al. (2022) |  |  |  |  |  | X |
| Mrakic-Sposta et al. (2018) |  |  |  |  | X |  |
| Okamura et al. (2018) |  |  | X |  | X |  |
| Padala et al. (2012) | X |  |  |  |  |  |
| Padala et al. (2017) | X |  |  |  |  |  |
| Swinnen et al. (2021) |  |  |  |  |  | X |
| Tanaka et al. (2020) |  |  |  |  | X |  |
| Total | 2 | 1 | 5 | 4 | 6 | 3 |
| % Overlap | 3/17 = 17.65% | | | | | |
| Covered Area (CA) | 21/6*17 = 21/102 = 0.21 | | | | | |
| Corrected Covered Area (CCA) | (21-17)/(102-17) = 4/85= 0.0471 = 4.71% = Slight (0-5%) | | | | | |

# **Appendix A11.5.** Within-Studies Overlaps for Gait Outcomes Using PCDT

|  | Systematic reviews and meta-analyses included in the umbrella review (n = 4) | | | |
| --- | --- | --- | --- | --- |
| Primary Studies (n = 19) | Cai et al. (2023) | Hong et al. (2024) | Ye et al. (2024) | Zhu et al. (2021) |
| Bischoff et al. (2021) |  |  | X |  |
| Callisaya et al. (2021) |  |  | X |  |
| Combourieu-Donnezan et al. (2018) |  |  | X |  |
| Delbroek et al. (2017) |  | X |  | X |
| Hagovska et al. (2016) |  | X |  |  |
| Hughes et al. (2014) |  |  |  | X |
| Karssemeijer et al. (2019a) | X |  |  |  |
| Karssemeijer et al. (2019b) |  | X |  |  |
| Kuo et al. (2022) |  |  | X |  |
| Lemke et al. (2019) |  |  | X |  |
| Liao et al. (2019) |  |  |  | X |
| Liu et al. (2022) | X |  |  |  |
| Nam & Kim (2021) |  |  | X |  |
| Padala et al. (2012) | X |  |  | X |
| Park et al. (2019) |  |  | X |  |
| Schwenk et al. (2010) |  |  | X |  |
| Schwenk et al. (2016) |  |  |  | X |
| Shimada et al. (2018) |  |  | X |  |
| Thapa et al. (2020) |  |  | X | X |
| Total | 3 | 3 | 10 | 6 |
| % Overlap | 3/19 = 0.1578 = 15.79% | | | |
| Covered Area (CA) | 22/4*19 = 22/76 = 0.29 | | | |
| Corrected Covered Area (CCA) | (22-19)/(76-19) = 3/57 = 0.0526 = 5.26% = Slight(0-5%) | | | |

# **Appendix A11.6.** Within-Studies Overlaps for Balance Outcomes Using PCDT

|  | Systematic reviews and meta-analyses included in the umbrella review (n = 5) | | | | |
| --- | --- | --- | --- | --- | --- |
| Primary Studies (n = 16) | Ali et al. (2022) | Cai et al. (2023) | Wati et al. (2024) | Ye et al. (2024) | Zhu et al. (2021) |
| Binns et al. (2020) |  |  | X |  |  |
| Callisaya et al. (2021) |  |  |  | X |  |
| Chen et al. (2018) | X |  |  |  |  |
| Combourieu-Donnezan et al. (2018) | X |  | X | X |  |
| Delbroek et al. (2017) | X |  |  | X | X |
| Hagovská & Olekszyová (2016) |  |  | X |  |  |
| Hagovska et al. (2016) |  |  |  | X |  |
| Hwang & Lee (2017) |  |  |  |  | X |
| Makizako et al. (2012) | X |  |  |  |  |
| Menengi et al. (2022) |  |  |  | X |  |
| Nam & Kim (2021) |  |  |  | X |  |
| Padala et al. (2012) |  | X |  |  | X |
| Padala et al. (2017) |  | X |  |  |  |
| Park et al. (2019) | X |  |  | X |  |
| Schwenk et al. (2016) |  |  |  |  | X |
| Thapa et al. (2020) |  |  |  | X |  |
| Total | 5 | 2 | 3 | 8 | 4 |
| % Overlap | 4/16 = 0.25 = 25.00% | | | | |
| Covered Area (CA) | 22/4*16 = 22/64 = 0.34 | | | | |
| Corrected Covered Area (CCA) | (22-16)/(64-16) = 6/48 = 0.1250 = 12.50% = High (11-15%) | | | | |

##

# **Appendix A12.** Mixed-Effects Meta-Regression of Age on Outcomes

| Outcomes | Covariate | Meta-regression | | | |
| --- | --- | --- | --- | --- | --- |
|  |  | *β* | SE | 95% CI | *p-*value |
| Global cognition | Age | -0.05 | 0.03 | -0.10, 0.001 | 0.06 |
| HRQoL | Age | -0.02 | 0.04 | -0.10, 0.06 | 0.56 |
| ADL | Age | -0.02 | 0.05 | -0.11, 0.07 | 0.65 |
| Gait | Age | -0.01 | 0.06 | -0.13, 0.10 | 0.80 |
| Balance | Age | -0.02 | 0.04 | -0.09, 0.05 | 0.60 |

*Note. β:* Coefficient; ADL: Activities of daily living; CI: Confident interval; HRQoL: Health-related quality of life; SE: Standard error

# **Appendix B1.** Cognitive Decline Trajectory from Mild Cognitive Impairment to Dementia

**
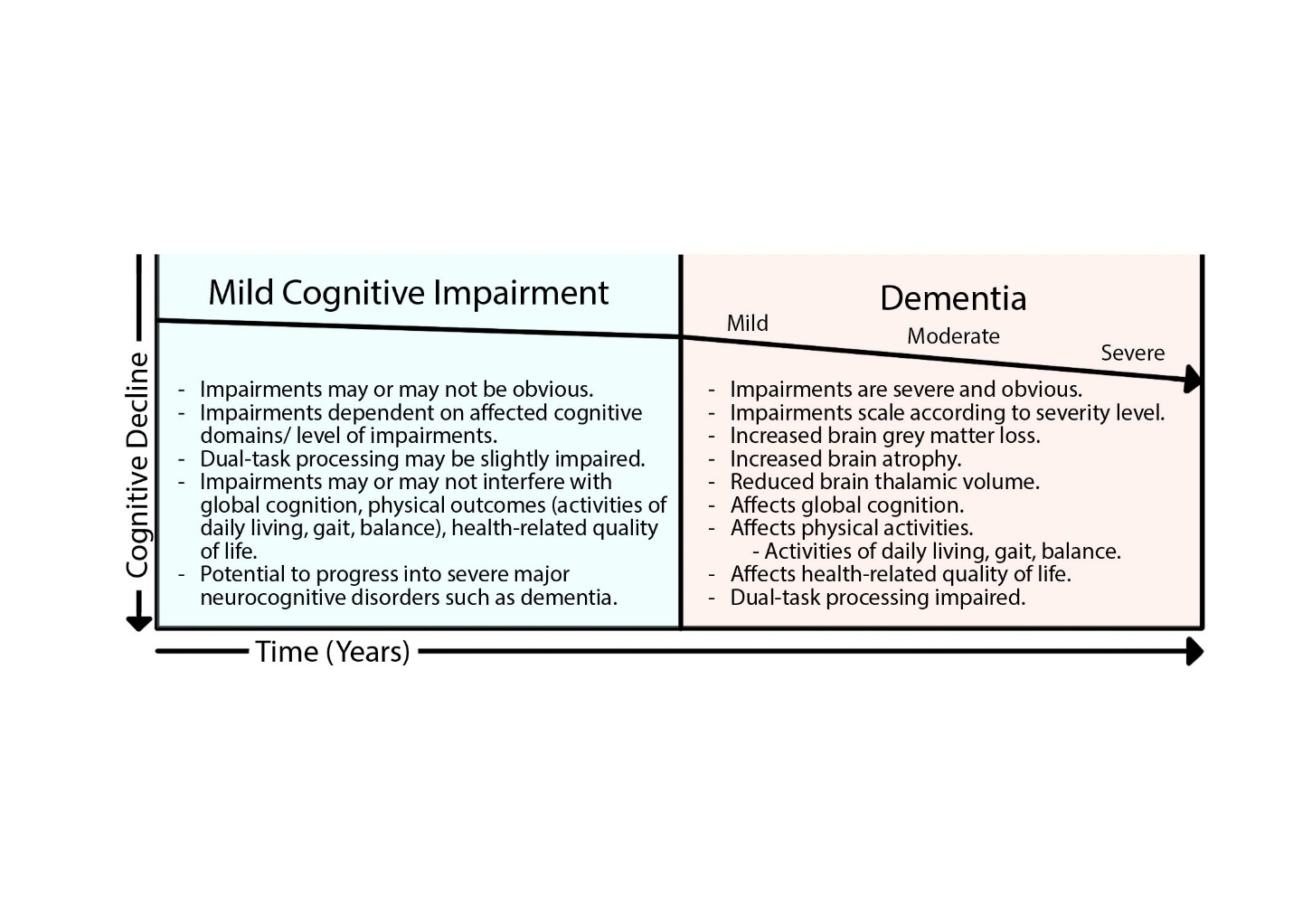
**

# **Appendix B2.1.** Forest Plot of Effect Sizes (Hedges’ g) of Meta-Level Data for Global Cognition


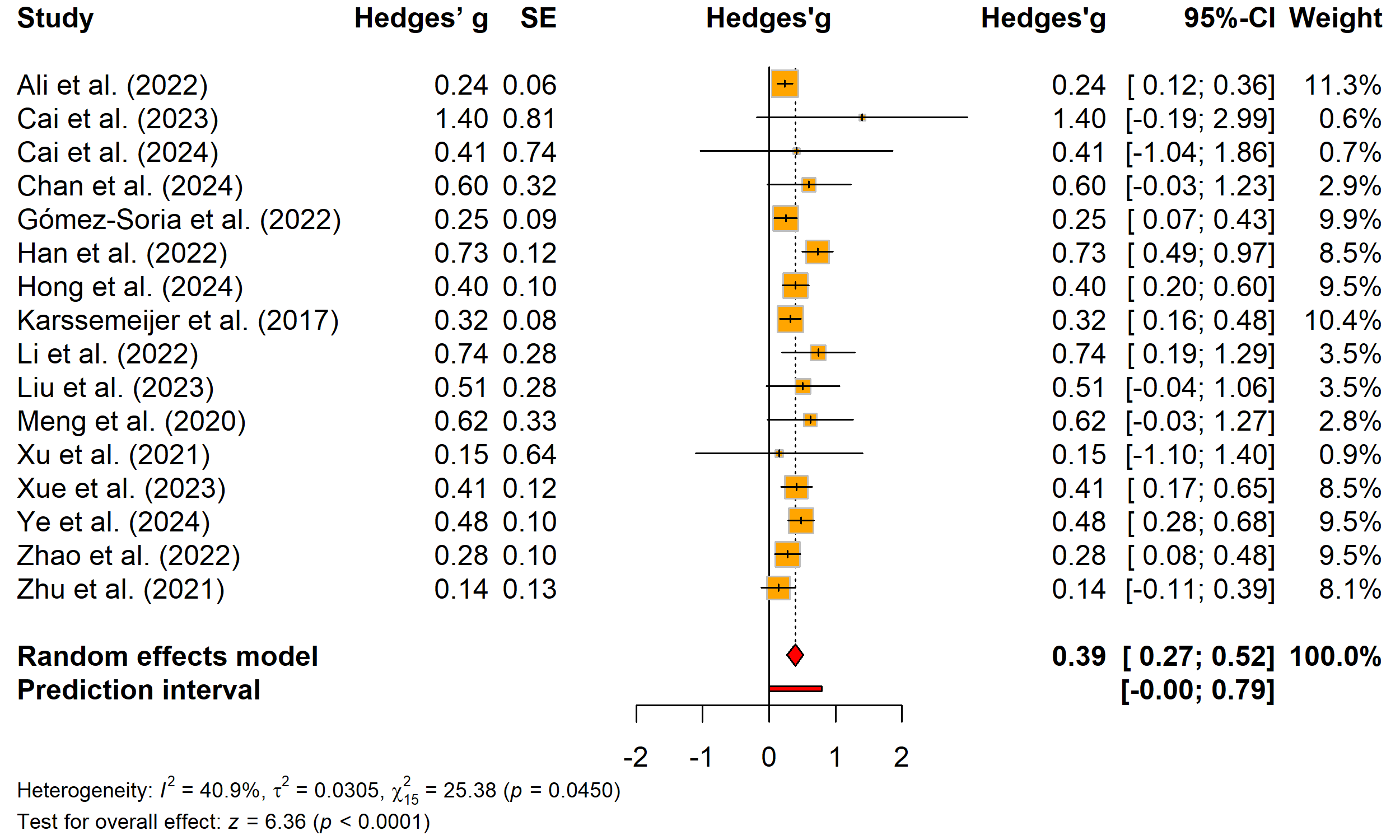


# **Appendix B2.2.** Forest Plot of Effect Sizes (Hedges’ g) of Study-Level Data for Global Cognition


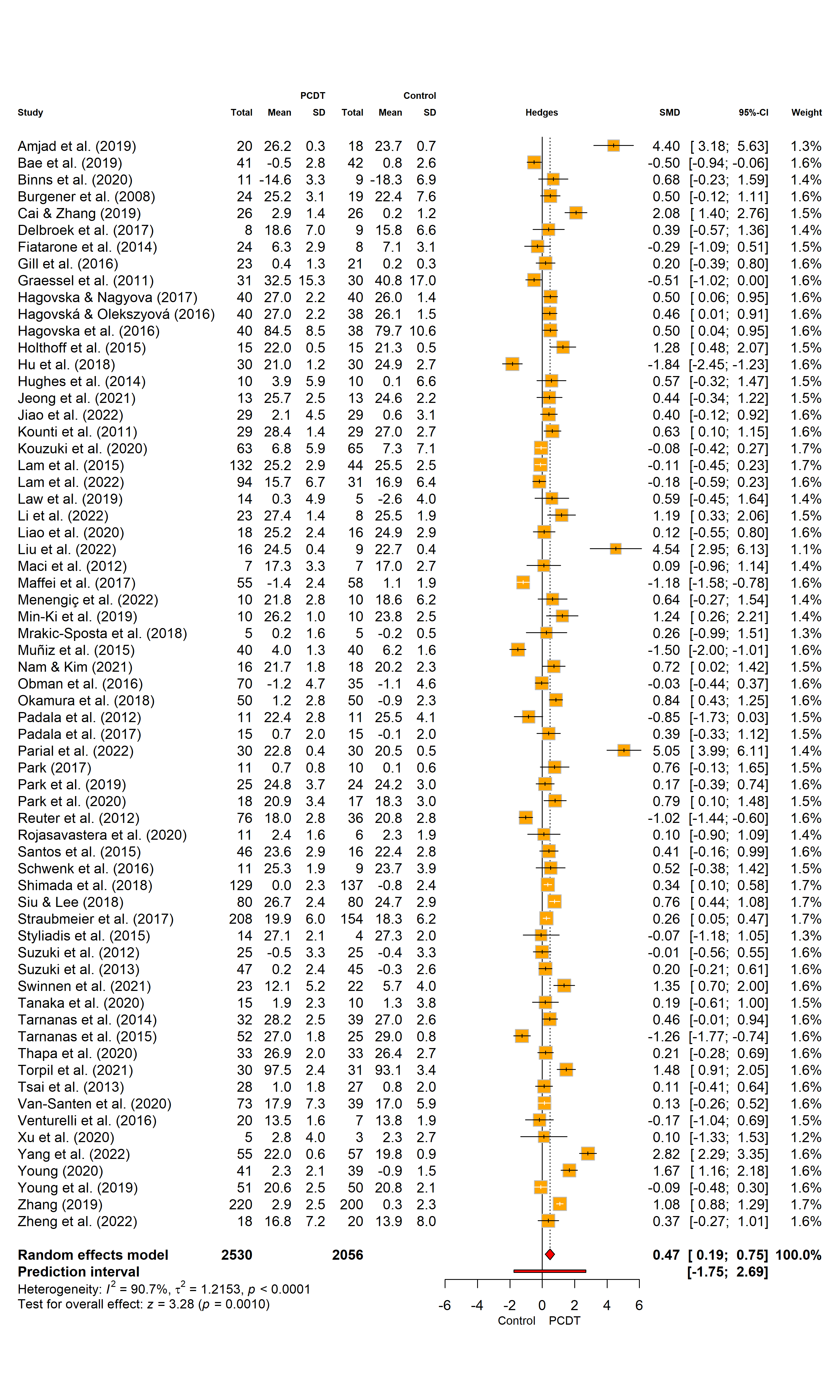


# **Appendix B2.3.** Subgroup Analyses of Forest Plot of Effect Sizes (Hedges’ g) of Study-Level Data for NCD Nature for Global Cognition


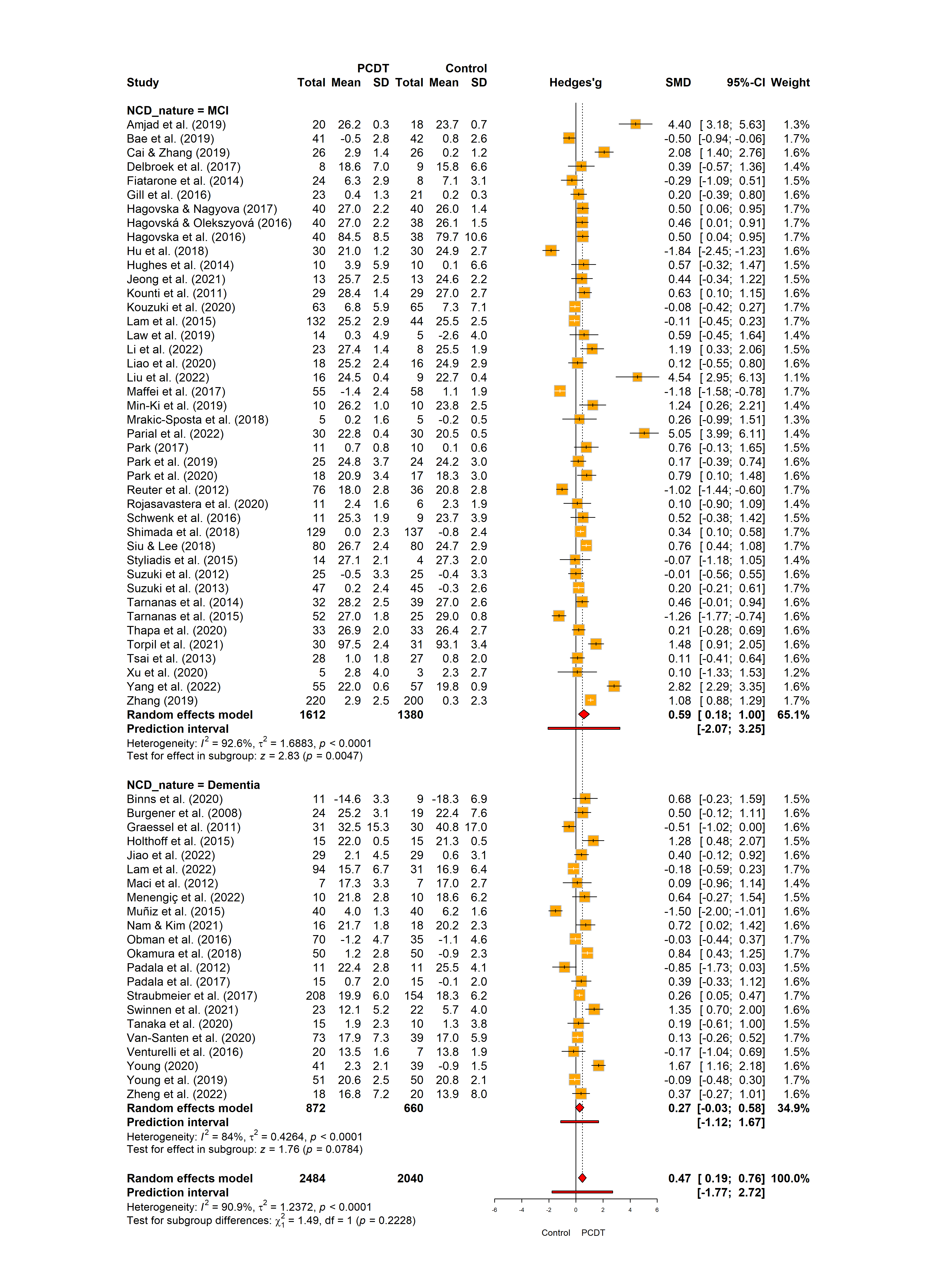


# **Appendix B2.4.** Subgroup Analyses of Forest Plot of Effect Sizes (Hedges’ g) of Study-Level Data for Intervention Type for Global Cognition


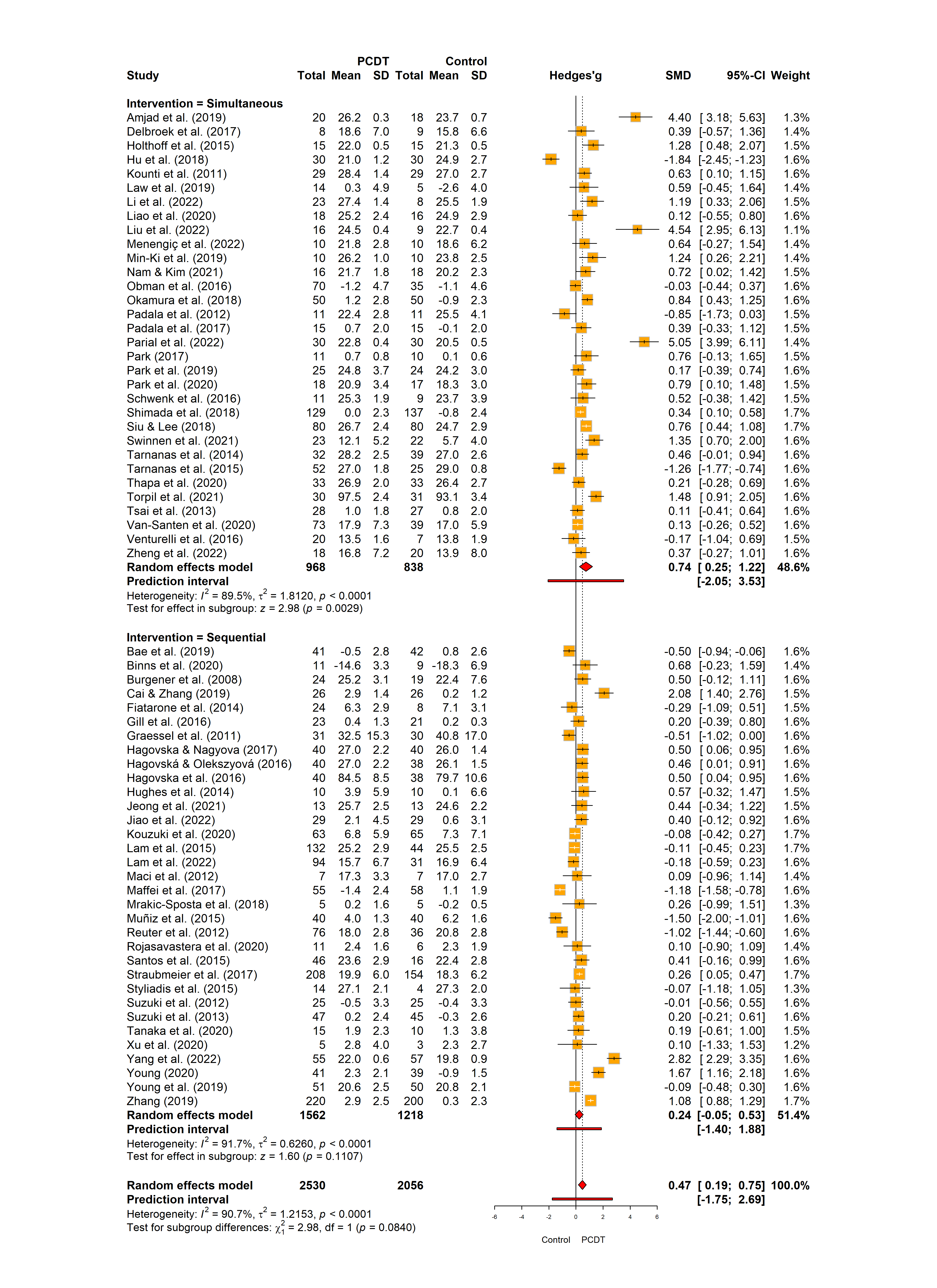


# **Appendix B2.5.** Subgroup Analyses of Forest Plot of Effect Sizes (Hedges’ g) of Study-Level Data for Training Duration for Global Cognition


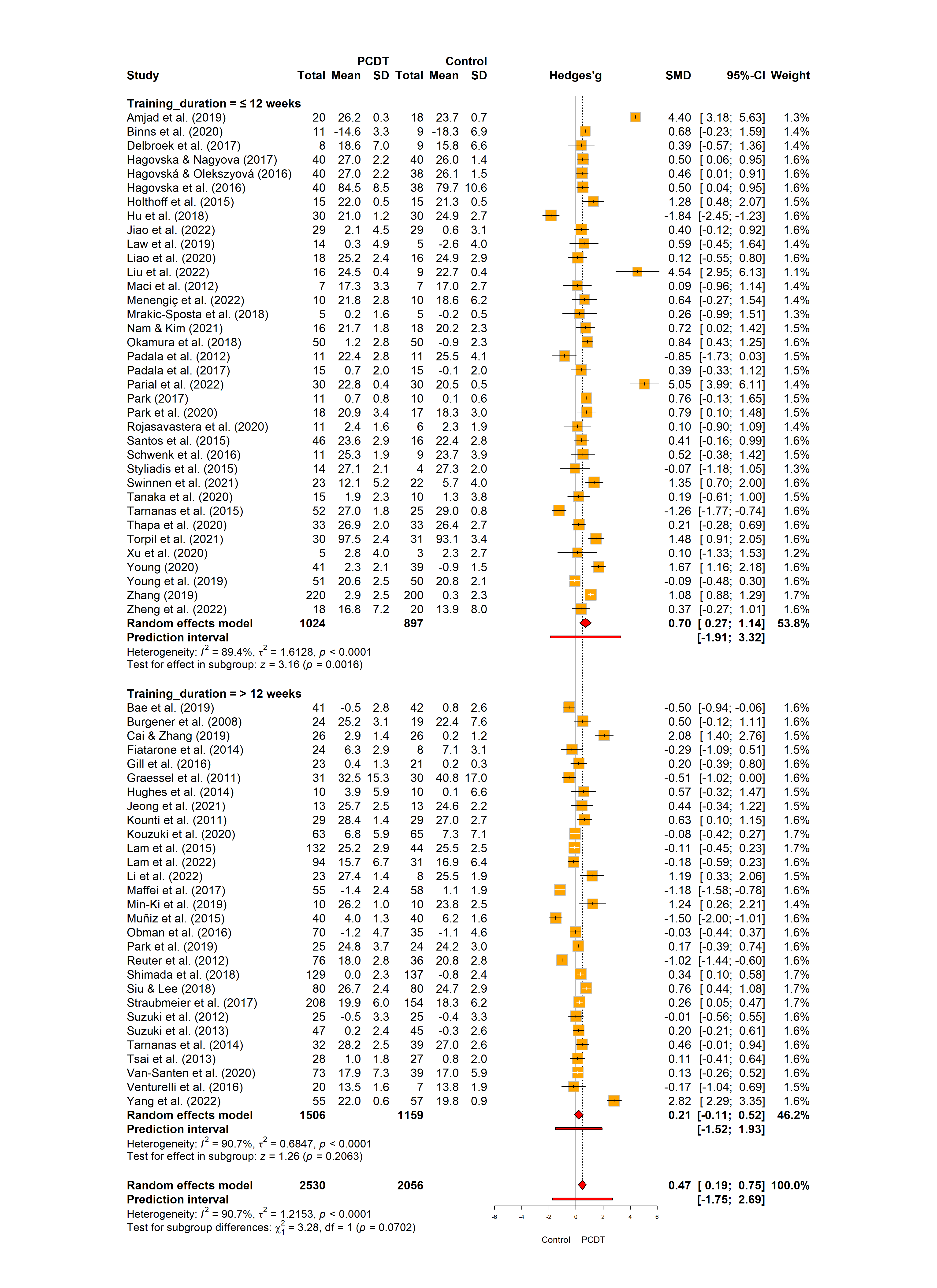


# **Appendix B2.6.** Subgroup Analyses of Forest Plot of Effect Sizes (Hedges’ g) of Study-Level Data for Session Duration for Global Cognition


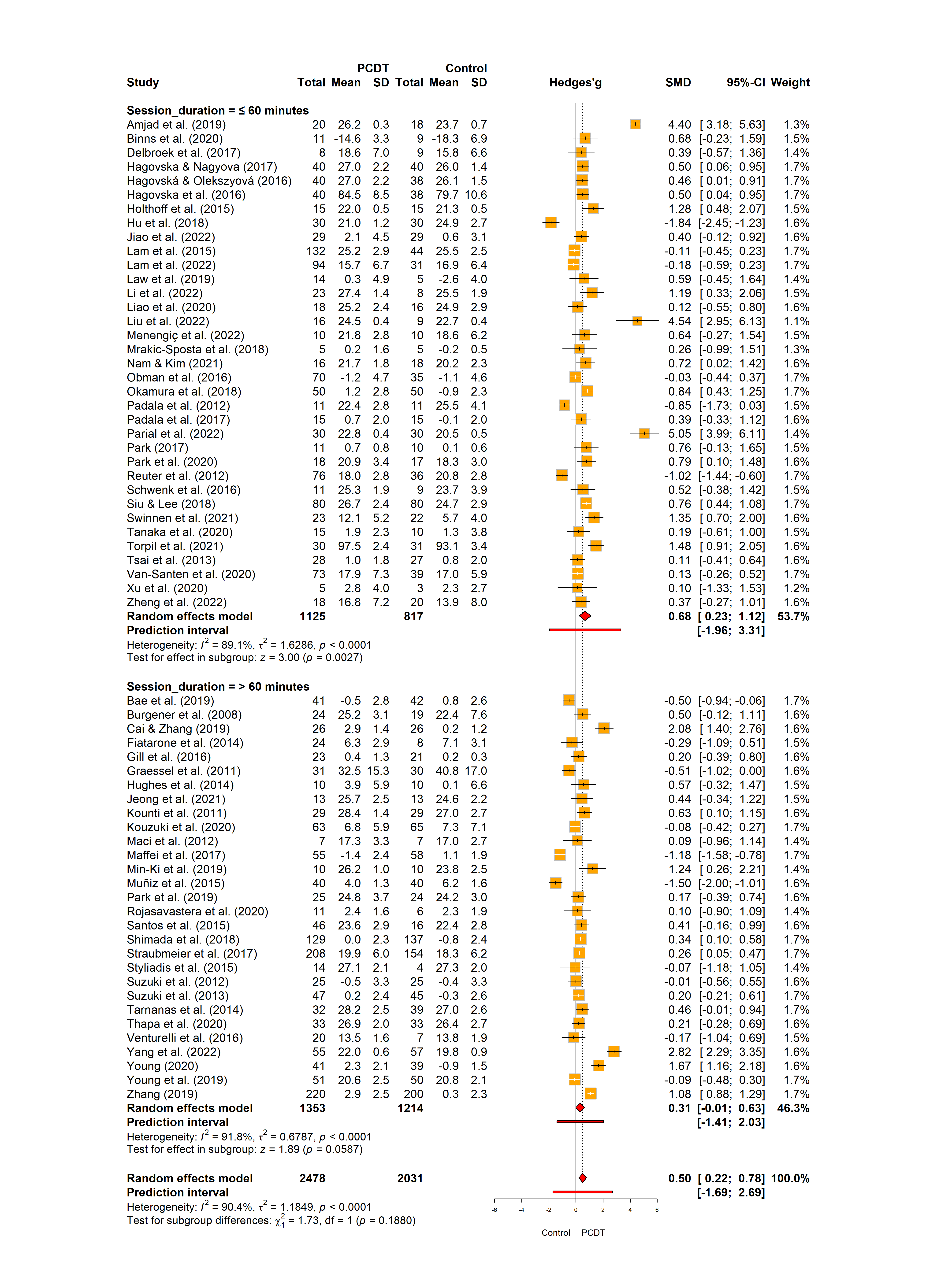


# **Appendix B2.7.** Subgroup Analyses of Forest Plot of Effect Sizes (Hedges’ g) of Study-Level Data for Training Frequency for Global Cognition


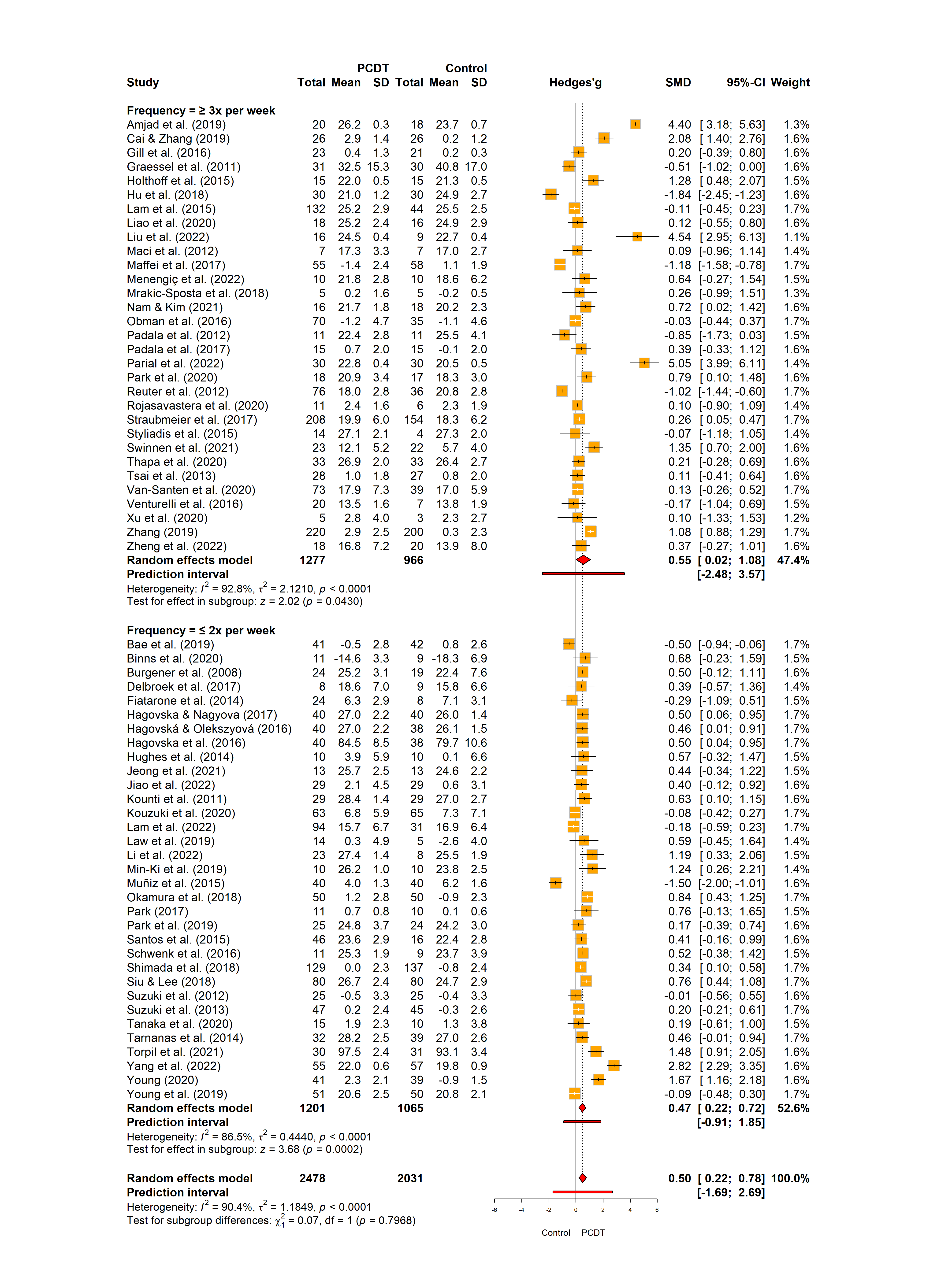


# **Appendix B3.1.** Forest Plot of Effect Sizes (Hedges’ g) of Meta-Level Data for HRQoL


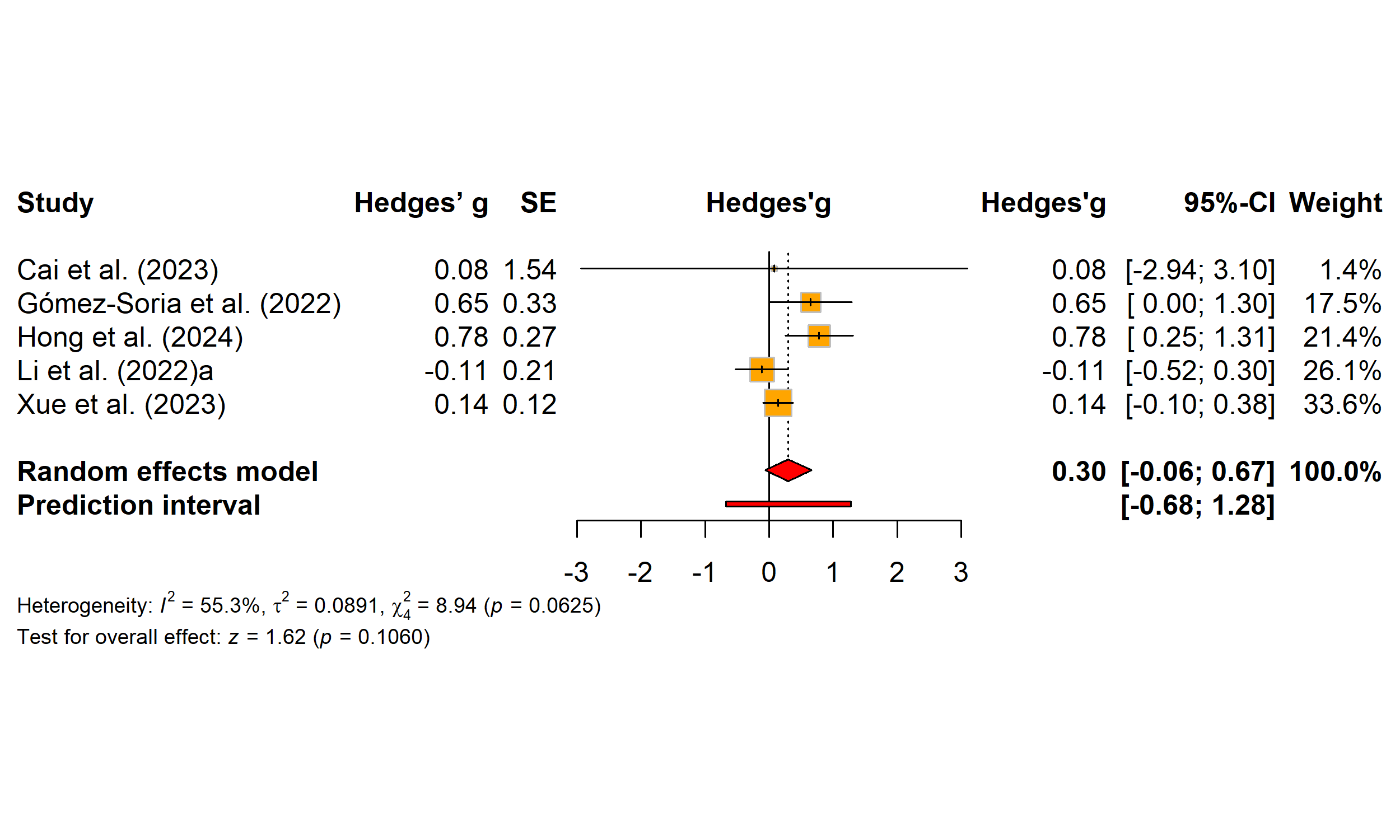


# **Appendix B3.2.** Forest Plot of Effect Sizes (Hedges’ g) of Study-Level Data for HRQoL


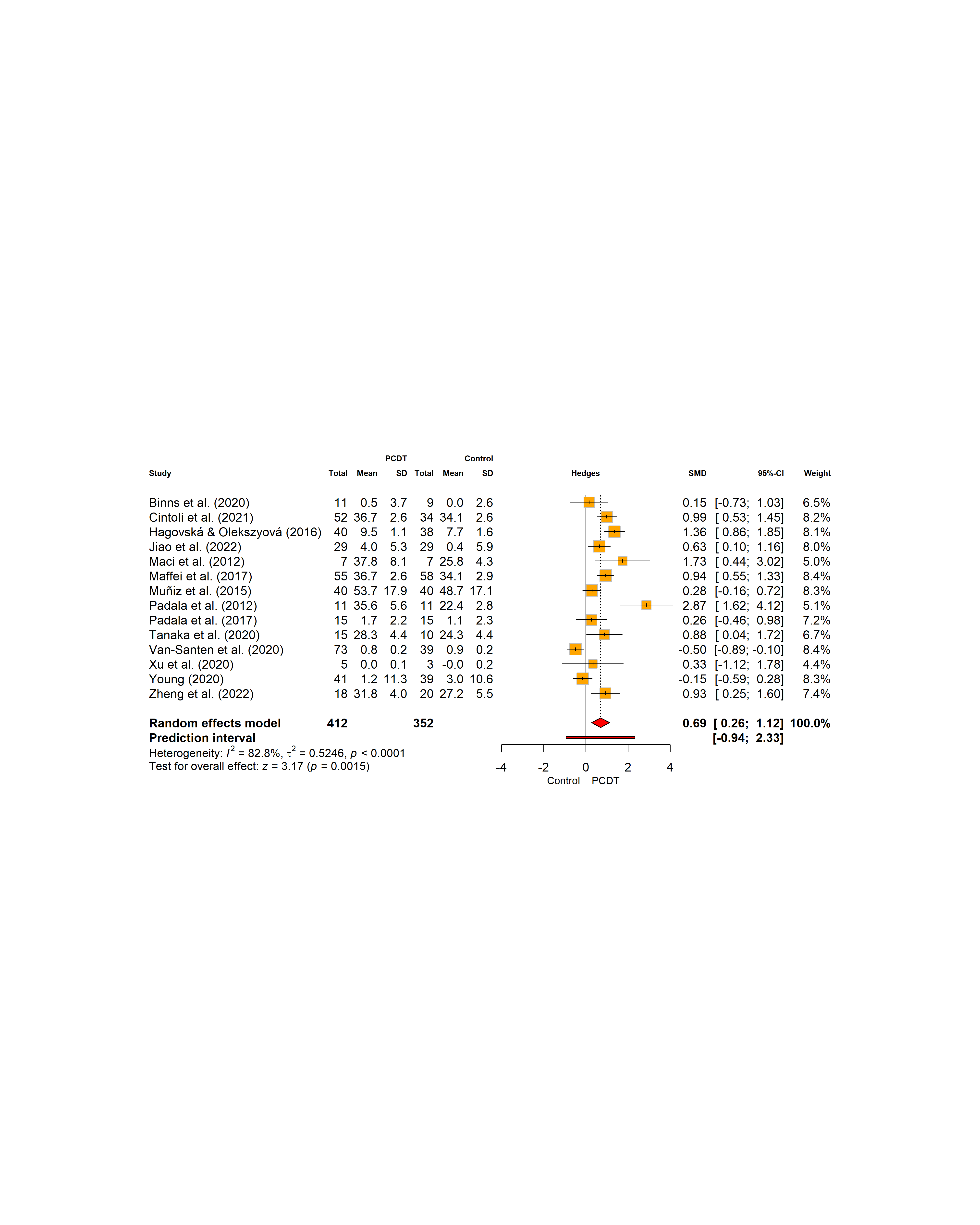


# **Appendix B3.3.** Subgroup Analyses of Forest Plot of Effect Sizes (Hedges’ g) of Study-Level Data for NCD Type for HRQoL


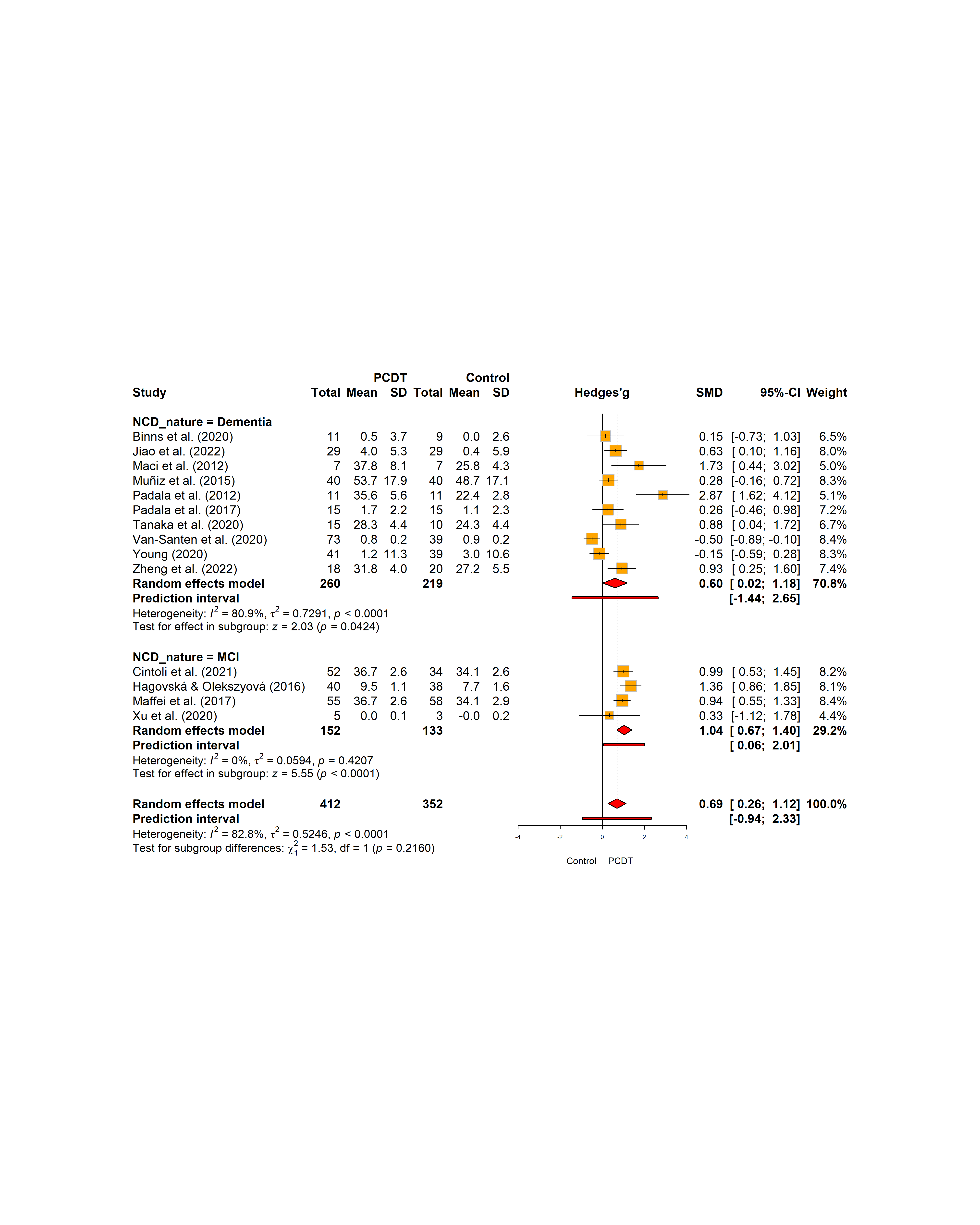


# **Appendix B3.4.** Subgroup Analyses of Forest Plot of Effect Sizes (Hedges’ g) of Study-Level Data for Intervention Type for HRQoL


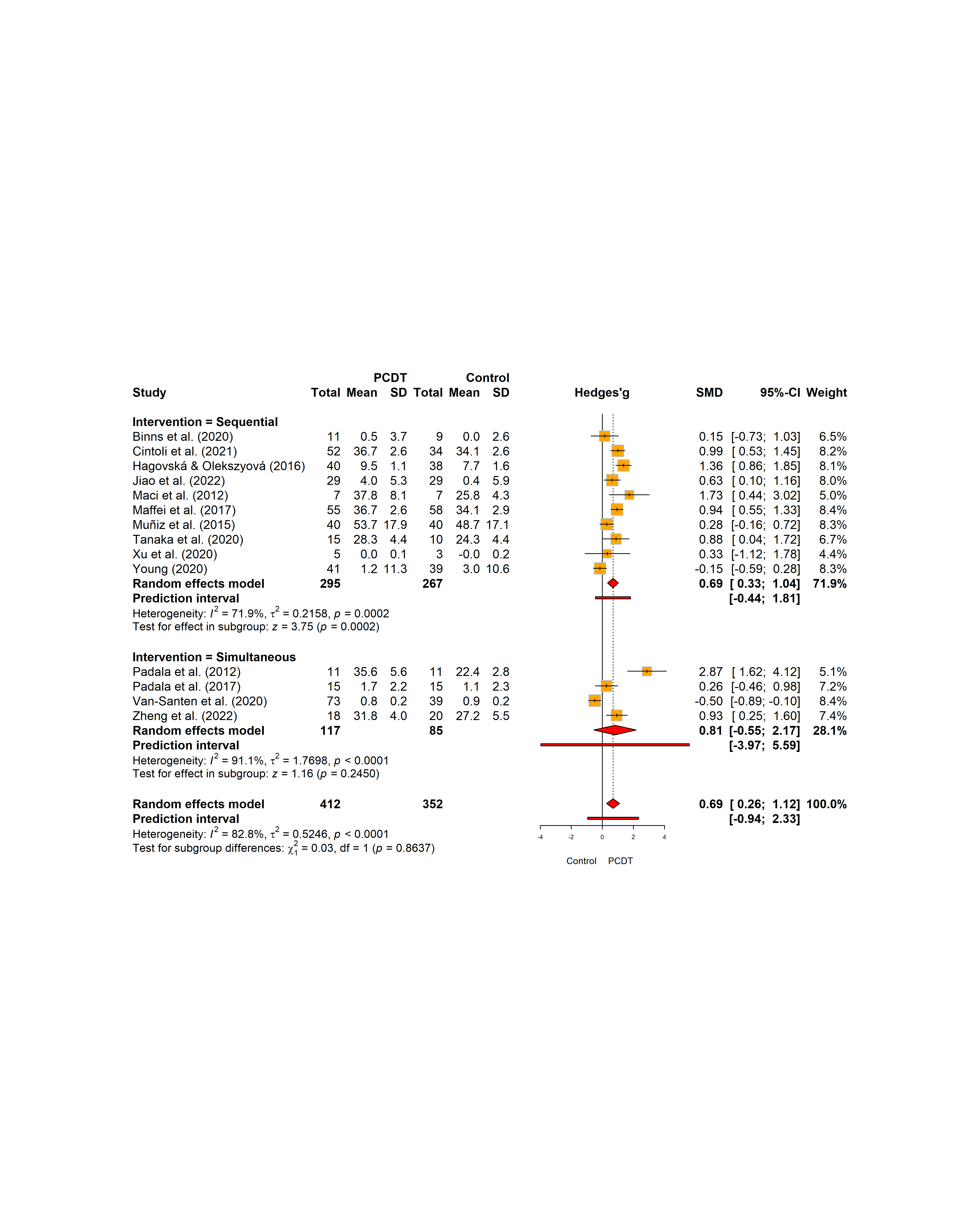


# **Appendix B3.5.** Subgroup Analyses of Forest Plot of Effect Sizes (Hedges’ g) of Study-Level Data for Training Duration for HRQoL


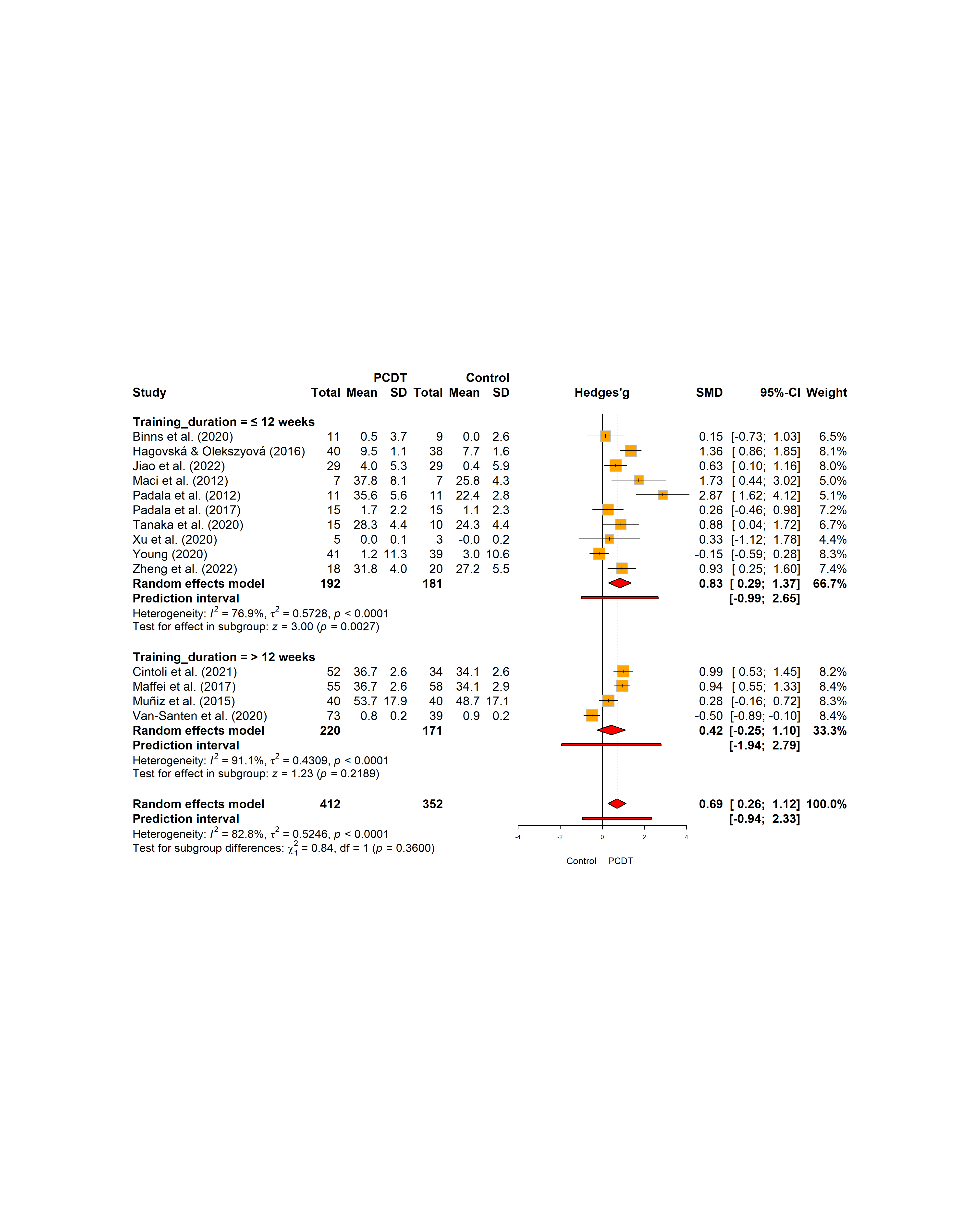


# **Appendix B3.6.** Subgroup Analyses of Forest Plot of Effect Sizes (Hedges’ g) of Study-Level Data for Session Duration for HRQoL


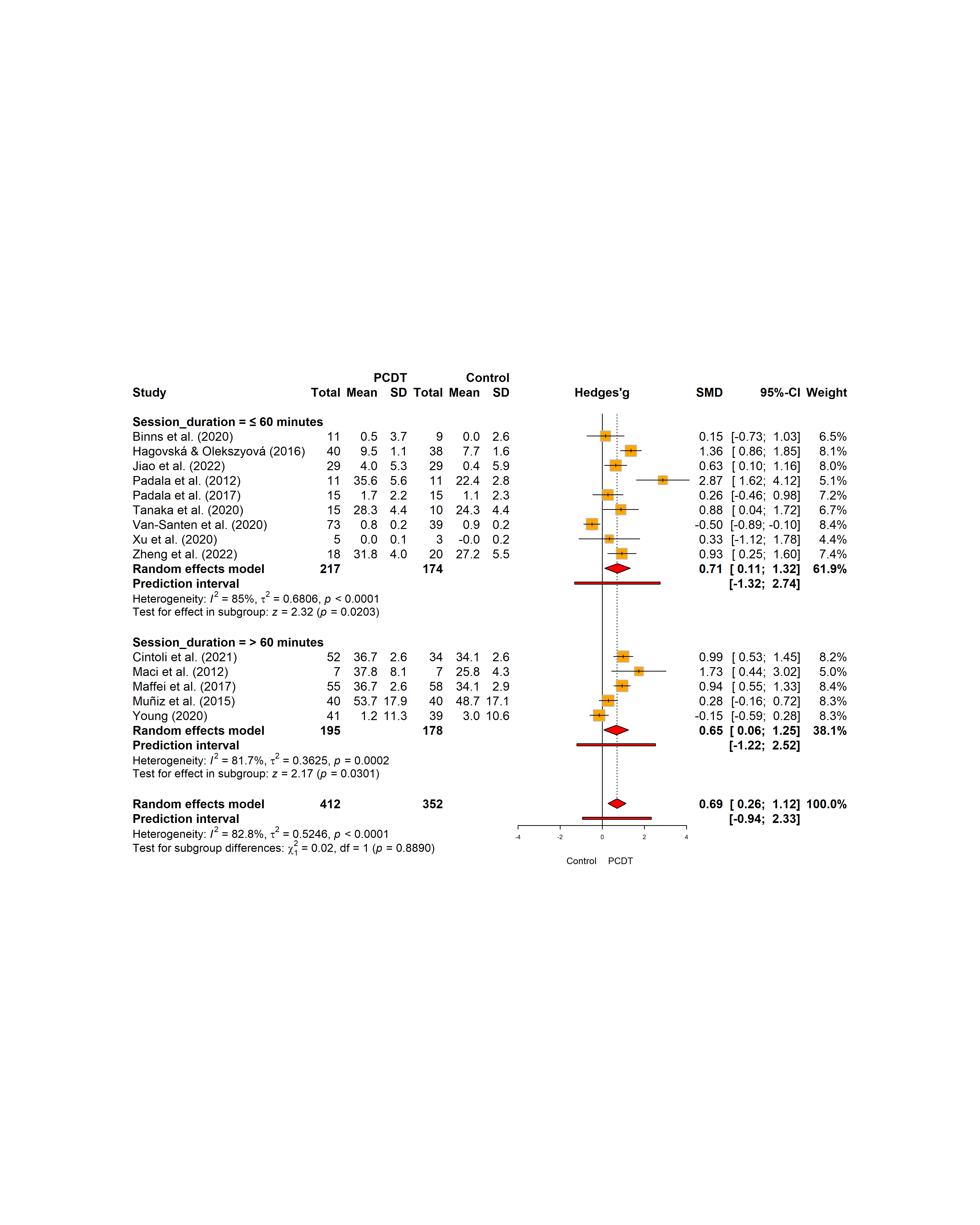


# **Appendix B3.7.** Subgroup Analyses of Forest Plot of Effect Sizes (Hedges’ g) of Study-Level Data for Training Frequency for HRQoL


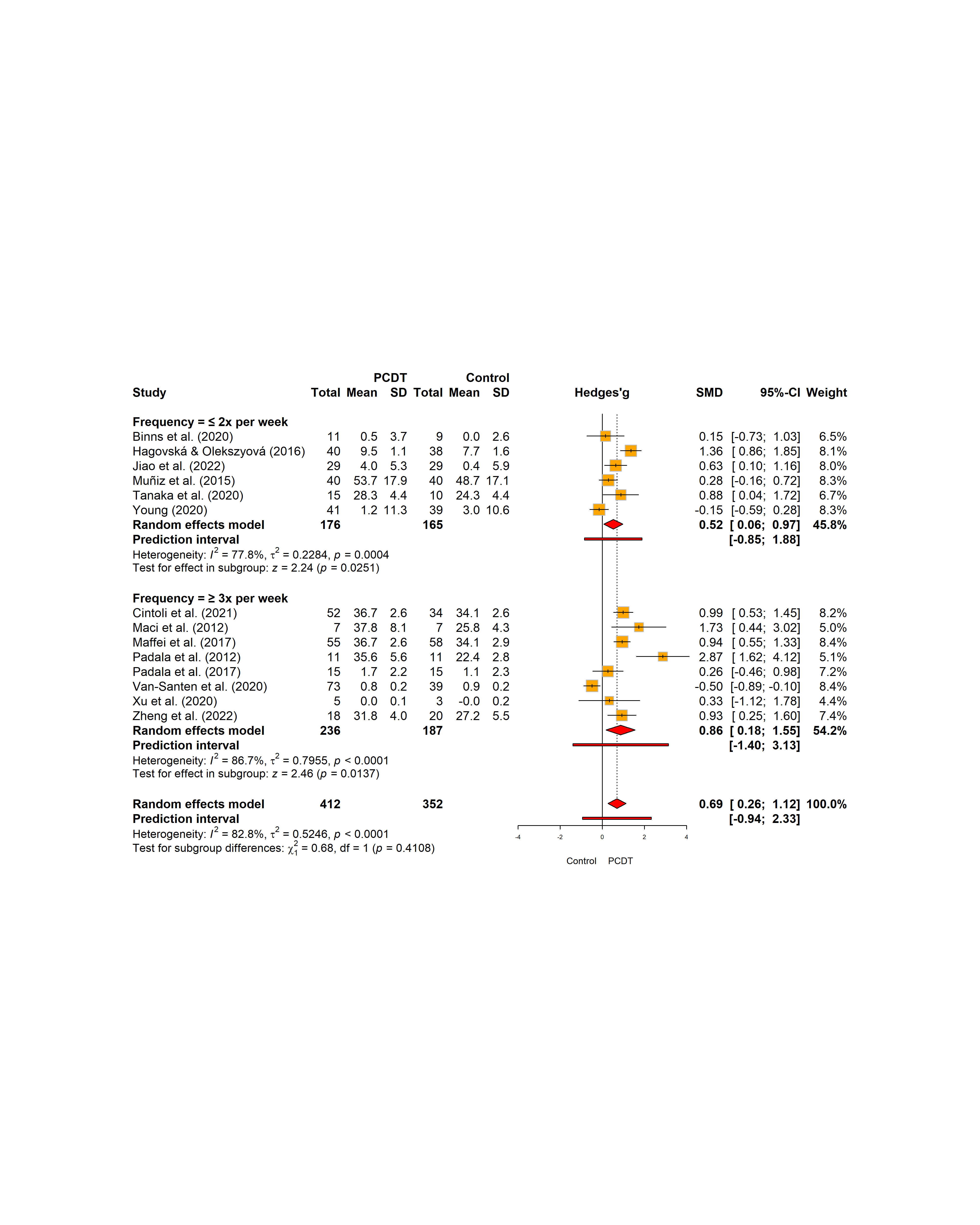


# **Appendix B4.1.** Forest Plot of Effect Sizes (Hedges’ g) of Meta-Level Data for ADL


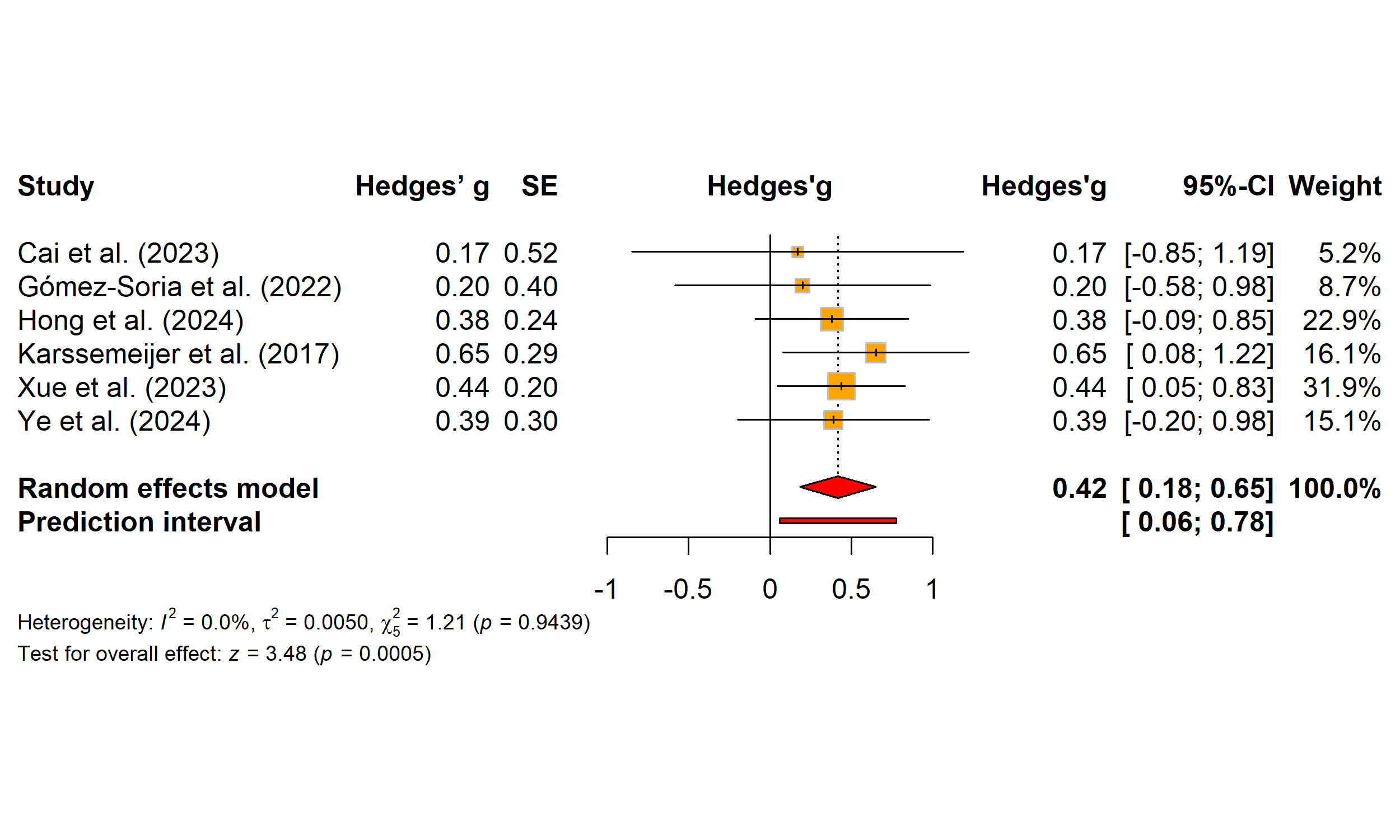


# **Appendix B4.2.** Forest Plot of Effect Sizes (Hedges’ g) of Study-Level Data for ADL


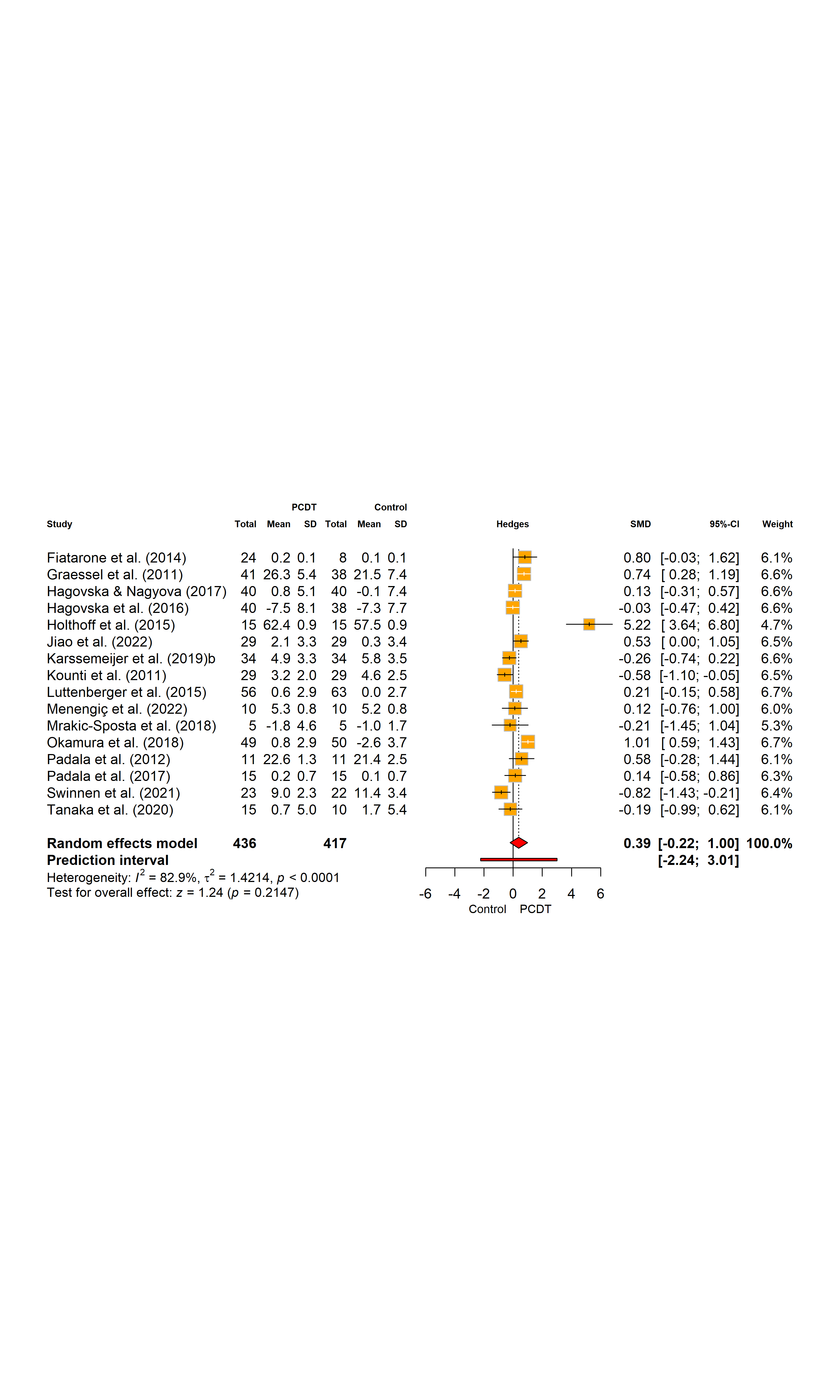


# **Appendix B4.3.** Subgroup Analyses of Forest Plot of Effect Sizes (Hedges’ g) of Study-Level Data for NCD Nature for ADL


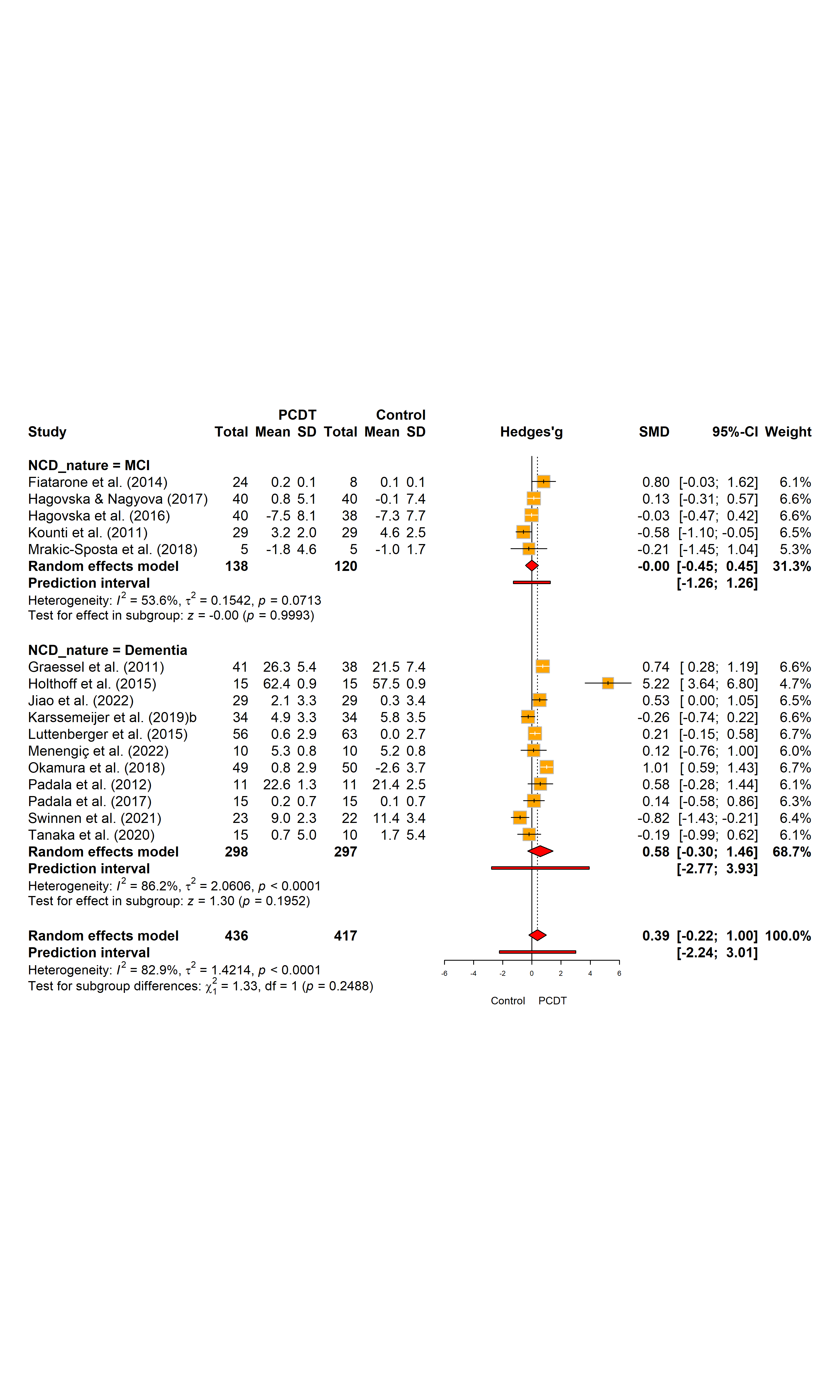


# **Appendix B4.4.** Subgroup Analyses of Forest Plot of Effect Sizes (Hedges’ g) of Study-Level Data for Intervention Type for ADL


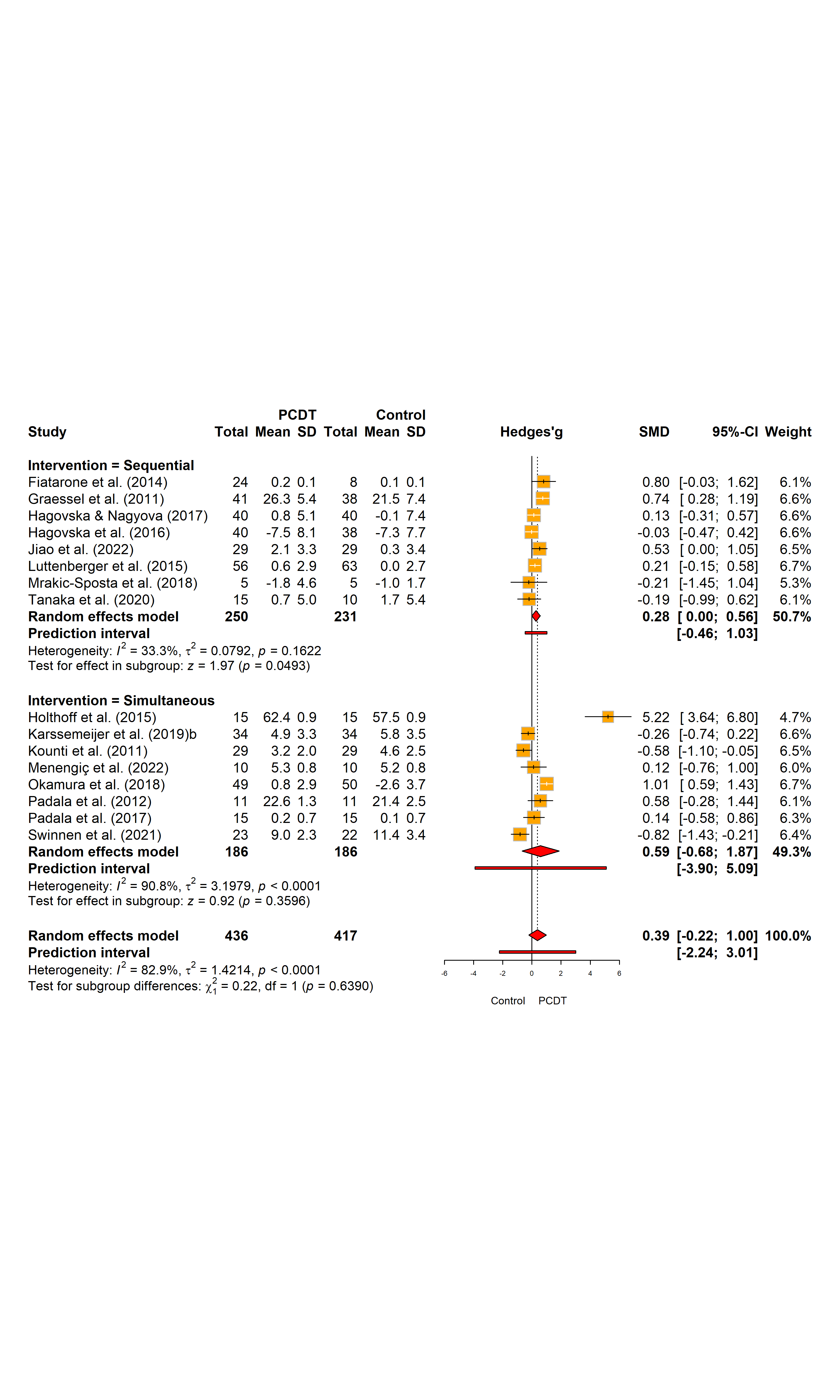


# **Appendix B4.5.** Subgroup Analyses of Forest Plot of Effect Sizes (Hedges’ g) of Study-Level Data for Training Duration for ADL


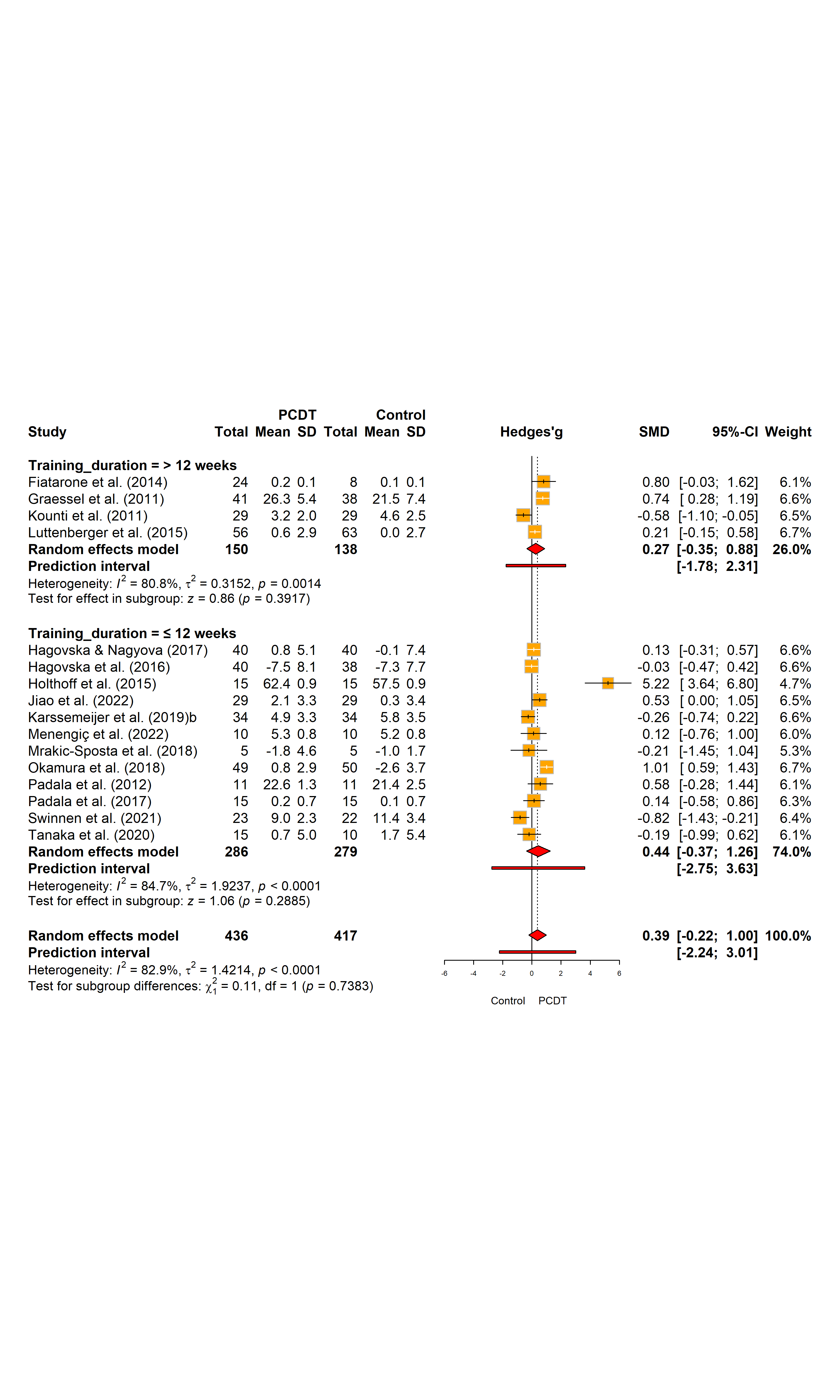


# **Appendix B4.6.** Subgroup Analyses of Forest Plot of Effect Sizes (Hedges’ g) of Study-Level Data for Session Duration for ADL


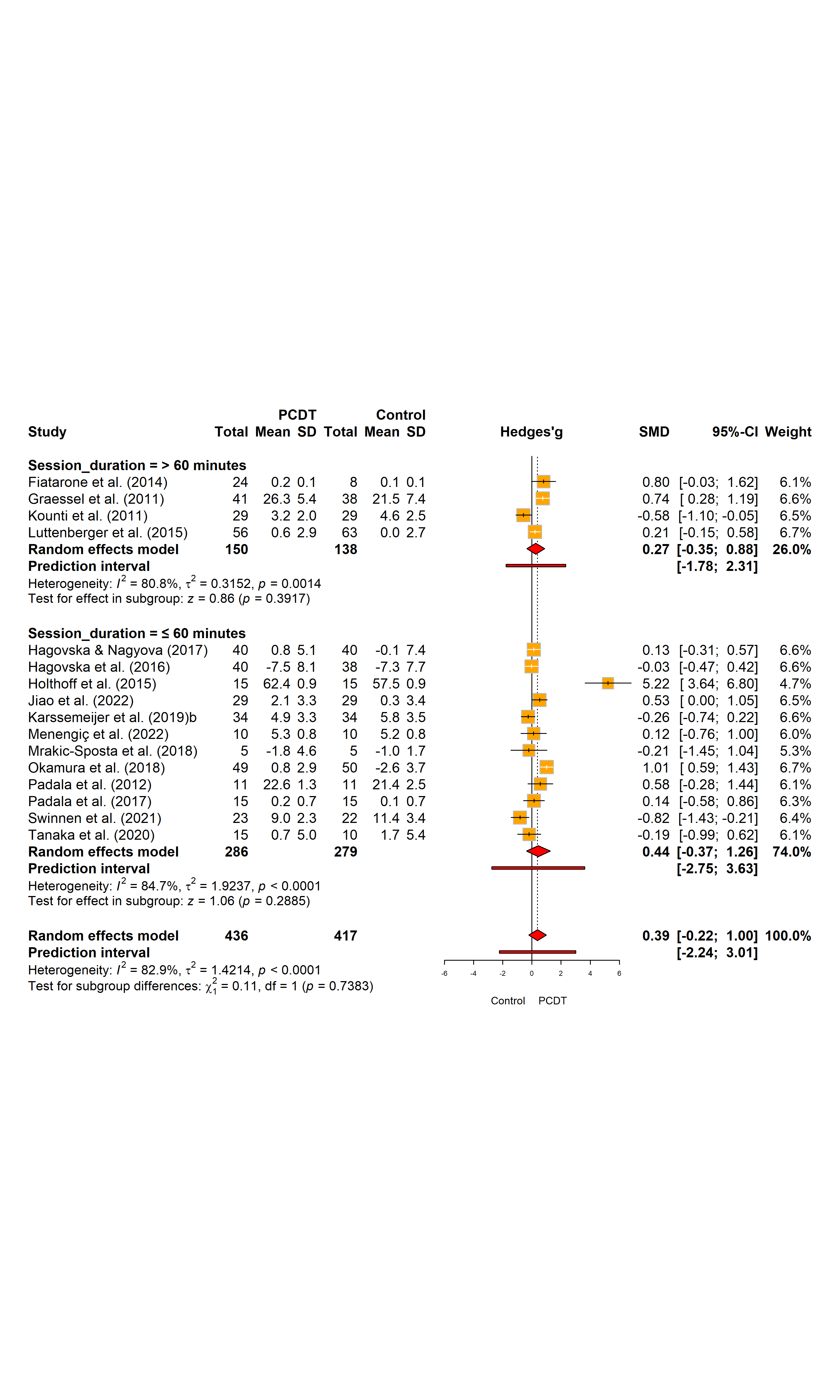


# **Appendix B4.7.** Subgroup Analyses of Forest Plot of Effect Sizes (Hedges’ g) of Study-Level Data for Training Frequency for ADL


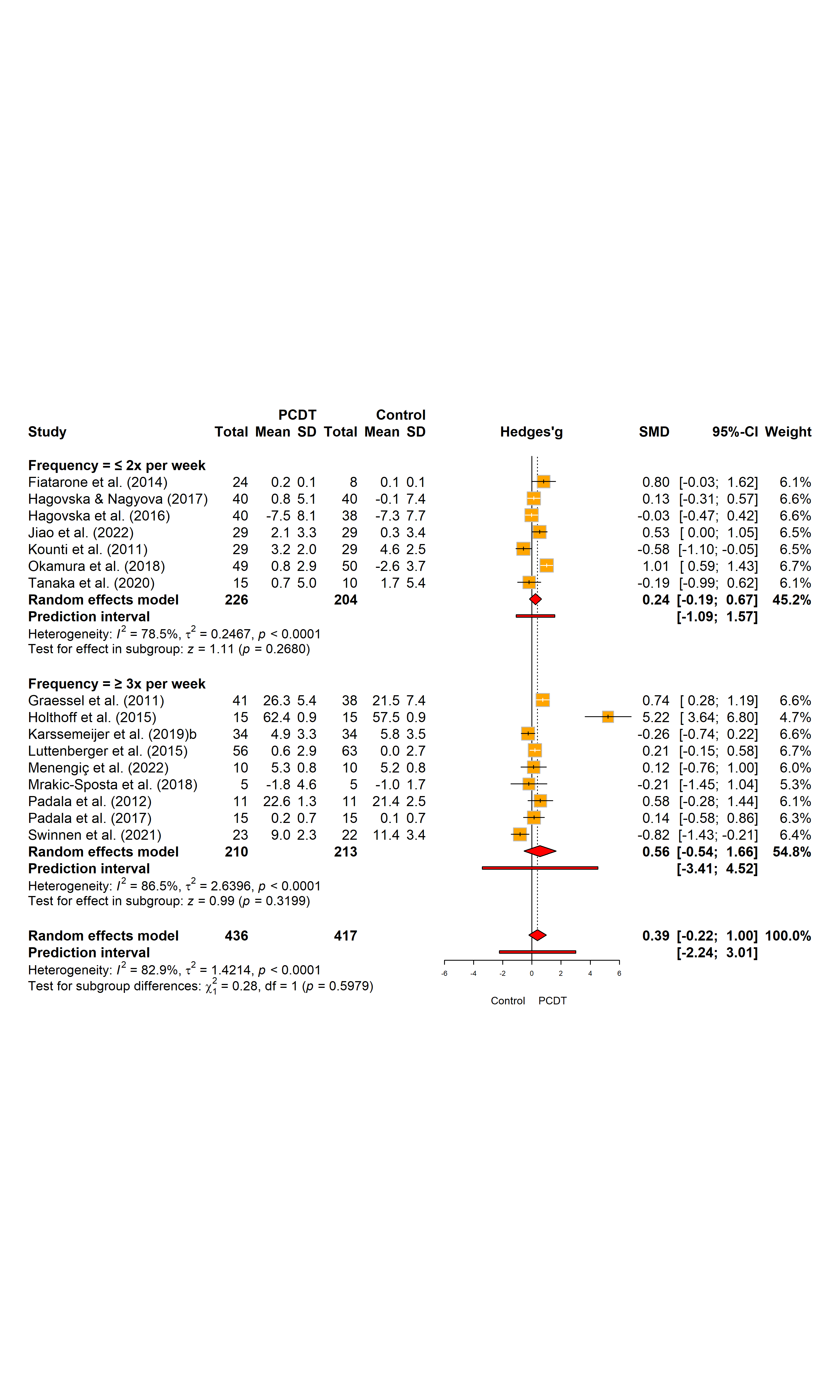


# **Appendix B5.1.** Forest Plot of Effect Sizes (Hedges’ g) of Meta-Level Data for Gait


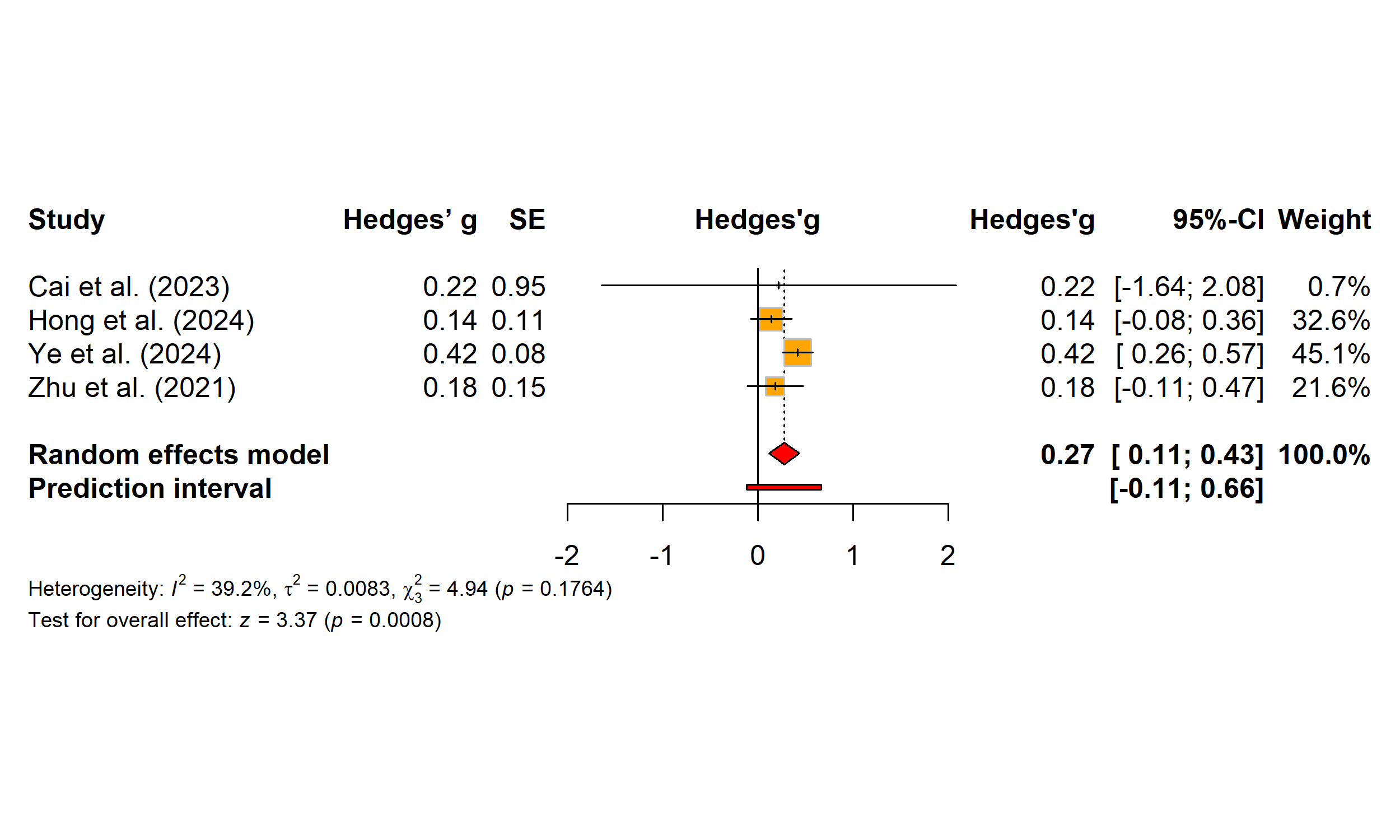


# **Appendix B5.2.** Forest Plot of Effect Sizes (Hedges’ g) of Study-Level Data for Gait


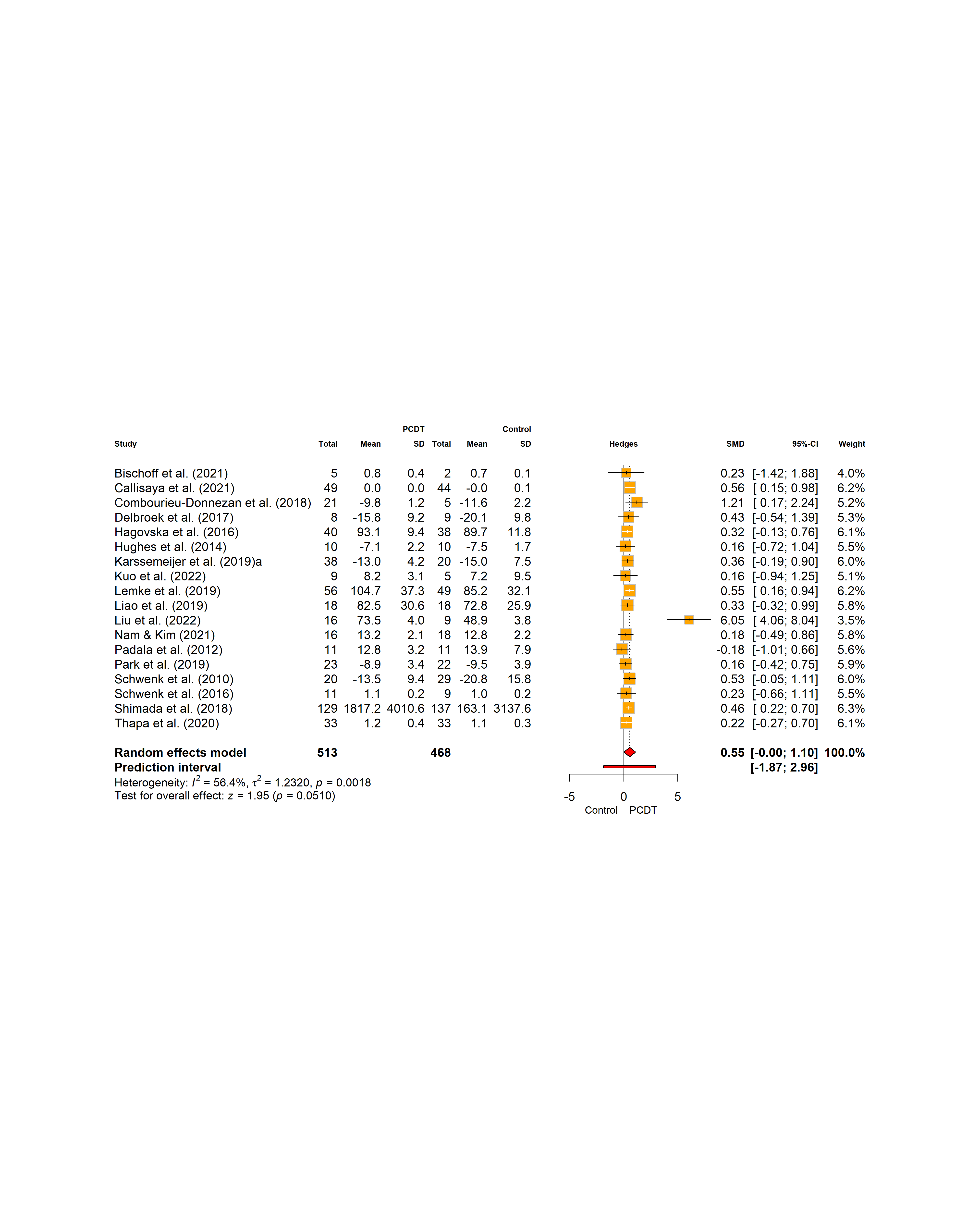


# **Appendix B5.3.** Subgroup Analyses of Forest Plot of Effect Sizes (Hedges’ g) of Study-Level Data for NCD Nature for Gait


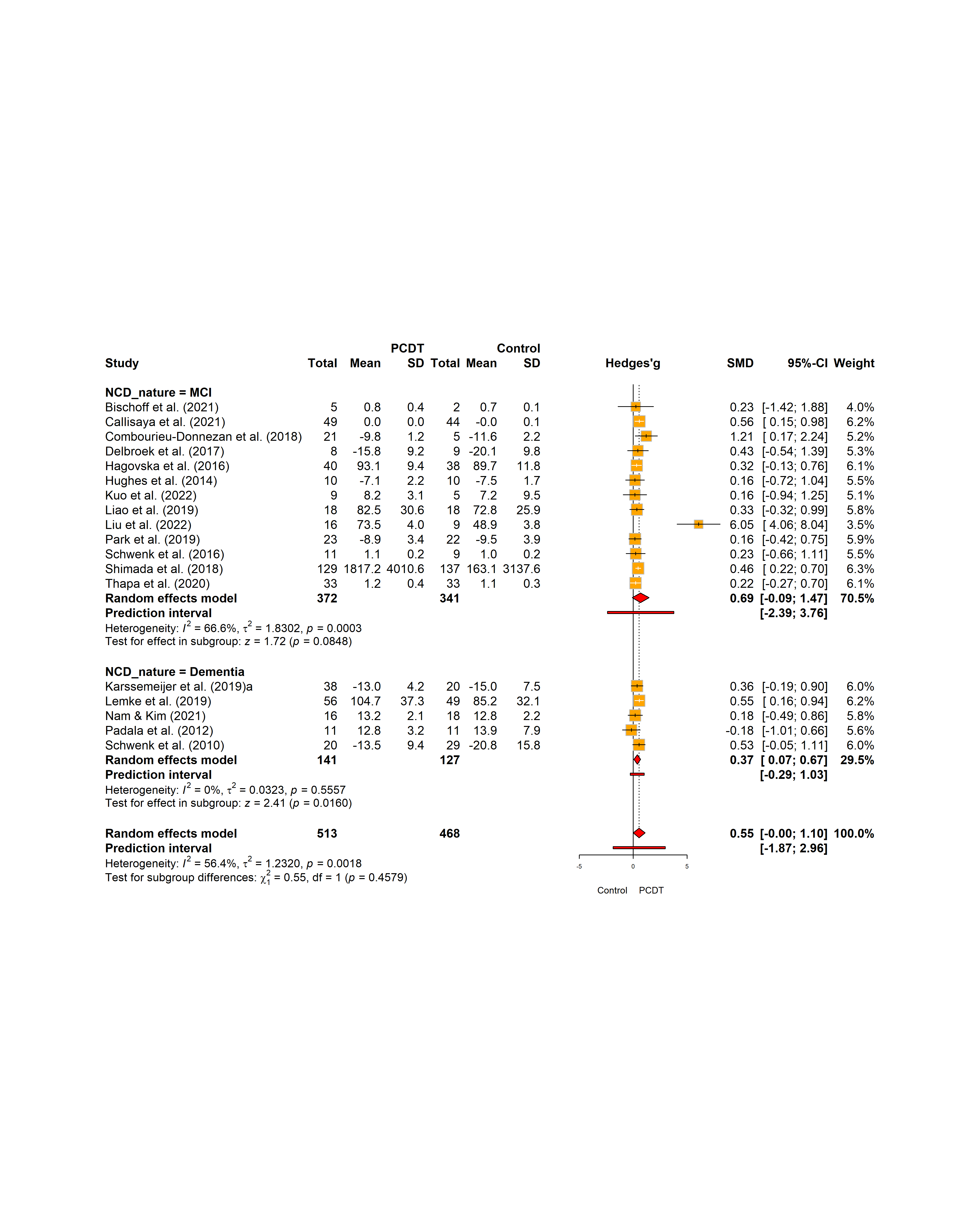


# **Appendix B5.4.** Subgroup Analyses of Forest Plot of Effect Sizes (Hedges’ g) of Study-Level Data for Intervention Type for Gait


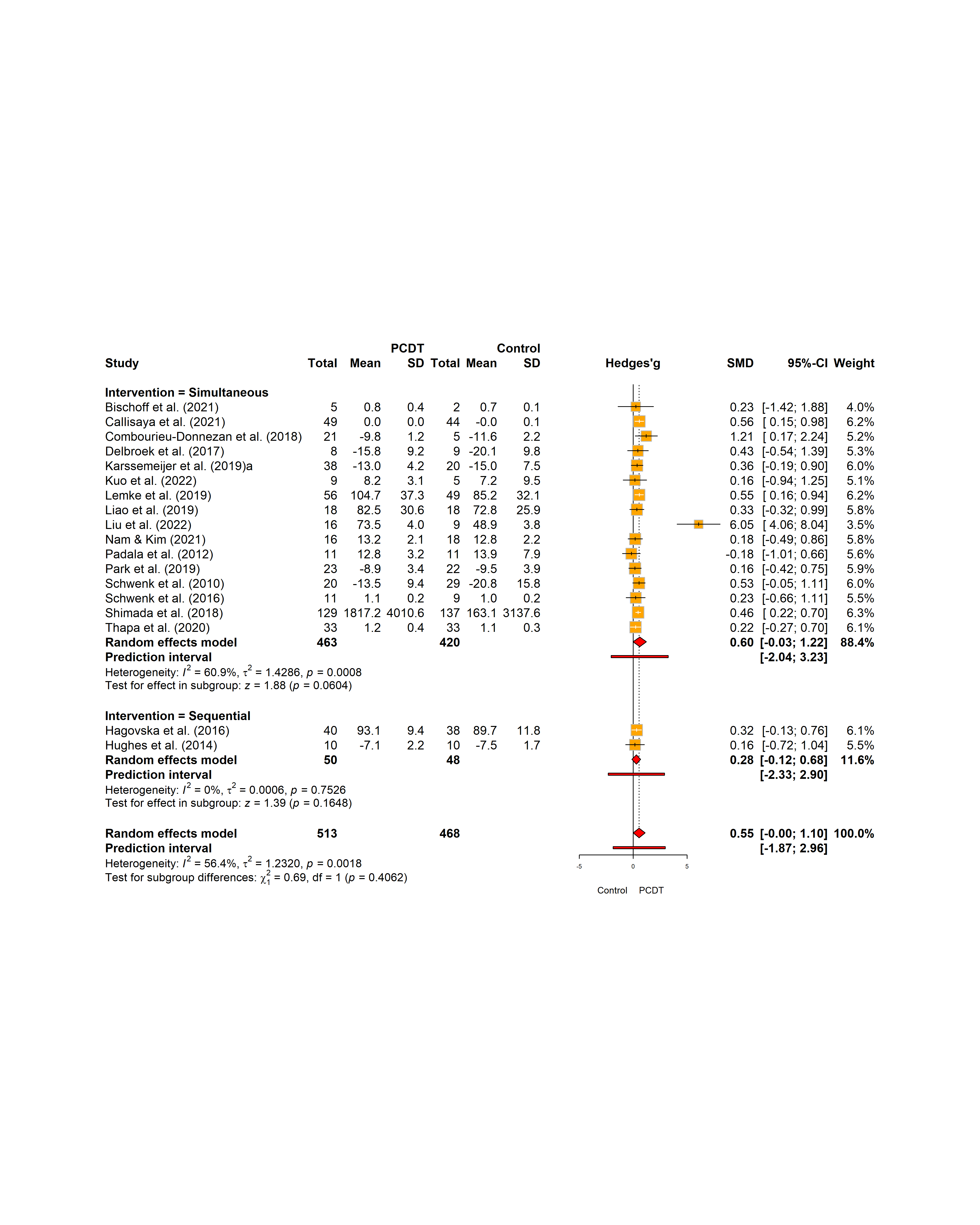


# **Appendix B5.5.** Subgroup Analyses of Forest Plot of Effect Sizes (Hedges’ g) of Study-Level Data for Training Duration for Gait


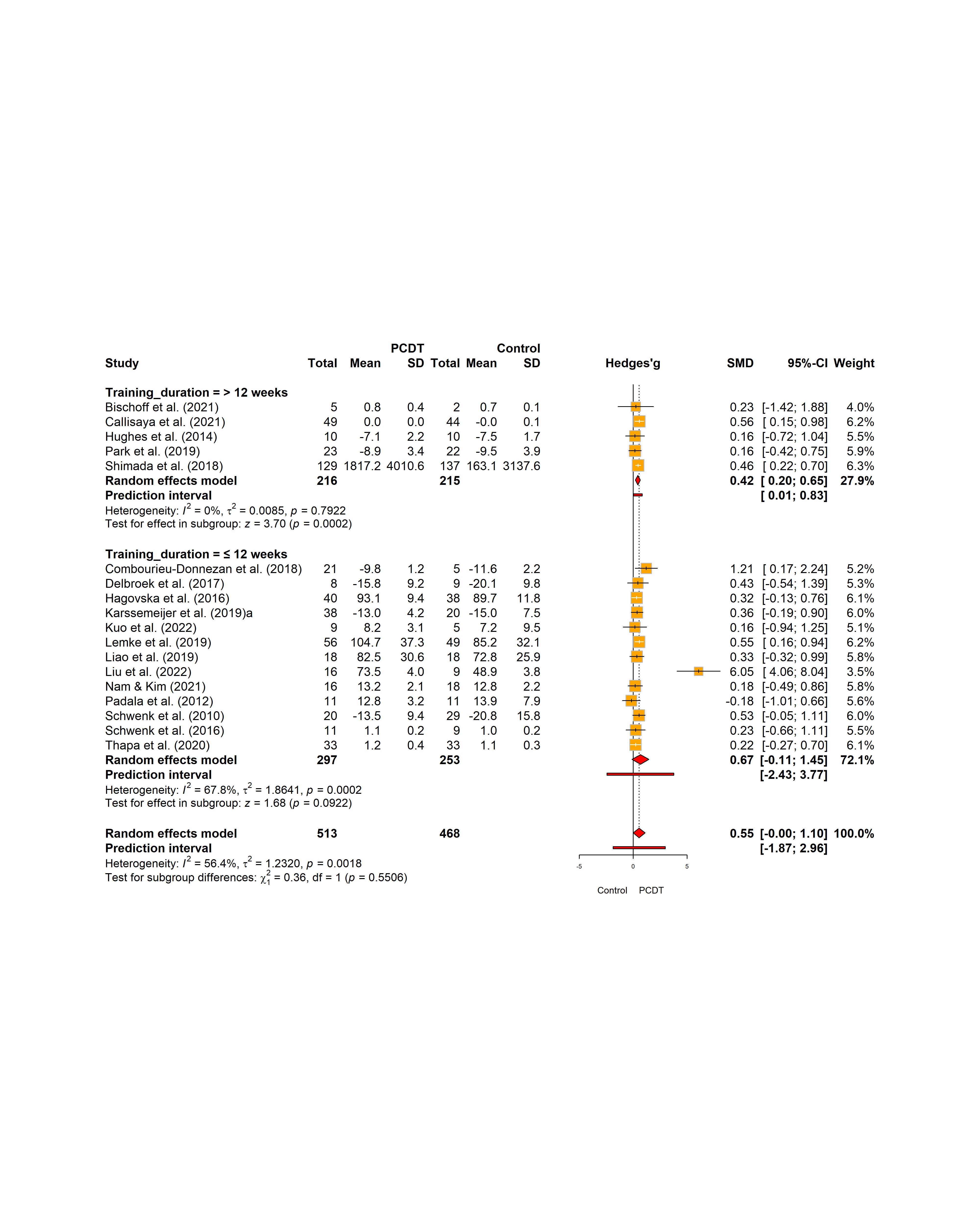


# **Appendix B5.6.** Subgroup Analyses of Forest Plot of Effect Sizes (Hedges’ g) of Study-Level Data for Session Duration for Gait


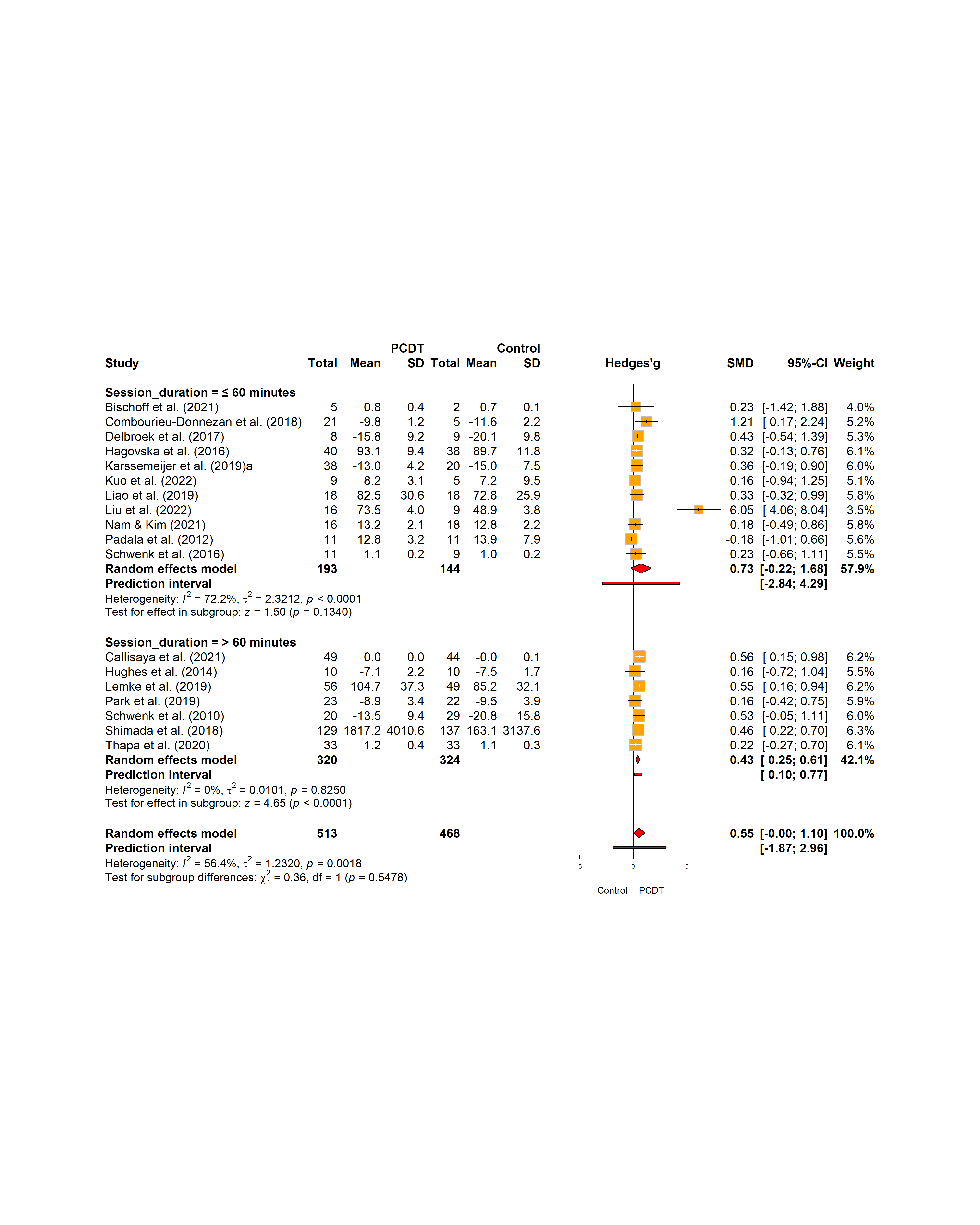


# **Appendix B5.7.** Subgroup Analyses of Forest Plot of Effect Sizes (Hedges’ g) of Study-Level Data for Training Frequency for Gait


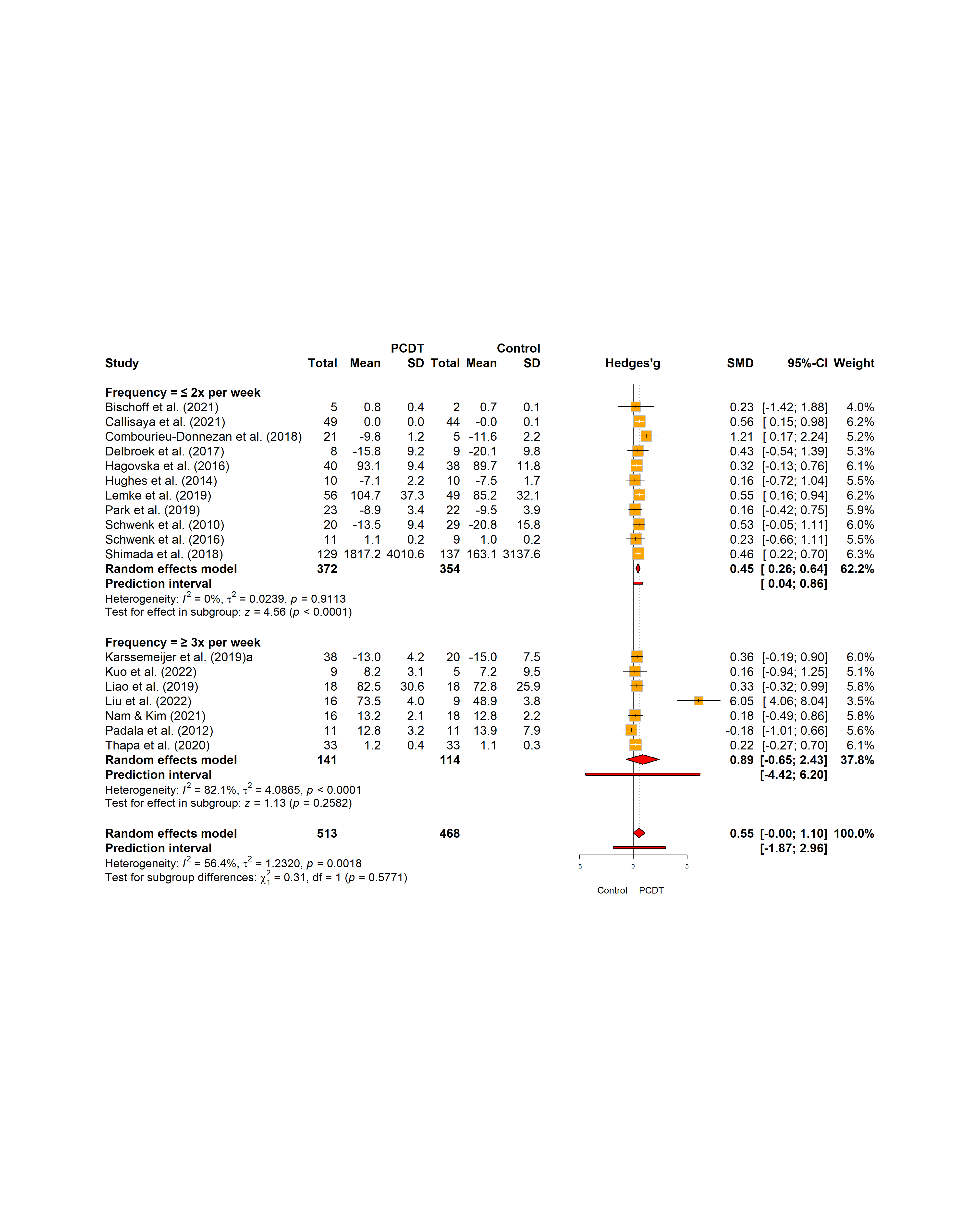


# **Appendix B6.1.** Forest Plot of Effect Sizes (Hedges’ g) of Meta-Level Data for Balance


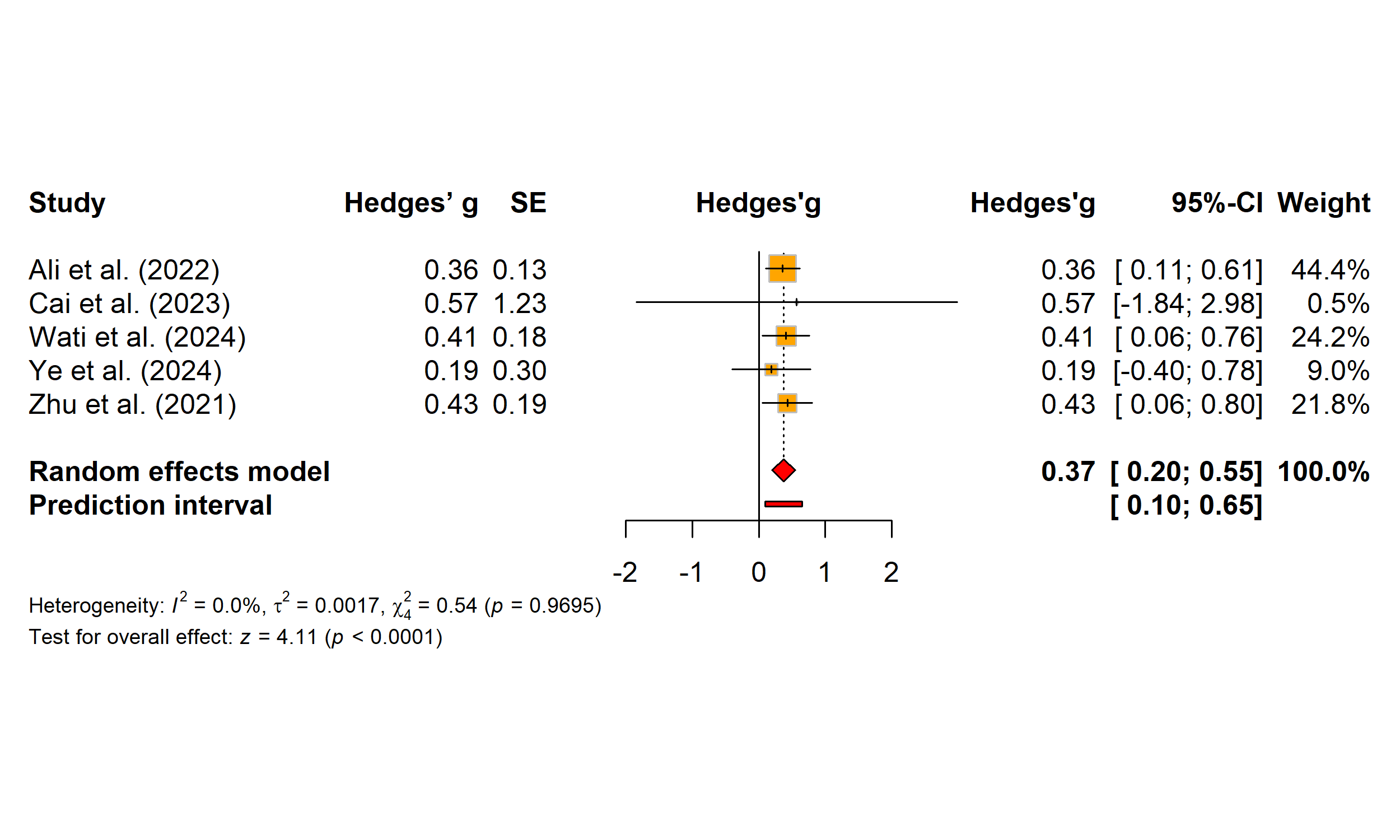


# **Appendix B6.2.** Forest Plot of Effect Sizes (Hedges’ g) of Study-Level Data for Balance


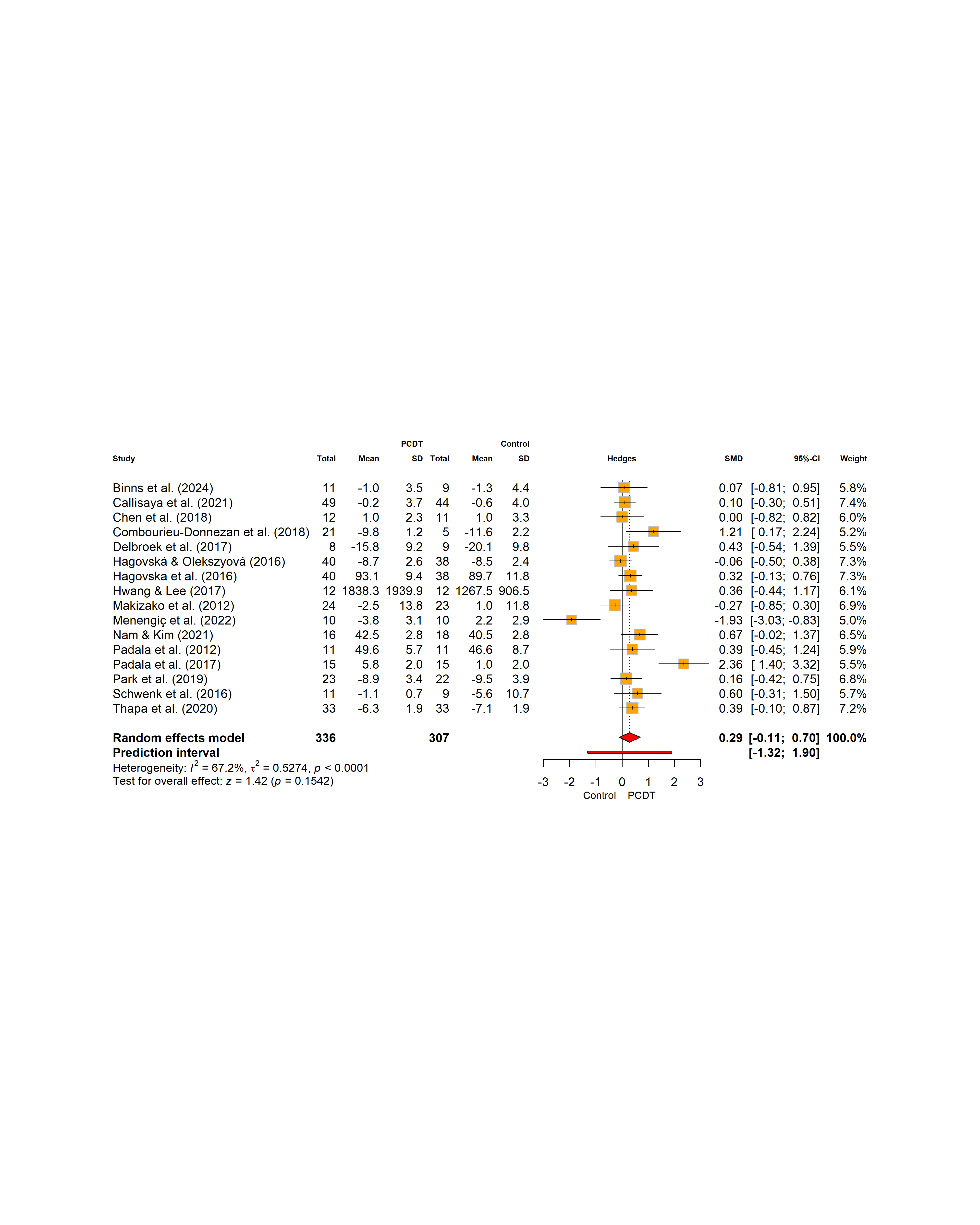


# **Appendix B6.3.** Subgroup Analyses of Forest Plot of Effect Sizes (Hedges’ g) of Study-Level Data for NCD Nature for Balance


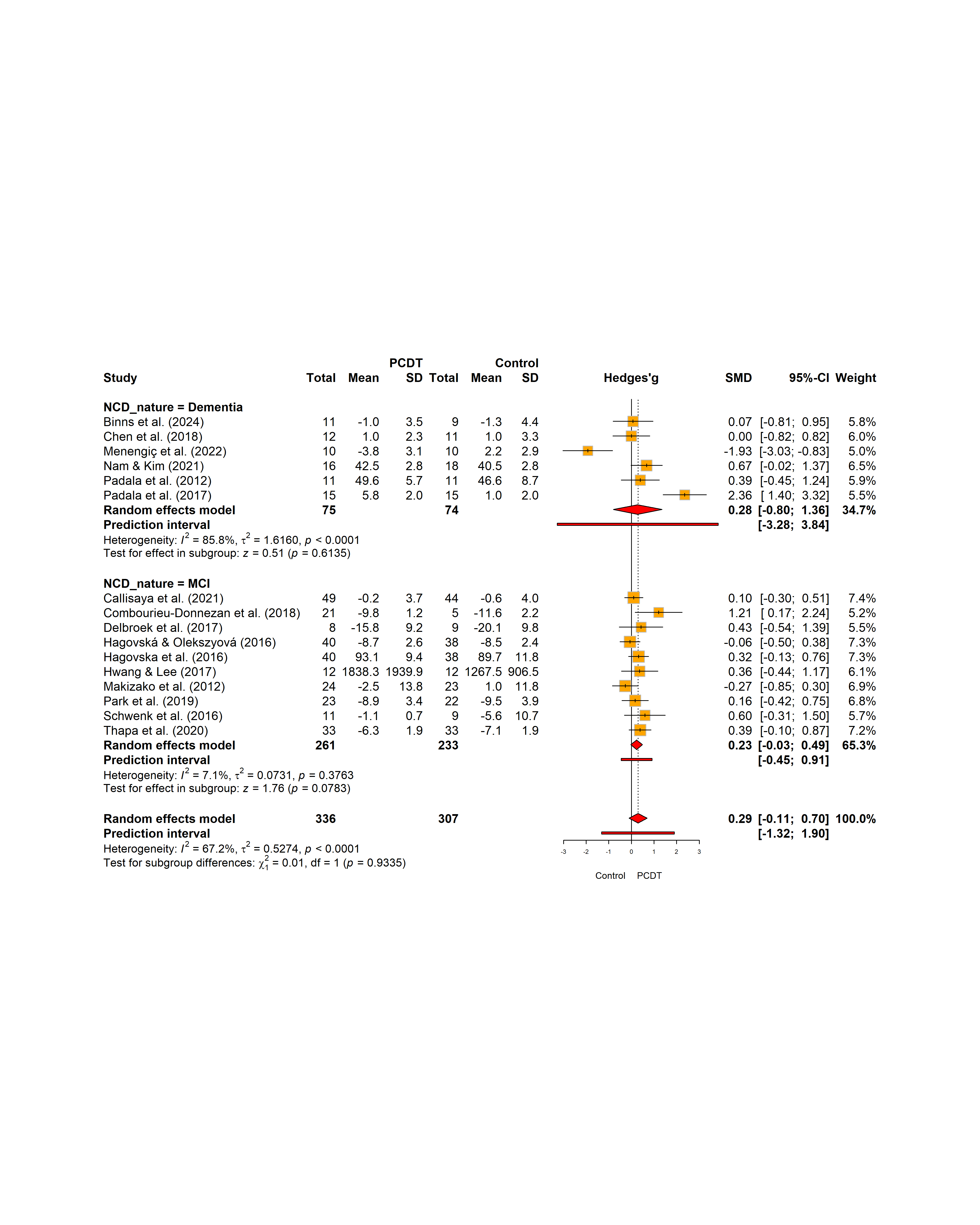


# **Appendix B6.4.** Subgroup Analyses of Forest Plot of Effect Sizes (Hedges’ g) of Study-Level Data for Intervention Type for Balance


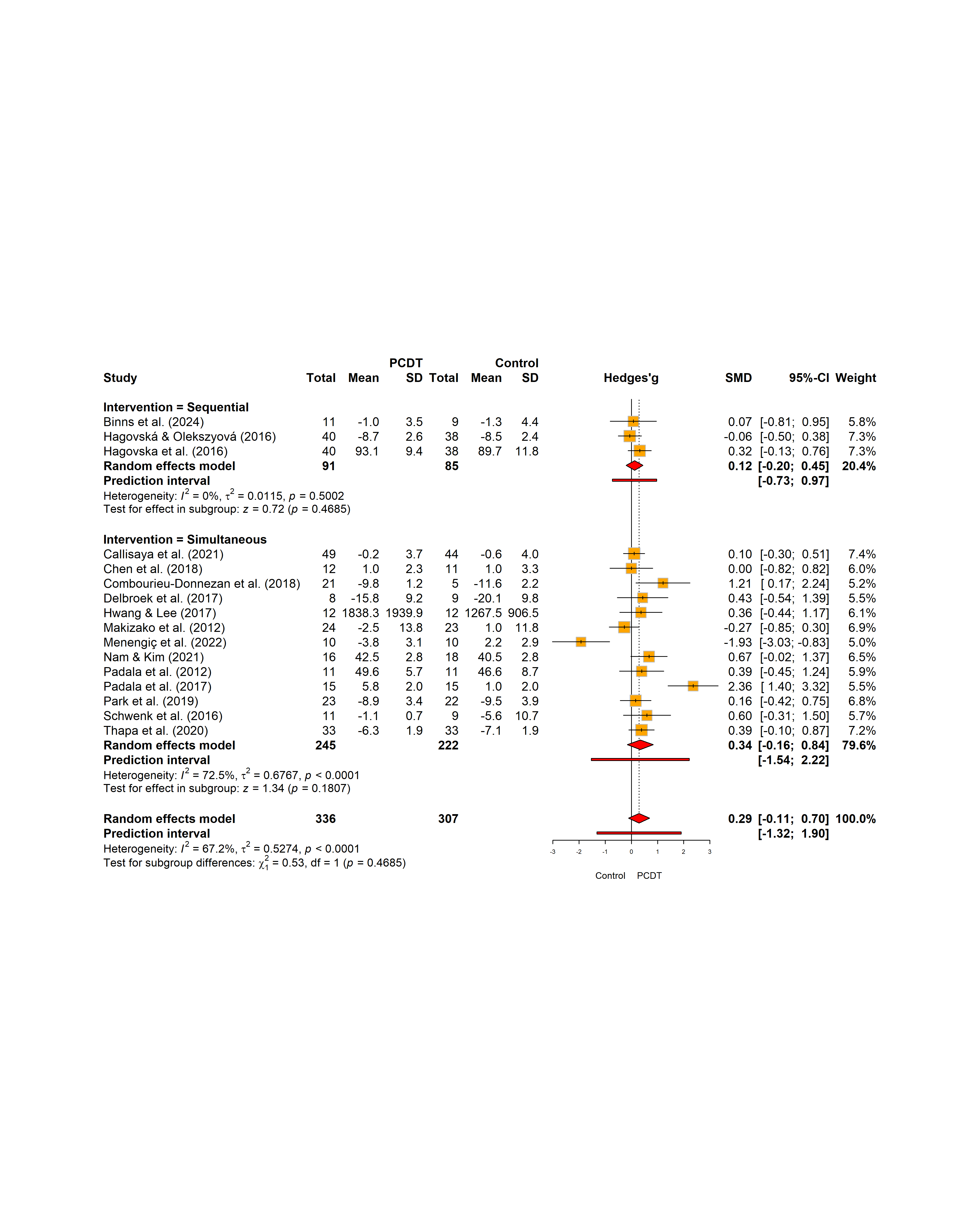


# **Appendix B6.5.** Subgroup Analyses of Forest Plot of Effect Sizes (Hedges’ g) of Study-Level Data for Training Duration for Balance


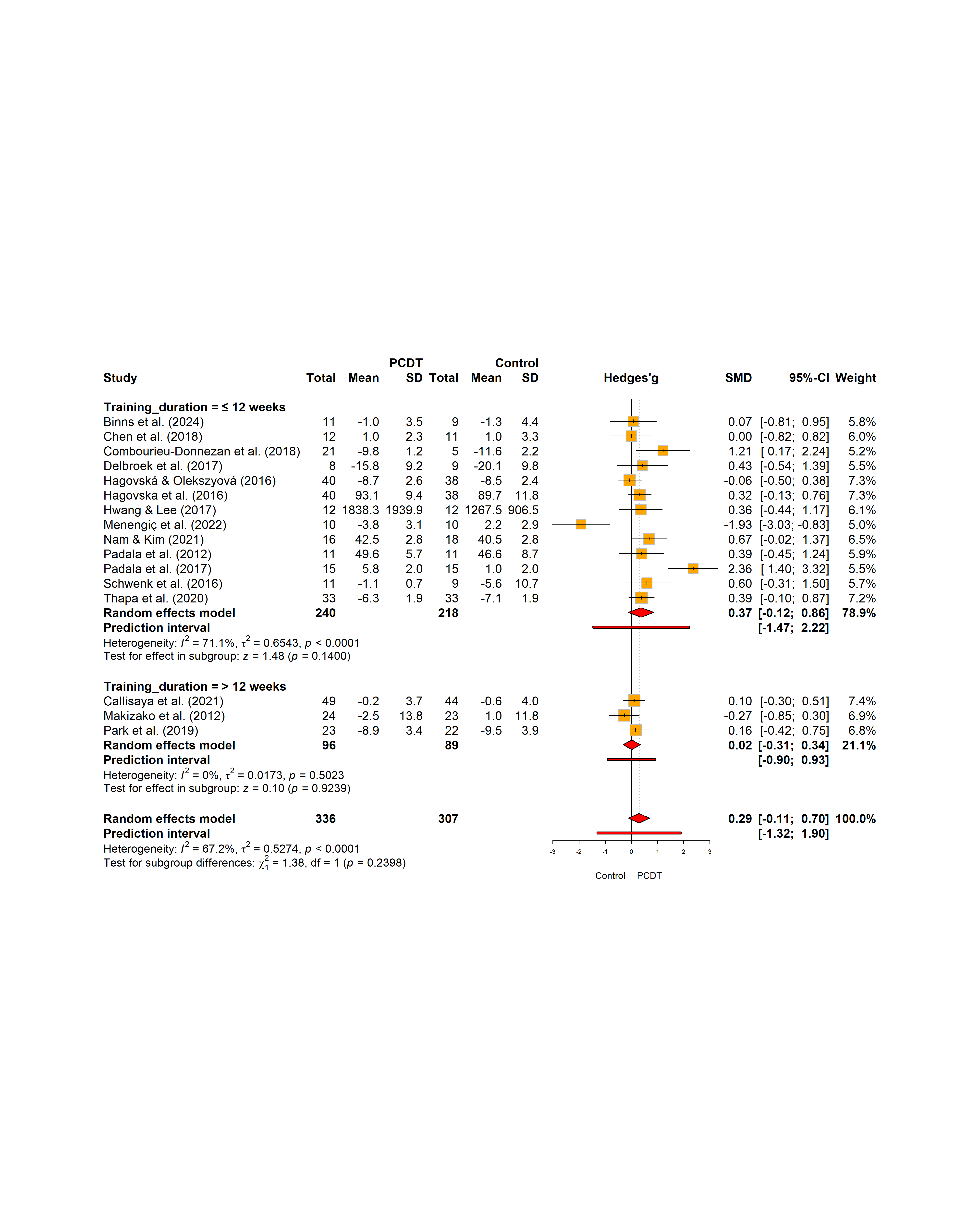


# **Appendix B6.6.** Subgroup Analyses of Forest Plot of Effect Sizes (Hedges’ g) of Study-Level Data for Session Duration for Balance


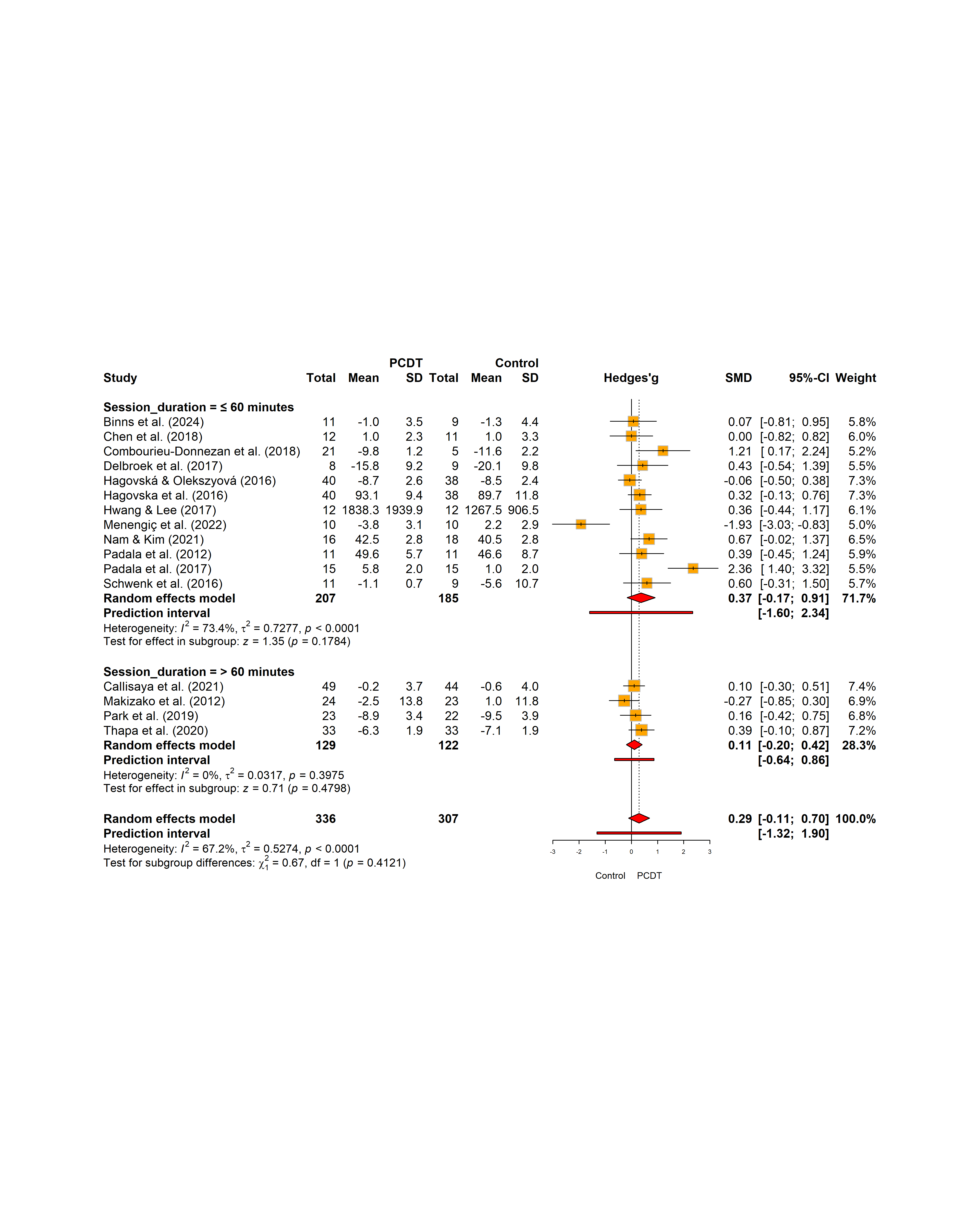


# **Appendix B6.7.** Subgroup Analyses of Forest Plot of Effect Sizes (Hedges’ g) of Study-Level Data for Training Frequency for Balance


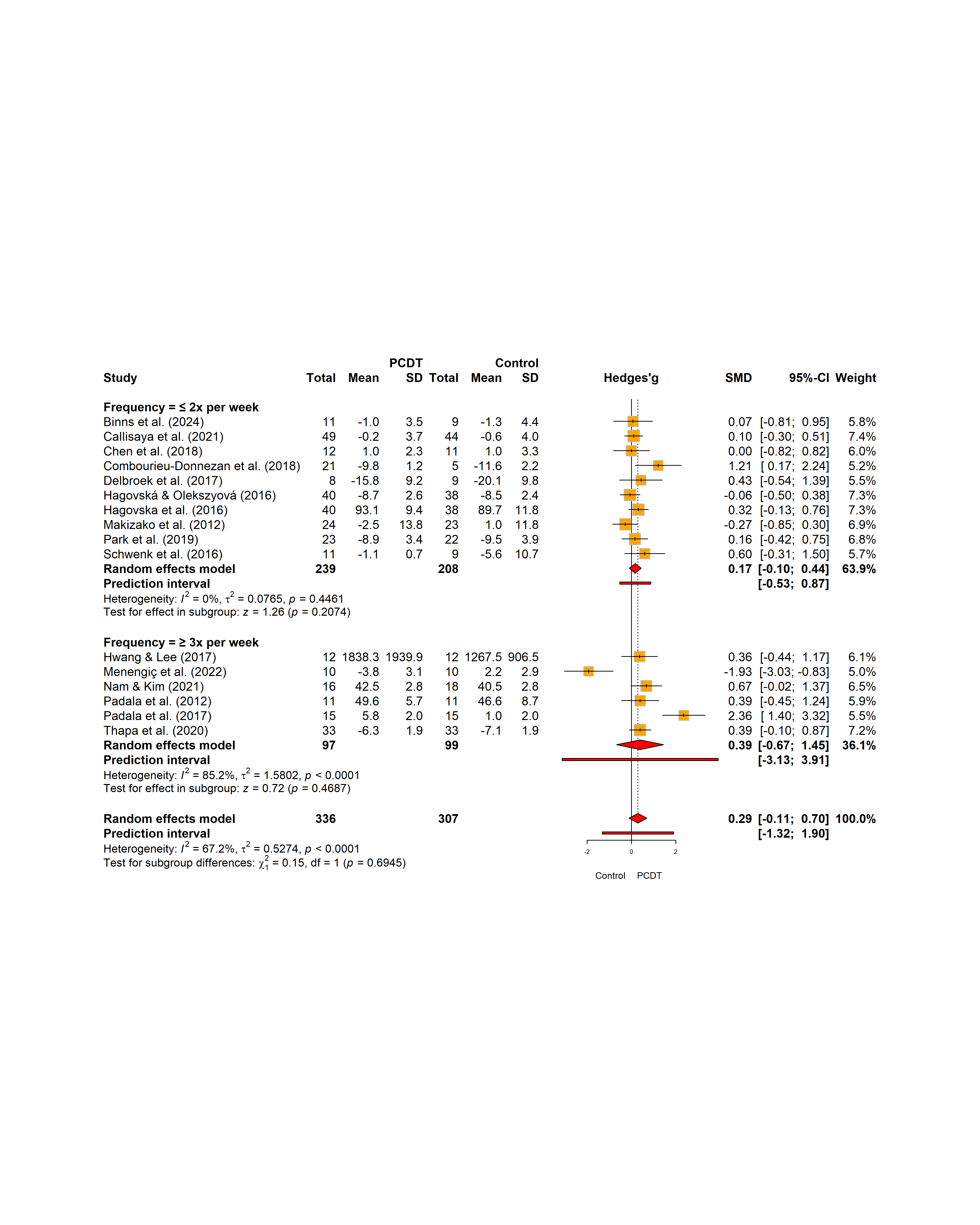


# **Appendix B7.** Funnel Plots and Publication Bias of Meta-Level Data Outcomes


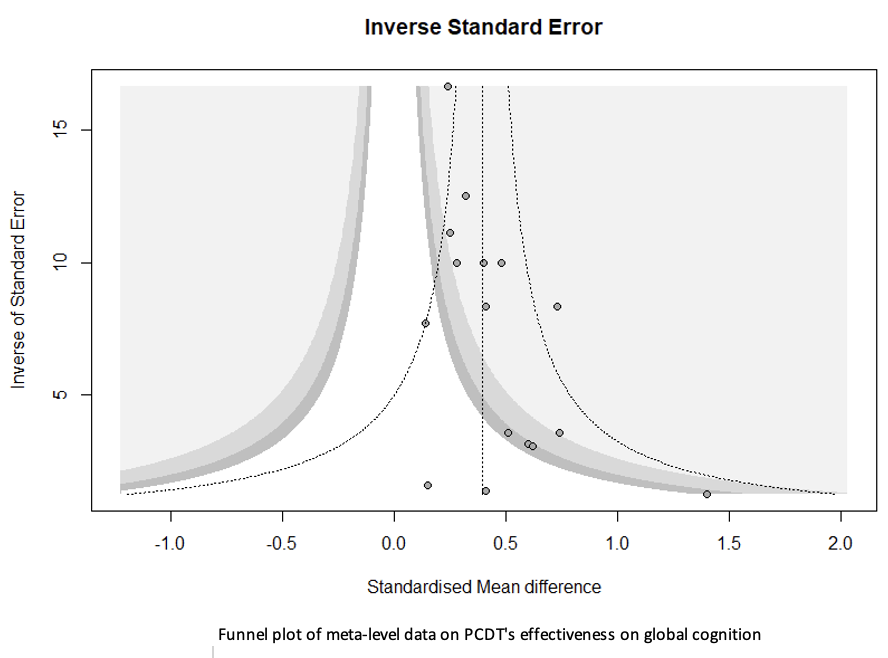

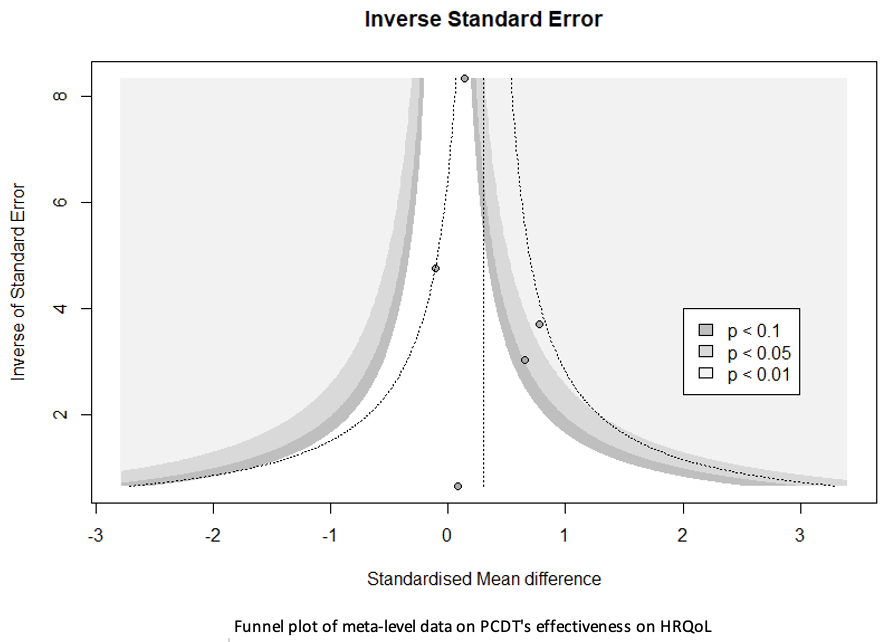


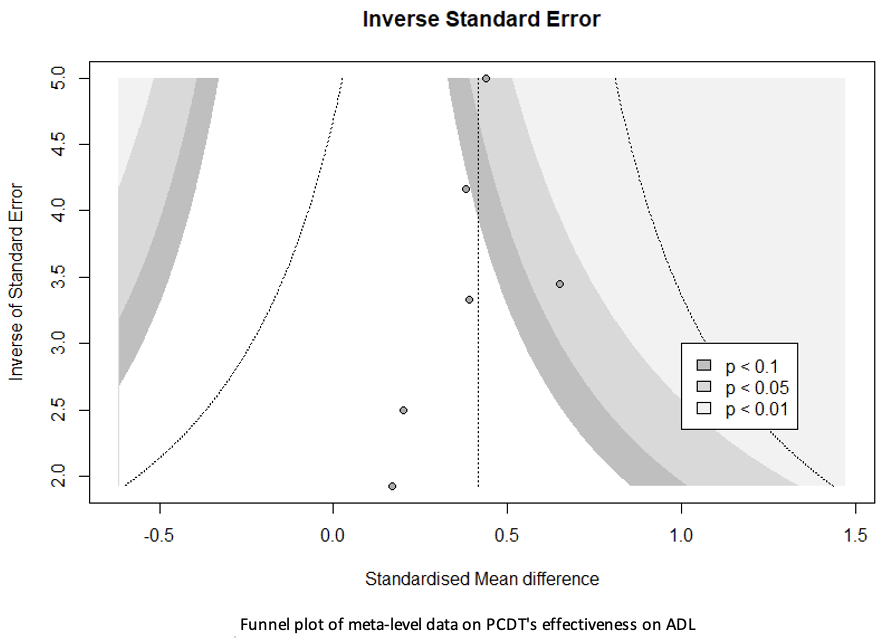

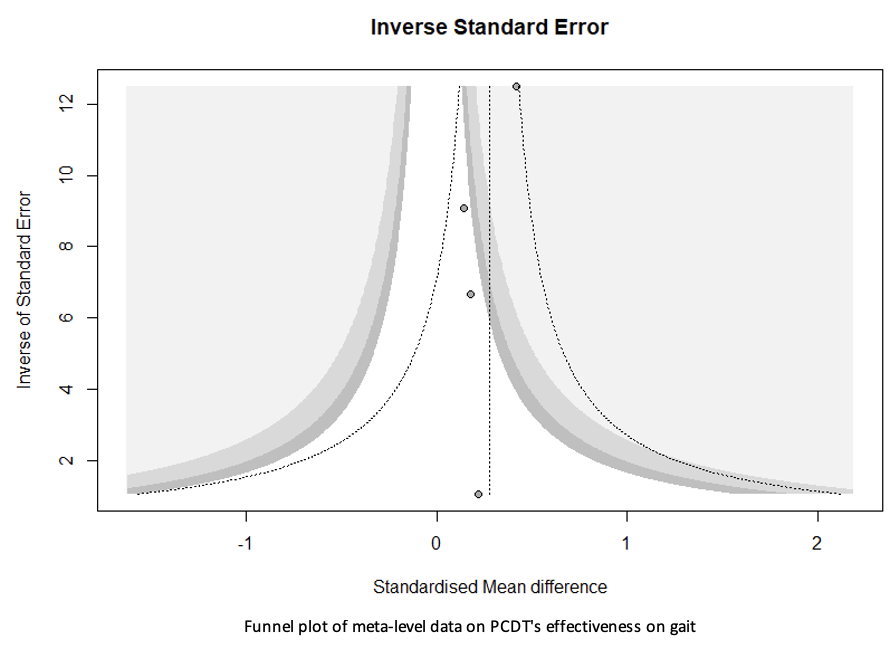


| Publication Bias Assessment | | |
| --- | --- | --- |
| Outcome | Egger’s Regression value | *p*-value |
| Global Cognition | 1.08 | 0.07 |
| HRQoL | 0.96 | 0.53 |
| ADL | -0.77 | 0.32 |
| Gait | -0.86 | 0.62 |
| Balance | -0.05 | 0.91 |


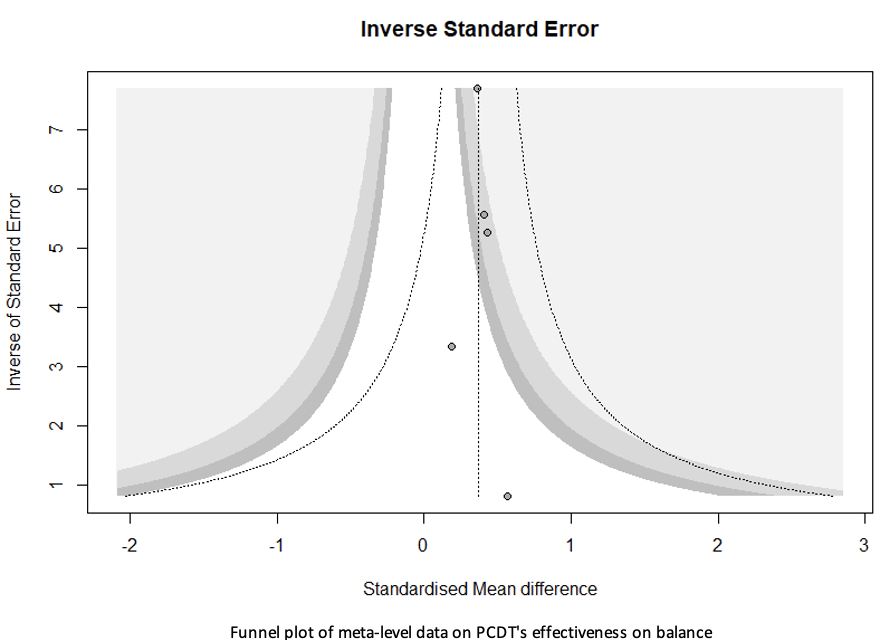


# **Appendix B8.** Funnel Plots and Publication Bias of Study-Level Data Outcomes


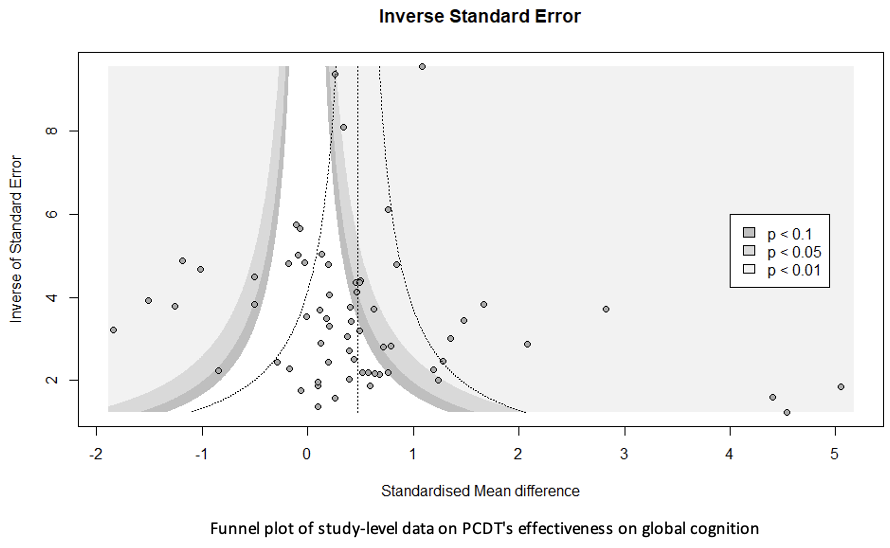

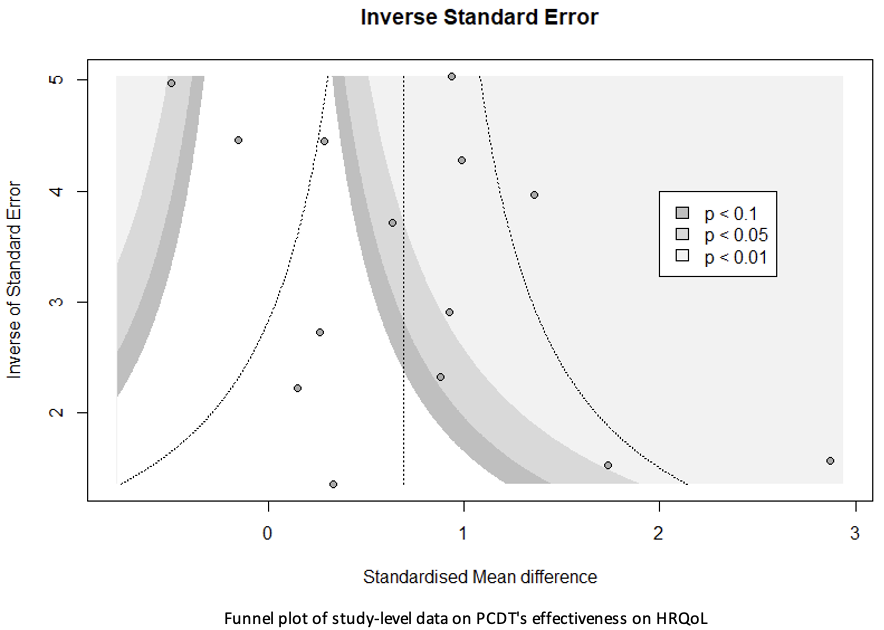


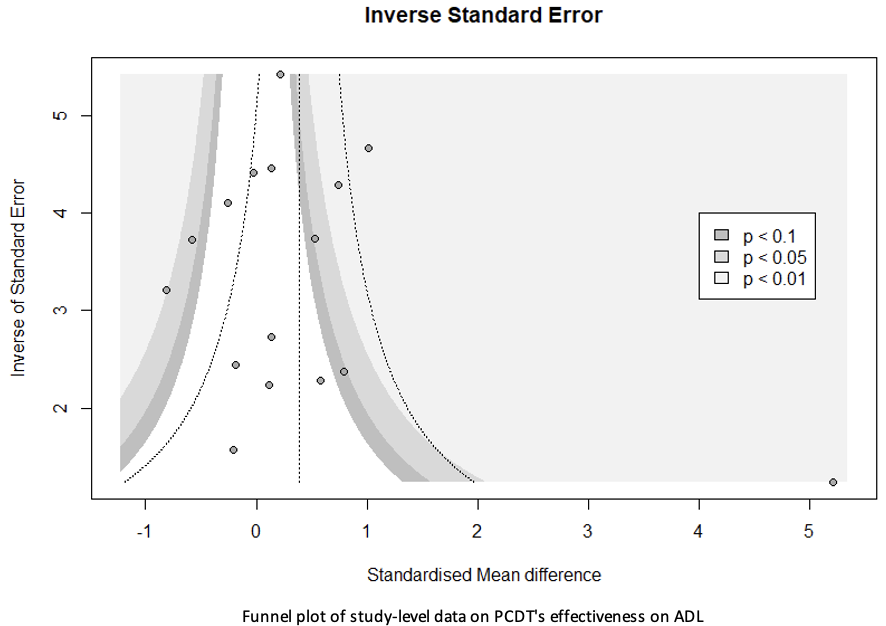

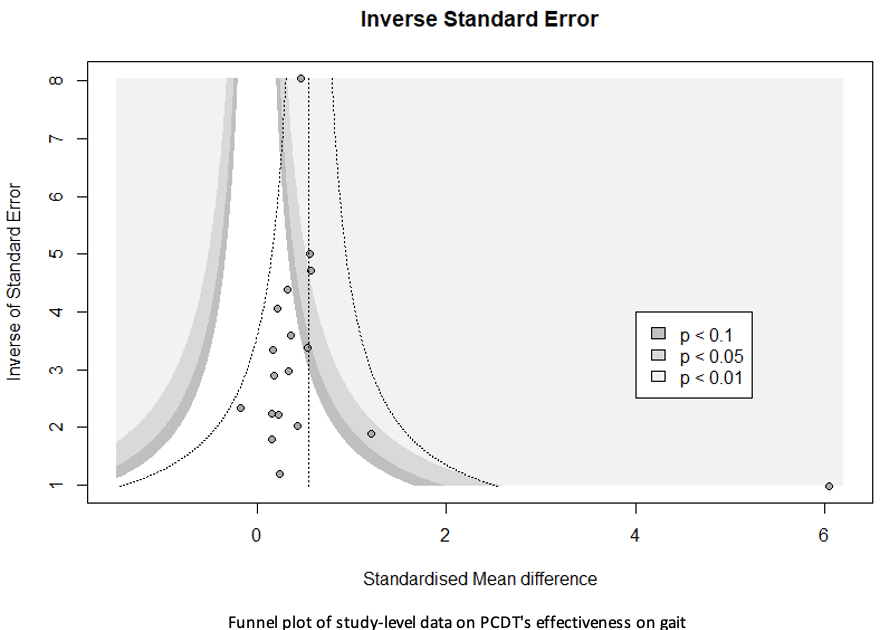


| Publication Bias Assessment | | |
| --- | --- | --- |
| Outcome | Egger’s Regression value | *p*-value |
| Global Cognition | 0.94 | 0.33 |
| HRQoL | 2.35 | 0.20 |
| ADL | 2.45 | 0.03 |
| Gait | 0.69 | 0.39 |
| Balance | 1.05 | 0.46 |


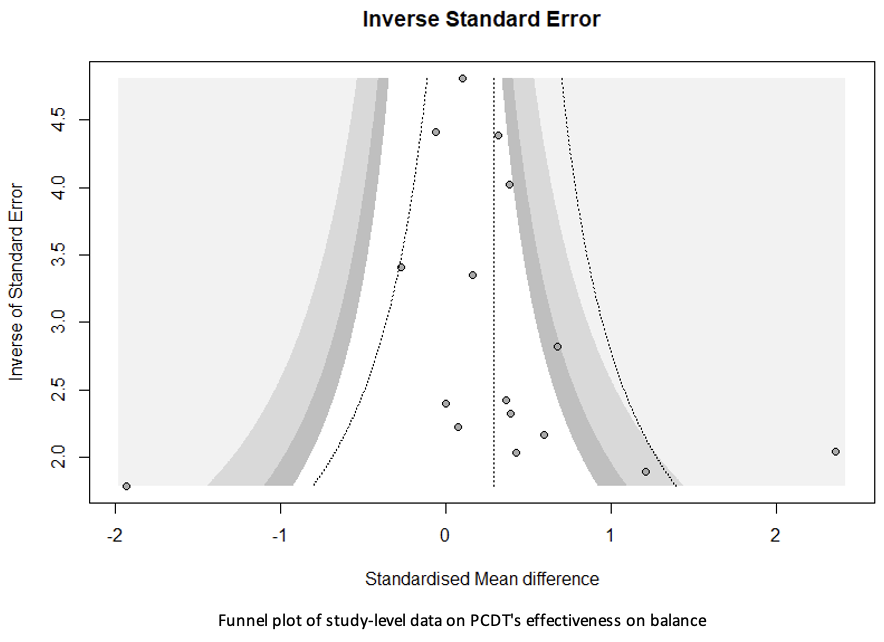

Supplement: aa-25-2382-File003_afag061 [file aa-25-2382-file003_afag061.docx]
